# Upregulation of Yin-Yang-1 Associates with Proliferation and Glutamine Metabolism in Esophageal Carcinoma

**DOI:** 10.1155/2022/9305081

**Published:** 2022-03-20

**Authors:** Can Luo, Xin Chen, Yuting Bai, Lei Xu, Shuqi Wang, Lihua Yao, Xiaolan Guo, Dongsheng Wang, Xiaowu Zhong

**Affiliations:** ^1^Department of Clinical Laboratory, Affiliated Hospital of North Sichuan Medical College, Nanchong, 637000 Sichuan, China; ^2^Translational Medicine Research Center, North Sichuan Medical College, Nanchong, 637000 Sichuan, China; ^3^Department of Laboratory Medicine, North Sichuan Medical College, Nanchong, 637000 Sichuan, China; ^4^Department of Rehabilitation Medicine, Affiliated Hospital of North Sichuan Medical College, Nanchong, 637000 Sichuan, China; ^5^University of Electronic Science and Technology of China, Chengdu, 610041 Sichuan, China; ^6^Department of Clinical Laboratory, Sichuan Cancer Hospital & Institute, Chengdu, 610041 Sichuan, China

## Abstract

**Objective:**

To investigate the expression of Yin-Yang-1 (YY1) in esophageal carcinoma (ESCA) and its effect on glutamine metabolism in ESCA.

**Methods:**

The expression and roles of YY1 in ESCA were investigated using a series of bioinformatics databases and tools. The expression of YY1 between ESCA tissues with the corresponding adjacent tissues was validated using real-time PCR, western blot, and immunohistochemical staining method. Furthermore, the effects of YY1 on ESCC cell proliferation and migration were examined. The correlation between the YY1 and glutamine metabolism was evaluated by western blot.

**Results:**

YY1 gene was highly conserved in evolution and upregulated in ESCA tissues and ESCC cell lines (ECA109 and TE-1). In addition, YY1 may affect the level of immune cell infiltration and promote tumor cell immune escape. Functional enrichment analysis found that YY1 involved in many biological processes, such as cell division and glutathione and glutamine metabolism. After siRNA knockdown of YY1 in ECA109 and TE-1, the proliferation and the migration of ECA109 and TE-1 were suppressed. The glutamine consumption and glutamate production were significantly decreased. The protein expression of alanine-, serine-, cysteine-preferring transporter 2 (ASCT2), glutaminase (GLS), and glutamate dehydrogenase (GLUD1) was significantly downregulated.

**Conclusion:**

YY1 is highly expressed in ESCA and may promote glutamine metabolism of ESCC cells, indicating it may be as a diagnostic biomarker for ESCA.

## 1. Introduction

Esophageal carcinoma (ESCA) is one of the most common cancers in the world, ranking seventh in morbidity and sixth in mortality among all cancers [1]. More than 500,000 people die from ESCA each year, accounting for 5.3% of all cancer-related deaths worldwide. In addition, it also shows significant geographical differences. China has the highest morbidity and mortality of ESCA in the world [2], while North Sichuan is one of the areas with high incidence of ESCA in China [3]. ESCA is mainly divided into two pathological types: esophageal squamous cell carcinoma (ESCC) and esophageal adenocarcinoma (EAC). At present, endoscopy and surgery are often used in the early stage of ESCA, while the treatment of advanced ESCA with radiotherapy, neoadjuvant chemotherapy, and esophagectomy is done according to the actual situation [4]. Many studies have found that there are a variety of gene mutations in ESCA, including TP53, NOTCH1, NOTCH2, and CCND1, which may be used as a diagnostic biomarker for patients with ESCA [5–7]. But these are no specific diagnostic indexes, the early diagnosis rate of ESCA patients is low. Moreover, the middle and late ESCA are more invasive and easier to relapse and metastasize distally [8]. These characteristics make the follow-up treatment and prognosis of ESCA not ideal. The 5-year survival rate of ESCA with local metastasis and distant metastasis is 25.1% and 4.8%, respectively [9]. The incidence of ESCA is high in the world and increasing year by year [10]. Therefore, the exposition of the carcinogenic effect of ESCA and the identification of new biomarkers or therapeutic targets may be very useful for optimizing the current treatment of ESCA.

Transcription factor Yin-Yang-1 (YY1) [11] is a multifunctional zinc finger protein of the yin-yang family, which has a highly conserved C2H2 zinc finger transcription factor in all vertebrates. YY1 is widely expressed in human tissues and participates in the regulation of a variety of molecular mechanisms, including transcriptional regulation and epigenetic modification [12]. YY1 can regulate the transcription of gene expression and participates in embryonic development, cell proliferation and differentiation [13]. Studies have found that YY1 is highly expressed in many cancers, participates in the regulation of tumor proliferation and growth, and plays a dual role in inhibiting or promoting tumor progression [12]. For example, YY1 inhibits tumor proliferation in colon cancer and breast cancer [14] but promotes tumor growth in melanoma, non-Hodgkin Lymphomas [15], and prostate cancer [12]. The expression of YY1 is significantly increased in colon cancer and subjected to O-GlcNAcylation modifications, which increases the stability and transcriptional activity of YY1, thus promoting tumor proliferation [16]. In addition, YY1 regulates the upregulation of metabolite transporter expression, which in turn promotes the survival and proliferation of colon cancer cells [17]. The signal pathways related to lung cancer progression can upregulate the expression of YY1 in lung cancer, while the overexpression of YY1 can lead to cancer progression by positively regulating the expression of many oncogenes [18]. YY1 has been shown to inhibit tumor suppressor and thus promote the proliferation of prostate cancer cells [19]. Recent studies have shown that YY1 is also involved in tumor metabolic reediting. YY1 can regulate glucose uptake, pentose phosphate pathway, and lipid metabolism of tumor cells by regulating peroxisome proliferator-activated receptor gamma coactivator-1 *β* (PGC-1*β*) [20], a key enzyme of lipid metabolism, and glucose-6-phosphatedehydrogenase (G6PD) [21], which regulates the key rate-limiting enzymes of glucose metabolism, that leads to metabolic reprogramming of tumor cells to promote tumor cell growth and metabolism. During tumorigenesis, the change of glutamine metabolism is also a characteristic of abnormal tumor metabolism [22]. Glutamine metabolism can provide energy and biological macromolecular materials to meet the needs of rapid growth and proliferation of tumor cells and play an important role in the maintenance of intracellular redox homeostasis and intracellular signal transduction. As we know, glutamine metabolism plays a regulatory role in the occurrence and development of tumor [23]. Abnormal glutamine metabolism mediated by oncogenes in tumor cells has become a new way of energy supply after glucose and fatty acid metabolism [24]. However, whether YY1 is involved in the regulation of glutamine metabolism remains to be further discussed.

The purpose of this study was to analyze the expression and function of YY1 in ESCA and its relationship with clinicopathological parameters based on the TCGA database. The possible molecular mechanism of YY1 in ESCA was discussed from the aspects of gene mutation, immune cell infiltration, and functional annotation analysis. Finally, the effects of YY1 on cell proliferation and glutamine metabolism of ESCC cell lines (ECA109 and TE-1) were analyzed, and its potential molecular mechanism was discussed to provide a theoretical basis for ESCA to develop new clinical therapeutic strategies.

## 2. Materials and Methods

### 2.1. Clinical Specimen

31 cases of pathologically confirmed ESCA tissues and matched paracancerous tissues were collected from the Department of Cardiothoracic surgery of the Affiliated Hospital of North Sichuan Medical College. The study is in line with the Helsinki Declaration, approved by the Medical Ethics Society of the Affiliated Hospital of North Sichuan Medical College, and all sampled patients have informed consent.

### 2.2. Expression and Clinicopathological Analysis of YY1

TIMER (https://cistrome.shinyapps.io/timer/) [25] is a systematic database that uses microarrays to calculate gene expression characteristics. In this study, the mRNA expressions of YY1 in pancancer tissues with their corresponding adjacent normal control samples were analyzed by the TIMER database. UALCAN (http://ualcan.path.uab.edu) [26] uses TCGA level 3 RNA-seq and clinical data from 33 cancer types. In this study, the mRNA expressions of YY1 of ESCA tissues and normal tissues were analyzed in the TCGA-ESCA dataset. Meanwhile, the association between the mRNA expression of YY1 in ESCA tissues with their clinicopathologic parameters such as individual cancer stages, patient's race, nodal metastasis status, patient's age, TP53 mutation status, and tumor histology were analyzed by the UALCAN database.

### 2.3. Diagnostic Analysis

The expression data and mRNA expression profiles of ESCA patients were downloaded from the TCGA database, including 11 samples of normal esophageal tissues and 162 ESCA tissues (Workflow type: HTSeq-FPKM). The clinical characteristic data of patients with ESCA from TCGA was also collected. The ROC curve (receive operating characteristic curve) was plotted using R. The cut-off value of the YY1 expression was determined by the middle bit value.

### 2.4. cBioPortal Analysis

cBioPortal (https://www.cbioportal.org/) [27] has multidimensional cancer genomics datasets. The cBioPortal was used to explore, visualize, and analyze genetic alterations analysis and survival analysis of YY1 in the esophageal carcinoma (TCGA, Firehose legacy) dataset. The genomic profiles were consisted of mutations and putative copy number alterations (CNA).

### 2.5. Immune Infiltration Analysis

In this study, TIMER was used to explore the correlation between the expression level of YY1 and the abundance of immune cell infiltration and evaluate the effect of YY1 gene somatic cell copy number change (SCNAs) on immune cell infiltration. Furthermore, GEPIA2 (http://gepia2.cancer-pku.cn/#index) [28] is an interactive web that includes 9,736 tumors and 8,587 normal samples from TCGA and the GTEx projects. We analyzed the association of YY1 expression with gene biomarkers of immune cell in ESCA tumor, ESCA normal, and GTEx via GEPIA2. The association of YY1 expression with immune subtype and ESCA-related chemokines was investigated using TISIDB (http://cis.hku.hk/TISIDB/) [29].

### 2.6. YY1-Related Gene Enrichment Analysis

LinkedOmics (http://www.linkedomics.org/) [30] is a web-based platform for analyzing 32 TCGA cancer-associated multidimensional datasets. The TCGA ESCA dataset was used to analyze the YY1-related expression gene. The volcano plots and heat maps were explored statistically using Spearman's correlation coefficient. Furthermore, the biological functions of these genes were classified by DAVID analysis tool (https://david.ncifcrf.gov/) [31]. The biological process (BP), molecular function (MF), cellular component (CC), and KEGG pathway were selected as analysis items.

### 2.7. Real-Time PCR

Total RNA was extracted from tissues and cells using TRIzol (Ambion, Carlsbad, USA). Reverse transcription was conducted with Transcriptor First-Stand cDNA Synthesis Kit (Roche, Balser, USA), and cDNA was collected. Fast SYBR Green Master mix (Takara, Tokyo, Japan) was used to detect YY1 mRNA expression levels by real-time PCR with LightCycler 96 system (Roche, Balser, USA). The reaction conditions of PCR were predenatured at 95°C for 10 min, denatured at 95°C, 30 s at 60°C, 30 s at 72°C, and extended for 30 s. YY1 and *β*-actin primers (the sequence is shown in Table S1) were provided by Sangon Biotech (Shanghai, China). Each experiment was performed in triplicate. Data are analyzed using the 2^-*ΔΔ*Ct^ method with the normalization to *β*-actin [20].

### 2.8. Western Blot

Total proteins were extracted from tissues or cells using a precooled RIPA buffer containing protease and phosphatase inhibitors (Beyotime, Shanghai, China). The protein concentration was determined by BCA protein concentration determination kit (Thermo Fisher, Massachusetts, USA). The same amount of protein samples was separated with 10%SDS-PAGE (EpiZyme, Shanghai, China) and then transferred to the PVDF membrane (Millipore, Billerica, USA). After sealing with 5% skim milk in PBST for 1 h, the membrane and corresponding YY1 antibody (catalog number: ET1605-40, dilutions: 1 : 2000, HUABIO, Hangzhou, China) were incubated overnight at 4°C. After washing by PBST, we put the PVDF membrane into the corresponding species diluted secondary antibody (catalog number: HA1025, dilutions: 1 : 5000, HUABIO, Hangzhou, China) incubated at room temperature for 1 h. Western blotting was detected by the imaging system (Vilber, Paris, France) using the ECL chemiluminescence chromogenic solution (Millipore, Billerica, USA). GAPDH were selected to be the loading controls [21].

### 2.9. Immunohistochemical Staining

The expression of YY1 protein in ESCA tissues was detected strictly according to the instructions of Zhongshan Jinqiao immunohistochemical kit (ZSJQ BIO, Beijing, China). The paraffin-embedded tissue specimens were continuously sliced with a thickness of about 4 *μ*m. The slices were dewaxed with xylene and dewaxed with gradient ethanol. The slices were placed in the repair box of citric acid antigen repair buffer and repaired in the microwave oven. The 8 min was boiled over medium heat, and then, the ceasefire 8 min was transferred to medium and low heat for 7 min. The 25 min was incubated with hydrogen peroxide solution of 3% hydrogen peroxide at room temperature and incubated with light. After the serum sealed 30 min, we add YY1 antibody (catalog number: ET1605-40, dilutions: 1 : 100, HUABIO, Hangzhou, China) overnight at 4°C. Then, PBS was washed for 3 times and the corresponding species second antibody (catalog number: SV0002, BOSTER, Wuhan, China) was added. After incubating at room temperature for 50 min, the chromogenic agent, hematoxylin restaining, blue return, dehydration, transparent, and neutral resin seal were used [32].

### 2.10. Transient Cell Transfection

Small interfering RNAs (siRNA) directed against YY1 (siRNA-YY1-1 and siRNA-YY1-2) and negative control RNAs (siRNA-NC) were synthesized by Ribobio (Ribobio, Guangzhou, China). ESCC cell lines (ECA109 and TE-1) in the logarithmic growth phase were counted and inoculated in six-well plate with 5 × 10^5^ cells per well. Transfection was carried out when the cell density was 50%-60%. Cells were transfected with plasmids by Lipofectamine 2000 reagent (Invitrogen, Waltham, MA) according to the manufacturer's protocol. Cells were harvested after 48 hours for immunoblot analysis and functional study. The siRNA sequences were shown in Table S1 [19].

### 2.11. Cell Proliferation Assay

ECA109 and TE-1 cells in the logarithmic phase were digested with trypsin to prepare single-cell suspension, and the concentration was adjusted to 5 × 10^4^/mL. After 24 h of culture, 100 *μ*L cell suspension was inoculated into 96-well plate, negative control hole and blank hole were set up at the same time, and 4 parallel groups were set up, with 3 multiple holes and one blank hole in each group, and continued culture for 0 h, 24 h, 48 h, and 72 h. A group of cells were taken out at each time point and 10 *μ*L CCK8 reagent was added to each hole. After incubating in the cell incubator for 2 h, the absorbance (*A*) at the 450 nm wavelength was measured [33].

### 2.12. Clone Formation

Five hundred cells in the logarithmic growth phase were seeded in each well of a 6-well plate in triplicate and cultured under normal conditions for 14 days. After the cloning was completed, the cells were washed with PBS for 3 times, fixed 30-60 min with 1 mL of 4% paraformaldehyde in each well, and then stained with 0.1% crystal violet, and the colonies were counted by microscope [34].

### 2.13. Wound Healing Assay

We use a sterile ruler and the tip of the skin marker to draw a straight width line on the cell-covered plate. The cells were washed with PBS and cultured in serum-free medium. After 48 hours, we observe the width of the line using a microscope. Three random views were taken and quantified under the microscope [34].

### 2.14. Detection of Glutamine and Glutamate

ECA109 and TE-1 cells in the logarithmic phase were inoculated into six-well plates, respectively. Cells and culture supernatant were collected for detection the next day. Cell lysates were detected using a glutamine kit (Solarbio, Beijing, China). The protein concentration was determined by the BCA method, and the glutamine content of unit protein was determined by the BCA method. Culture supernatant and cell lysate were collected, and glutamate detection kit (Nanjing Jiacheng Institute of Biological Engineering, Nanjing, China) was used for glutamate detection. The content of glutamate was calculated using the measured value of cell-free control medium as the initial concentration. These experiments were repeated at least three times.

### 2.15. Statistical Analysis

SPSS19.0 statistical software and GraphPad Prism 8.0 software (GraphPad, San Diego CA, USA) were used for statistical analysis and image drawing. The measurement data were expressed as mean ± standard deviation (*X* ± *S*). *T*-test was used for pairwise comparison, and analysis of variance was used for multigroup comparison. *P* < 0.05 was considered to indicate a statistically significant difference.

## 3. Results

### 3.1. Gene Expression and Clinical Diagnostic Efficacy of YY1

**Figure 1:**
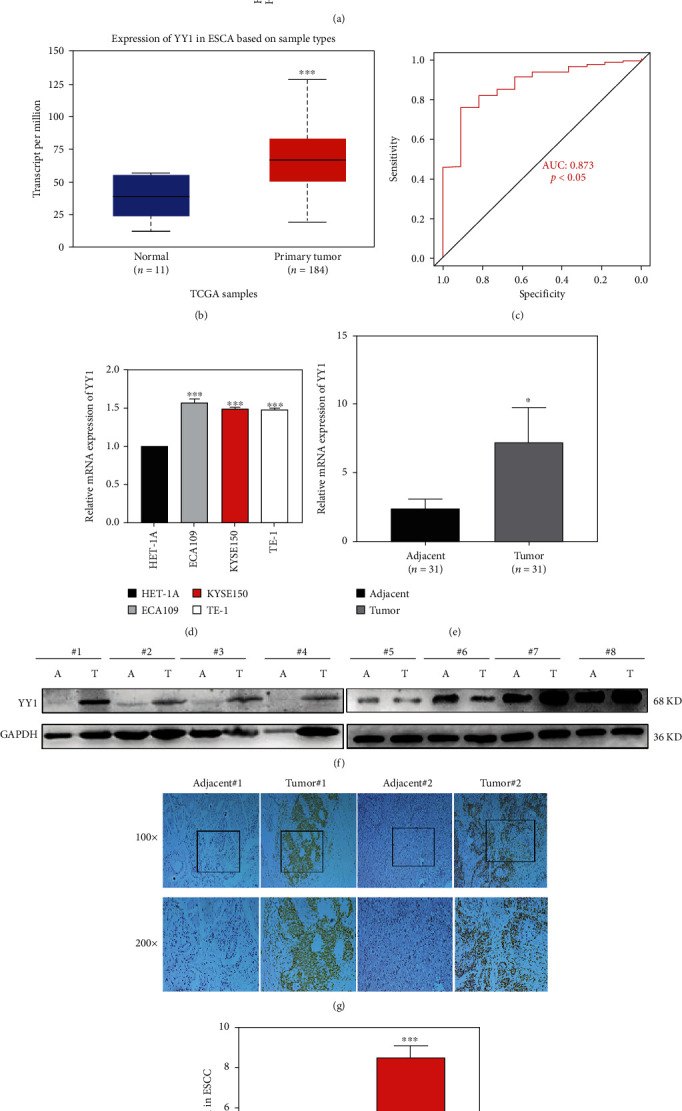
The expression of YY1 in ESCA and its relationship with prognosis of ESCA patients. (a) Expression of YY1 in different tumors. (b) Expression of YY1 in ESCA based on sample types. (c) The diagnostic value of YY1 in ESCA patients was demonstrated by ROC curve. (d) The mRNA expression of YY1 in ESCC cell line and normal esophageal epithelial cell line. (e) The mRNA expression of YY1 in 31 pairs of ESCC cancer tissues and matched adjacent tissues. (f) The protein expression of YY1 in ESCA cancer tissues and matched adjacent tissues. (g) Representative IHC images of YY1 staining in ESCC tumor or adjacent tissues (scale bar, 100 *μ*m; magnification, 100x and 200x). (h) IHC scores of YY1 in ESCA cancer tissues and matched adjacent tissues. ^∗^*P* < 0.05, ^∗∗^*P* < 0.01, and ^∗∗∗^*P* < 0.001. ROC: receiver operating characteristic; AUC: area under curve; A: adjacent tissue; T: tumor tissue.

Firstly, the expression of YY1 mRNA in 31 tumors was analyzed by TIMER. The results showed that the expression level of YY1 was significantly increased in 14 tumors, including ESCA, gastric cancer, and liver cancer (Figure 1(a)). Secondly, the mRNA expression levels of YY1 in ESCA and adjacent normal tissues were statistically analyzed by online analysis of UALCAN. The results showed that the expression level of YY1 in ESCA tissues was significantly higher than that in normal tissues (*P* < 0.001) (Figure 1(b)). In order to evaluate the diagnostic value of YY1, we used the expression data of TCGA-ESCA to draw the receiver operating characteristic (ROC) curve to predict the survival rates of ESCA patients. The results showed that the area under the ROC curve of YY1 was 0.873 (Figures 1(c), *P* < 0.05). It demonstrated that YY1 had good diagnostic efficacy for ESCA patients.

### 3.2. Association of mRNA Expression of YY1 with Clinicopathological Features of ESCA Patients

**Figure 2:**
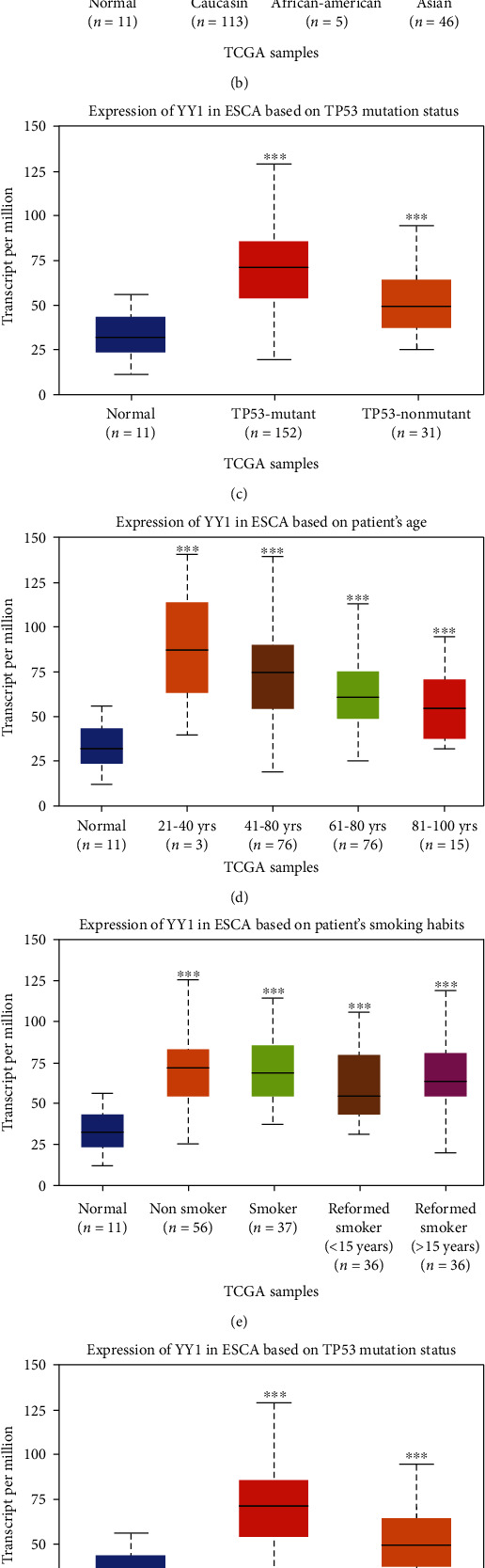
Analysis results of expression of YY1 in ESCA subgroups. (a–g) Box plot shows the mRNA expression of YY1 in normal individuals or in ESCA individual cancer stages, patient's race, nodal metastasis status, patient's age, patient's smoking habits, TP53 mutation status, and tumor histology. ^∗^*P* < 0.05, ^∗∗^*P* < 0.01, and ^∗∗∗^*P* < 0.001; ns: no significance.

**Figure 3:**
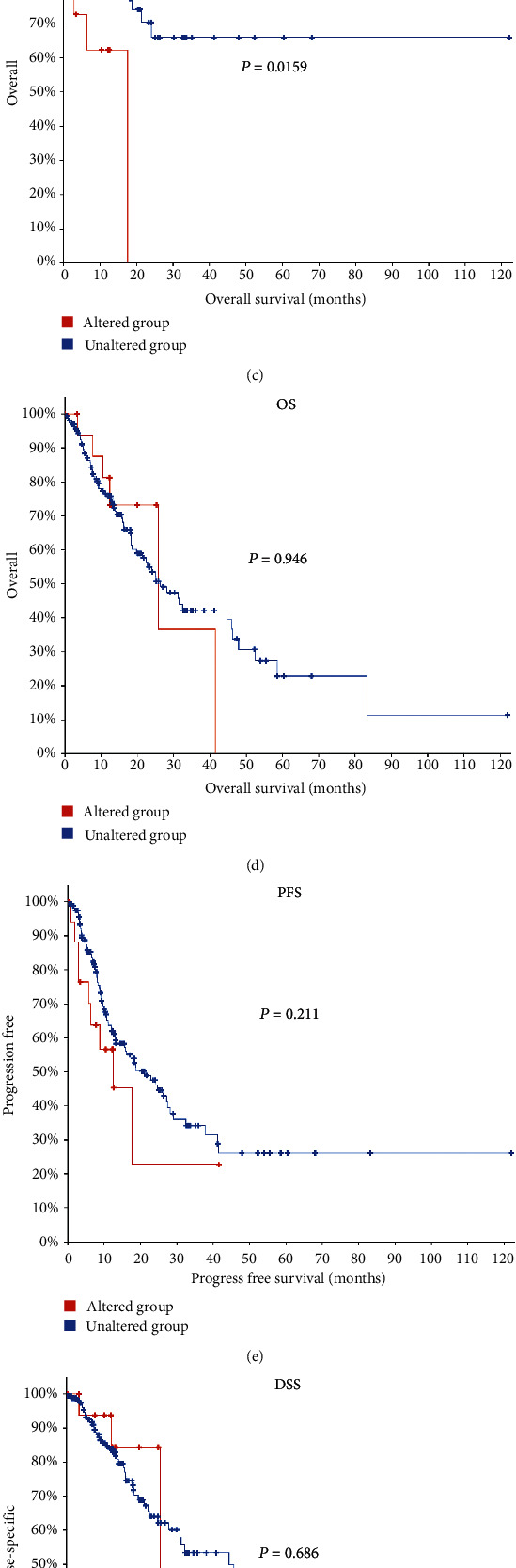
Genetic alteration and prognosis of YY1 in ESCA (cBioPortal). (a) The oncoPrint of YY1 alterations in ESCA. (b) The mutation sites of YY1 in ESCA. (c–f) Survival analyses of genetic alteration of YY1 in ESCA.

The relationship between YY1 mRNA expression and clinicopathological parameters in patients with ESCA was analyzed by UALCAN, based on individual cancer stages, patient's race, nodal metastasis status, patient's age, patient's smoking habits, TP53 mutation status, and tumor histology. As shown in Figure 2, the expression level of YY1 mRNA in ESCA patients was significantly higher than that in the normal control group (*P* < 0.05). mRNA expressions of YY1 were remarkably correlated with cancer stages. Compared to normal tissues, the mRNA expression of YY1 was significantly higher in stage 1, stage 2, stage 3, and stage 4 (Figure 2(a)). In different races, the expression of YY1 in ESCA tissues was higher than that in normal paracancerous tissues (Figure 2(b)). Then, we analyzed the relationship mRNA expression of YY1 with nodal metastasis status of ESCA patients. As shown in Figure 3(c), mRNA expressions of YY1 were significantly related to nodal metastasis status compared with normal tissues. In addition, we also found that the mRNA expression level of YY1 in ESCA tissues was significantly higher than that in normal paracancerous tissues in different ages, smoking habits, TP53 mutations, and tumor histology (Figures 2(d)–2(g)).

### 3.3. Verification of YY1 Expression in ESCA

The expression of YY1 in normal esophageal epithelial cells HET-1A and ESCC cell lines ECA109 and TE-1 was detected by real-time PCR. The results showed that the expression level of YY1 in ESCC cell lines ECA109, TE-1, and KYSE150 was significantly upregulated compared with HET-1A (*P* < 0.05) (Figure 1(d)).

To further verify the mRNA expression of YY1 in ESCA, the expression of YY1 in 31 pairs of ESCA tissues and adjacent tissues was detected by real-time PCR and immunohistochemical staining. It was found that the expression levels of YY1 mRNA (Figure 1(e)) and protein (Figures 1(g) and 1(h)) in ESCA tissues were significantly higher than those in adjacent tissues. The protein expression of YY1 in 8 pairs of ESCA cancer tissues and adjacent tissues was detected by western blot. It was found that the expression level of YY1 protein in ESCA tissues was significantly higher than that in paracancerous tissues (Figure 1(f)). Based on the above results, we found that YY1 is abnormally high expression in ESCA, suggesting that YY1 may play an important role in the occurrence and development of ESCA.

### 3.4. YY1 Gene Alteration of ESCA

We used the cBioPortal tool to determine the type and frequency of YY1 alteration in ESCA based on the DNA sequencing data of ESCA patients. We found that 10% of the ESCA cases had YY1 gene mutation (Figure 3(a)), of which 7.4% had mRNA upregulation, 1.6% had missense mutation, 5% had deletion mutation, and 0.5% had deep deletion mutation.

In addition, there was a significant correlation between YY1 gene mutation and disease-free survival (DFS) in ESCA patients (Figure 3(c)). The DFS of samples with YY1 gene alterations was significantly lower than that of samples without YY1 gene alterations (*P* < 0.05). However, there was no significant correlation between YY1 gene mutation and disease-specific survival (DSS), overall survival (OS), and progress-free survival (PFS) in patients with ESCA (Figures 3(d)–3(f), *P* < 0.05). The above results suggest that the prognosis of ESCA patients with YY1 gene mutation was worse.

### 3.5. YY1 and Immune Infiltration

**Figure 4:**
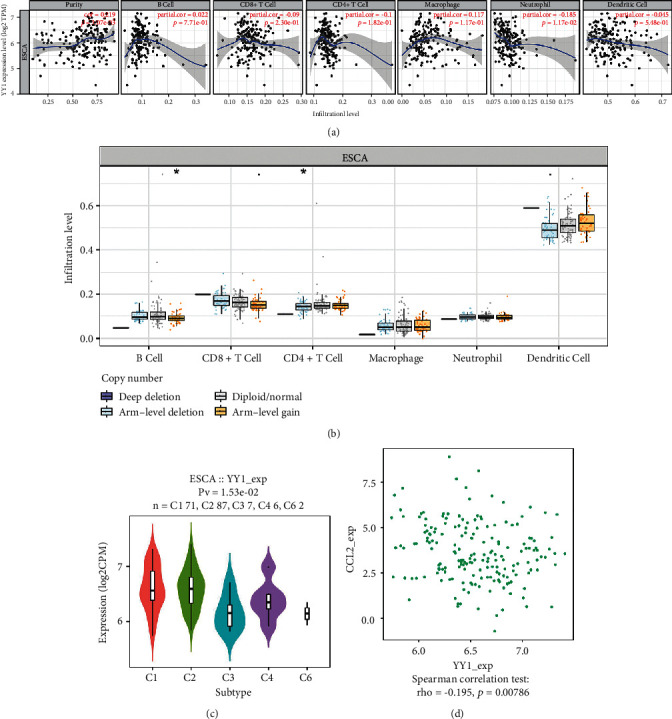
The association of YY1 with immune infiltration in ESCA. (a) YY1 expression is significantly associated with tumor purity and infiltrating of immune cells. (b) YY1 CNV affects the infiltrating of immune cells. (c) The association between YY1 expression and immune subtype using TISIDB: C1: wound healing; C2: IFN-gamma dominant; C3: inflammatory; C4: lymphocyte depleted; C5: immunologically quiet; C6 (TGF-b dominant. (d) The association between YY1 and chemokine. ^∗^*P* < 0.05.

In order to deepen the understanding of YY1 and immune gene crosstalk, we studied whether the expression level of YY1 was related to immune infiltration in ESCA by TIMER. The results showed that the expression of YY1 was significantly correlated with tumor purity (*P* = 2.90*E* − 05) and neutrophil (*P* = 1.27*E* − 02) in Figure 4(a). In order to further study the correlation between YY1 expression and immune recognition cells, we analyzed the correlation between YY1 and immune markers through the GEPIA2 database. As shown in Table 1, a variety of immune cells including T cell (general), B cell, CD8+ T cell, T cell exhaustion, Th17, Tfh, Th1, neutrophils, natural killer cell, Th2, and Mast cells were detected. The results showed that the expression of GATA3, STAT6, IL13, BCL6, and STAT3 were positively correlated with the expression of YY1 in ESCA, while the expression of CD8A, CD8B, CD2, CD3E, CD19, CD79A, KIR2DL1, KIR2DL4, CCR7, T-bet, STAT4, IL17A, PD-1, CTLA4, TPSB2, TPSAB1, MS4A2, and HDC were negatively correlated with the expression of YY1. At the same time, Th17 markers (STAT3), natural killer cell markers (KIR3DL1), and Th2 markers (STAT6 and STAT5A) were positively correlated with the expression of YY1 in normal tissues of patients with ESCA. The CNV level of YY1 also affected the infiltration level of B cells and CD4+ T cells in ESCA, and there was a significant correlation between them (Figure 4(b), *P* < 0.05). YY1 expression was significant associated with immune subtypes. Figure S1 shows the association between YY1 and ESCA-related chemokines; YY1 was negatively correlated with the ESCA-related chemokines including CCL2, CCL5, CCL11, CCL14, CCL15, CCL17, CCL19, CCL20, CCL22, CCL25, CCL28, CXCL1, CXCL2, CXCL3, CXCL6, CXCL9, CXCL11, CXCL12, CXCL13, CXCL16, and CXCL17 (*P* < 0.05). Moreover, YY1 was positively correlated with the ESCA-related chemokines including CCL7, CCL26, CXCL14, and XCL1 (Figure S1, *P* < 0.05).

### 3.6. YY1 Coexpression Networks in ESCA

**Figure 5:**
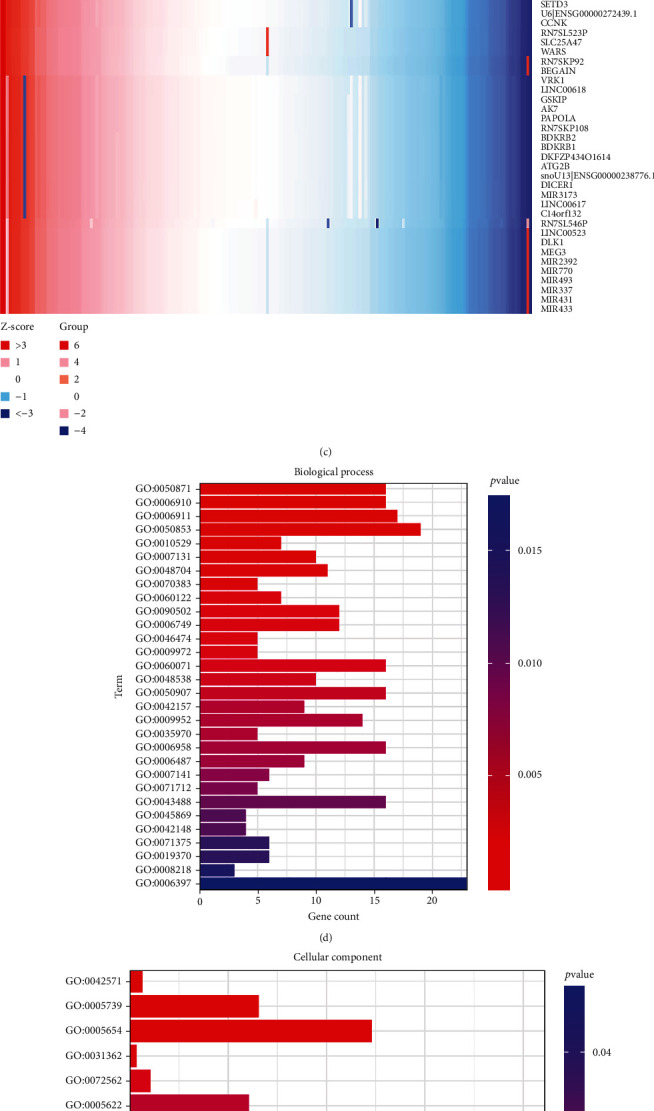
YY1 co-expression genes in ESCA (LinkedOmics). (a) The global YY1 highly correlated genes identified by spearman test in TCGA-ESCA cohort. Red indicates positively correlated genes and green indicates negatively correlated genes. (b, c): Heat maps showing top 50 genes positively and negatively correlated with YY1 in ESCA. (d) Top 22 GO terms according to biological processes. (e) The GO terms according to cellular components according to *P* value. (f) The GO terms according to molecular functions. (g) The KEGG pathway terms according to *P* value. The size of each node indicates the gene number in the corresponding pathway, whereas the *P* value is shown by the color. The redder the color, that is the more significant. GO: Gene Ontology; KEGG: Kyoto Encyclopedia of Genes and Genomes.

In order to understand the biological significance of YY1 in ESCA, we analyzed the genes related to YY1 in TCGA-ESCA data by LinkedOmics. As shown by Figure 5(a), a total of 2075 genes are represented by dark red dots, which indicate a significant positive correlation with YY1 and green represents a negative correlation. A total description of the coexpressed genes was detailed in Table S2. The first 50 significant gene sets were displayed by heat map to determine the correlation between these genes and YY1 (Figures 5(b) and 5(c)). The results showed that YY1 had an extensive effect on transcriptome. DAVID analysis showed that these genes were mainly involved in 22 biological processes (Figure 5(d)), such as glutathione metabolic process (GO: 0006749), lipoprotein metabolic process (GO: 0042157), and glycerophospholipid biosynthetic process (GO: 0046474). There were mainly located in 15 cellular components (Figure 5(e)), such as mitochondrion (GO: 0005739), nucleoplasm (GO: 0005654), and intracellular (GO: 0005622). There were mainly involved in the regulation of 21 molecular functions (Figure 5(f)), such as gamma-glutamyl transferase activity (GO: 0003840), glutathione hydrolase activity (GO: 0036374), and cytidine deaminase activity (GO: 0004126). KEGG pathway enrichment analysis showed that these genes were mainly enriched in nine pathways (Figure 5(g)), such as pathways in cancer (hsa05200), glutathione metabolism (hsa00480), and glycosphingolipid biosynthesis-globo series (hsa00603). A total description of the functional enrichment analysis of YY1-related genes was detailed in Table S3. The above results suggest that YY1 plays an important role in the glutamine metabolism.

### 3.7. YY1 Promotes the Proliferation and the Migration of ECA109 and TE-1 Cells

**Figure 6:**
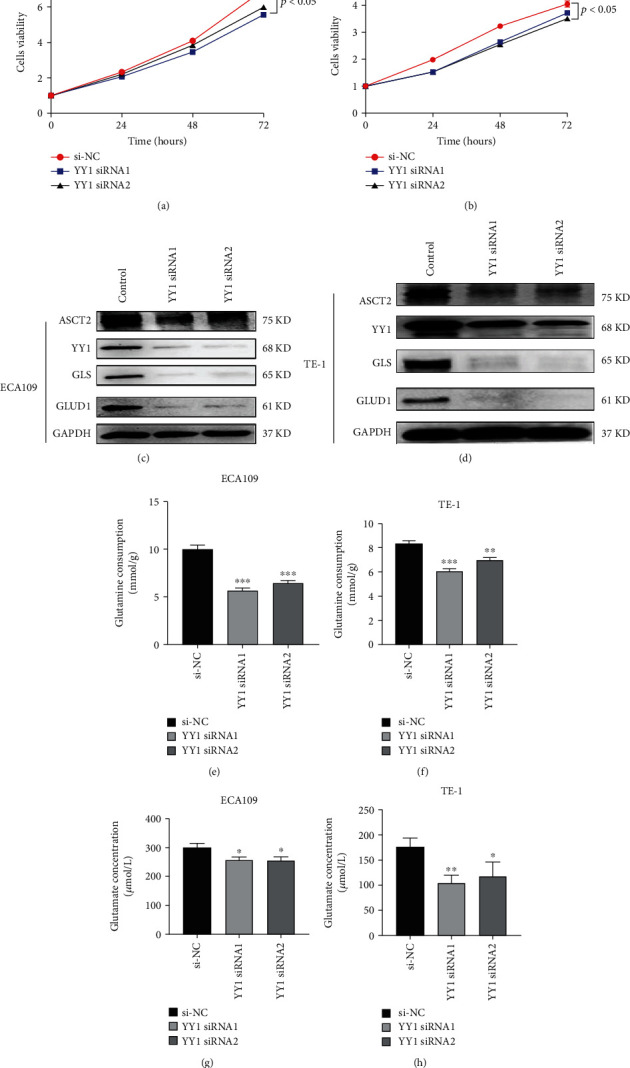
Silencing YY1 inhibited proliferation and glutamine metabolism of ECA109 and TE-1 cells. (a, b): Multiplication assays of ECA109 and TE-1 cells after siRNA knockdown of YY1. NC: negative control. Data are shown as the mean ± SD of three independent experiments. (c, d) The protein expressions of ASCT2, GLS, and GLUD1 in ECA109 and TE-1 cells after siRNA knockdown of YY1. (e, f) Analysis of the concentration of glutamate in ECA109 cells and TE-1 cells after siRNA knockdown of YY1. (g, h) Analysis of the consumption of glutamine in ECA109 cells and TE-1 cells after siRNA knockdown of YY1. ^∗^*P* < 0.05, ^∗∗^*P* < 0.01, and ^∗∗∗^*P* < 0.001; *n* = 3.

**Figure 7:**
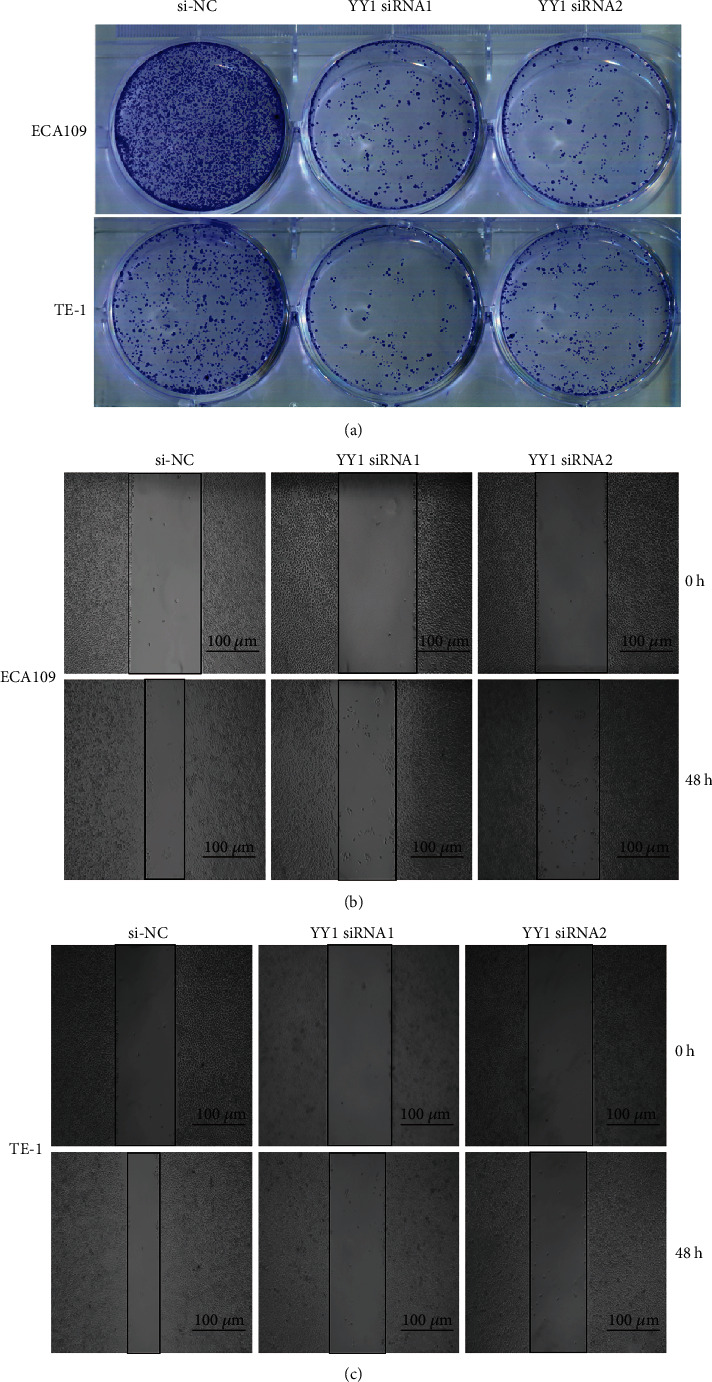
Silencing YY1 inhibited proliferation and migration of of ECA109 and TE-1 cells. (a) Determination of proliferation ability of ECA109 and TE-1 cells by clone formation. (b, c) Determination of the migratory ability of ECA109 and TE-1 cells by wound-healing assay. Scale bar = 100 *μ*m.

In order to explore the effect of YY1 on the proliferation and the migration of ESCC cell line, we knocked down YY1 in ECA109 and TE-1 to detect its effect on the proliferation of ESCC cell lines. After siRNA-YY1-1 and siRNA-YY1-2 were transfected into ECA109 and TE-1, the results of western blot showed that YY1 protein in ECA109 and TE-1 cells decreased significantly, indicating that YY1 knockdown was successful and could be used in follow-up experiment (Figures 6(c) and 6(d)). CCK8 assay and clone formation showed that cell proliferation was inhibited in the YY1 knockdown group (Figures 6(a), 6(b), and 7(a)). Wound healing assay showed that cell migration was inhibited in the YY1 knockdown group (Figures 7(b) and 7(c)).

### 3.8. YY1 Participates in the Regulation of Glutamine Metabolism

Through the previous functional analysis, we found that YY1 may be involved in glutamine metabolism process. So, we knocked down YY1 in ECA109 and TE-1 to detect its effect on glutamine metabolism. Downexpression of YY1 expression significantly decreased the amount of glutamine uptake and glutamate production ECA109 and TE-1 (Figures 6(e)–6(h), *P* < 0.05). After siRNA knockdown of YY1 in ECA109 and TE-1, the protein expression levels of ASCT2, GLS, and GLUD1were significantly downregulated (Figures 6(c) and 6(d)). These results suggest that YY1 may promote the occurrence of glutamine metabolism in ESCA cells and then participate in the tumorigenesis of ESCA.

## 4. Discussion

The incidence of ESCA ranks third among malignant tumors of digestive system and seventh among all common cancers [1]. Because the onset of ESCA is hidden, the tumor is usually diagnosed at an advanced stage. Despite the intensive multimode therapy of surgery, radiotherapy, and chemotherapy, the prognosis of advanced ESCA is still poor, and its 5-year survival rate is low, which is due to its late diagnosis and rapid metastasis [35]. Thus, it is of high importance to identify more specific and sensitive biomarkers and understand the pathogenetic mechanisms of ESCA and is very helpful for clinicians to choose suitable treatments for ESCA patients. It is estimated that more than 7% of vertebrate genes contain YY1 binding sites, indicating that YY1 has a wide range of regulatory effects on a number of basic biological processes that includes cell cycle [36], cell development [37], and cell proliferation [38]. In addition to its regulatory role in normal biological processes, YY1 may also have the potential to act as an initiator of tumorigenesis. More and more evidence shows that YY1 transcription factor has become an important factor affecting the development and progression of cancer. The activation or inactivation of YY1 transcription factor will lead to uncontrolled expression of multiple gene sets, thus promoting cancer development, cell survival, and proliferation and inducing tumor angiogenesis [39]. Moreover, it can be used as a tumor marker for diagnosis and prognosis at the same time [13]. However, there are relatively few studies on the role of YY1 in the development of ESCA. Therefore, this paper firstly analyzes the relationship between its expression and pathological features, in the hope that it can be used as a potential biomarker to promote the future evaluation of treatment results of patients with ESCA. To the best of our knowledge, our study was the first clarifying the systematic analysis of YY1 genes in ESCA patients using multiple bioinformatics databases. The results showed that mRNA expression of YY1 in ESCA tissues was significantly higher than in normal tissues. Furthermore, we investigated the association between the clinicopathological data and the expression of YY1 of ESCA patients. The mRNA expression of YY1 was remarkably correlated with all clinicopathological parameters in ESCA tissues.

To address the expression and function of YY1 in ESCA, we further detected the expression of YY1 in ESCA tissue. Because of the high morbidity rate of ESCC in China, we selected ESCC cell lines (ECA109 and TE1) for further study. As a tumor suppressor gene, the potential of YY1 as a valuable biomarker for the development of cancer had become increasingly known. So far, a great deal of evidence shows that YY1 played a key role in regulating the proliferation and development of tumor cells. The expression of YY1 was upregulated in many cancers, which might play a regulatory role in the activation, progression, and/or maintenance of malignant tumors in many tumor models and was associated with poor prognosis [40], including breast cancer, pancreatic cancer, prostate cancer [41], and colon cancer [42]. YY1 could promote the cell proliferation of breast cancer cells by inhibiting the expression of a cell cycle inhibitor [43]. YY1 could suppress the invasion and proliferation of pancreatic cancer cells through MUC4/ErbB2/p38/mef2cMEF2C-dependent mechanism [44]. YY1 can also inhibit the proliferation of pancreatic ductal adenocarcinoma cells by downregulating the expression of its potential target genes [45, 46]. YY1 directly banded to the target promoter region of protooncogene c-Myc on laryngeal carcinoma cells and inhibited its promoter activity to promote tumor cell proliferation and migration [47]. The expression of YY1 was increased in endometrioid endometrial carcinoma (EESCA). But the proliferation and migration of EESCA cells in vitro and in vivo were significantly inhibited after siRNA knockdown of YY1. And the overexpression of YY1 promoted the proliferation and growth of EESCA cells [48]. These results indicated that YY1 played a universal regulatory role in many biological processes, including proliferation, metabolism, and development. In order to verify the expression of YY1 in ESCA, we detected the expression of YY1 in ESCA tissue and its normal paracancerous tissues, ESCC cell lines, and esophageal normal epithelial cell line. We found that the YY1 expression was upregulated in ESCA tissue and ESCC cell lines. In addition, the proliferation and the migration of ESCC cells were significantly inhibited after YY1 knockdown, which indicated that YY1 could promote the growth and proliferation of ESCC. These research findings revealed YY1 played an oncogenic role in initiation and progression of ESCC.

Various studies had also shown that YY1 could regulate the expression of immune cells and participate in tumor cell immune escape. Kosasih and Bonavida [49] had shown that YY1 is a necessary transcription factor in the maturation and proliferation of immune cell *α β* lineage. YY1 could regulate the expression of Th2 and other immune cells during the differentiation of T cell subsets. Because YY1 binding sites often exist in the promoters of genes expressed in germinal centers, YY1 was also one of the main genes regulating the development of germinal center B cells [50]. Hays and Bonavida [51] found that there are several signal crosstalk pathways between YY1 and the expression regulation of immune cells and regulate the drug resistance of tumor cells to cellular immunotherapy through these pathways. In this study, we found that YY1 is significantly related to the abundance of immune cells and significantly related to immune gene markers and chemokines. These findings strongly suggest that YY1 plays an important role in the immune activity of the tumor microenvironment.

In order to further explore the molecular function of YY1 in ESCA, we analyzed the effects of YY1 gene on biological process by the DAVID approach, and the results suggest that glutamine metabolism may promote ESCC progression by influencing pathways in cancer. Meanwhile, we demonstrated that the amount of glutamine uptake and glutamate production were significantly decreased in YY1-downregulted ESCC cells. Glutamine metabolism was another important way of energy metabolism in tumor cells in addition to Warburg effect [52]. In addition to consuming glucose and energy supply, tumor cells consumed and utilized glutamine in large quantities, which provided a source of macromolecular substances for maintaining cell biosynthesis, energy metabolism, and cell homeostasis, thus driving tumor growth and proliferation [53, 54]. ASCT2 can transport extracellular glutamine into cells for biosynthesis [55] or exported back out of the cell by antiporters in exchange for other amino acids [56]. GLS could catalyze glutamine to glutamate, which was the first metabolic enzyme of glutamine and the key rate-limiting enzyme. Tumor cells increased the metabolism of glutamine by adding GLS, thus providing energy for the growth of tumor cells [57]. GLUD1 converted glutamate into alpha-ketoglutarate in mitochondria, which provided energy for tricarboxylic acid cycle and participates in glutamine metabolism [58]. Some studies had shown that the degree of malignancy of the tumor is positively correlated with the expression of glutamine metabolism [59]. Furthermore, we found that ASCT2, GLS, and GLUD1 showed a downward trend in the knockdown of YY1 expression cells. This research shows that the downregulation of YY1 expression may mediate the regulation of glutamine metabolism. These results indicated that YY1 may mediate glutamine metabolism to regulate the development of ESCA.

## 5. Conclusion

We found that the expression of YY1 was generally increased in tumors and participated in a variety of biological processes of ESCA. YY1 promoted the proliferation of ESCC cells and might regulate glutamine metabolism through ASCT2, GLS, and GLUD1, thus affecting the growth of ESCC cells. Therefore, this suggested that the YY1 transcription factor might be a diagnostic marker and therapeutic target of ESCA.

## Figures and Tables

**Table 1:** The association of YY1 expression and immune markers in GEPIA2.

Description	Gene markers	ESCA					
		Tumor		Normal		GTEX	
		*R*	*P*	*R*	*P*	*R*	*P*
CD8+ T cell	CD8A	-0.11	0.15	0.16	0.6	0.15	0.00019
	CD8B	-0.17	0.018	0.0096	0.98	0.15	9.80*E* − 05
T cell (general)	CD2	-0.2	0.0069	0.12	0.7	0.17	8.90*E* − 06
	CD3E	-0.24	0.0013	0.13	0.67	0.16	2.80*E* − 05
B cell	CD19	-0.14	0.053	0.092	0.77	0.088	0.024
	CD79A	-0.25	0.00051	-0.17	0.58	0.04	0.31
Natural killer cell	KIR2DL1	-0.057	0.44	0.51	0.075	-0.1	0.01
	KIR2DL3	-0.049	0.51	0.28	0.35	-0.073	0.063
	KIR2DL4	-0.065	0.38	-0.038	0.9	0.078	0.046
	KIR3DL1	-0.083	0.27	0.57	0.043	-0.074	0.058
	KIR3DL2	-0.0073	0.92	0.0028	0.99	0.023	0.55
	KIR3DL3	-0.018	0.81	0.15	0.64	0.035	0.38
	KIR2DS4	-0.033	0.66	0.19	0.53	-0.067	0.085
Neutrophils	CD66b	0.022	0.76	0.18	0.57	-0.069	0.076
	CD11b	0.084	0.26	0.48	0.099	0.008	0.84
	CCR7	-0.23	0.0019	0.21	0.49	0.091	0.02
Th1	T-bet	-0.11	0.16	0.15	0.62	0.083	0.034
	STAT4	-0.066	0.37	0.49	0.092	-0.12	0.0019
	TNF-*α*(TNF)	0.13	0.07	0.25	0.4	0.058	0.14
Th2	GATA3	0.23	0.0015	0.0.53	0.064	−8.20*E* − 05	1
	STAT6	0.13	0.075	0.65	0.016	0.4	0
	STAT5A	-0.011	0.88	0.84	0.00034	0.057	0.15
	IL13	0.043	0.56	0.31	0.3	-0.12	0.0024
Tfh	BCL6	0.36	4.70*E* − 07	0.55	0.052	0.19	8.30*E* − 07
Th17	STAT3	0.3	4.90*E* − 05	0.79	0.0012	0.32	0
	IL17A	-0.0084	0.91	0.26	0.39	0.11	0.0036
T cell exhaustion	PD-1 (PDCD1)	-0.15	0.047	0.2	0.52	0.15	7.90*E* − 05
	CTLA4	-0.027	0.72	0.21	0.49	0.13	0.00068
	LAG3	-0.048	0.52	0.33	0.27	-0.07	0.074
	TIM-3	0.032	0.67	0.52	0.07	0.013	0.74
Mast cells	TPSB2	-0.18	0.013	0.4	0.17	-0.12	0.0016
	TPSAB1	-0.22	0.0034	0.44	0.14	-0.1	0.0077
	CPA3	-0.085	0.25	0.49	0.091	-0.048	0.22
	MS4A2	-0.051	0.49	0.29	0.34	-0.097	0.013
	HDC	-0.1	0.18	-0.32	0.29	-0.2	0.0019

## Data Availability

The data used to support the findings of this study are available from the corresponding author upon request.

## Abbreviations

ASCT2: Alanine-, serine-, cysteine-preferring transporter 2
CNA: Copy number alterations
DFS: Disease-free survival
DSS: Disease-specific survival
EAC: Esophageal adenocarcinoma
ESCA: Esophageal carcinoma
ESCC: Esophageal squamous cell carcinoma
G6PD: Glucose-6-phosphatedehydrogenase
GLS: Glutaminase
GLUD1: Glutamate dehydrogenase
ROC: Receive operating characteristic
AUC: Area under curve
OS: Overall survival
PFS: Progress-free survival
PGC-1*β*: Peroxisome proliferator-activated receptor gamma coactivator-1 *β*
YY1: Yin-Yang-1.

## Supplementary Materials

Supplementary 1 Figure S1: the association between YY1 and ESCA-related chemokines.

Supplementary 2 Table S1: primers and oligonucleotides sequences.

Supplementary 3 Table S2: YY1 coexpressed genes.

Supplementary 4 Table S3: functional enrichment analysis of YY1-related genes. The table consists of four parts, which are composed of biological processes (BP), cellular components (CC), molecular functions (MF), and Kyoto Encyclopedia of Genes and Genomes (KEGG). We screened out the items with statistical significance according to *p* < 0.01. It includes 22 biological processes, 15 cellular components, 21 molecular functions, and 9 KEGG signaling pathways. These items are used to draw bubble charts.
