# Supplementary figures and images for: Upregulation of Yin-Yang-1 Associates with Proliferation and Glutamine Metabolism in Esophageal Carcinoma
Source: Int J Genomics. 2022 Mar 20;2022:9305081. doi: 10.1155/2022/9305081 (PMC8961439; doi:10.1155/2022/9305081)

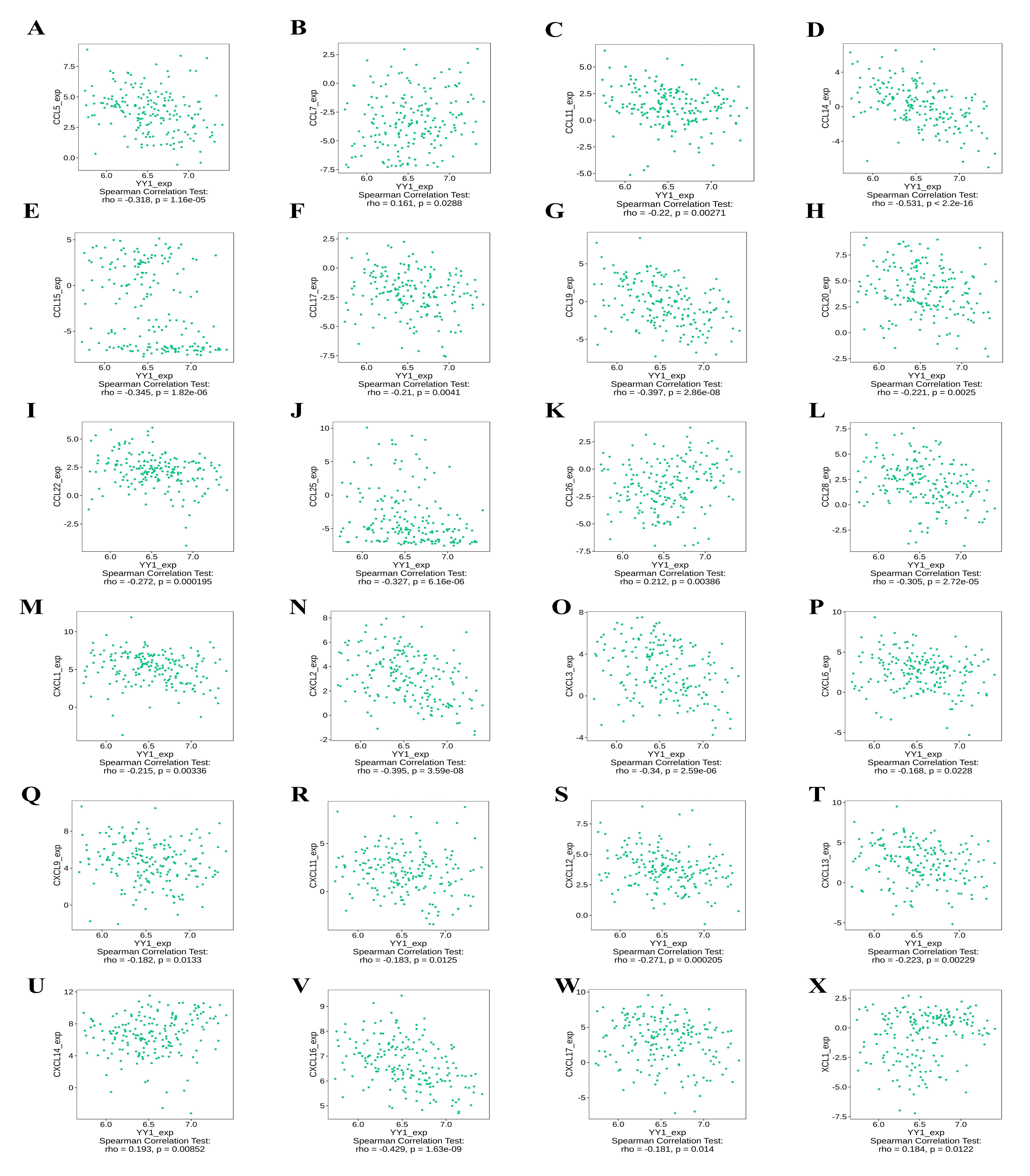

Supplement: Supplementary 1 — Figure S1: the association between YY1 and ESCA-related chemokines. [file 9305081.f1.jpg]
