# Supplementary material for: Upregulation of Yin-Yang-1 Associates with Proliferation and Glutamine Metabolism in Esophageal Carcinoma
Source: Int J Genomics. 2022 Mar 20;2022:9305081. doi: 10.1155/2022/9305081 (PMC8961439; doi:10.1155/2022/9305081)
Supplement: Supplementary 2 — Table S1: primers and oligonucleotides sequences. [file 9305081.f2.pdf]

Table S1 Primers and oligonuceotides sequences

	**Oilgonucleotides**
**PCR primers**	
PCR-YY1-F	GCGGAGCCCTCAGCCATGGCCTCG
PCR-YY1-R	CAGCGGCTGCAGAGCGATCATGG
PCR-GAPDH-F	GGAGCGAGATCCCTCCAAAAT
PCR-GAPDH-R	GGCTGTTGTCATACTTCTCATGG
**siRNAs**	
Human YY1 siRNA1	CGACGACTACATTGAACAA
Human YY1 siRNA2	CCTGAAATCTCACATCTTA

Note: F：Forward Primer；R：Reverse primer
