# Supplementary material for: Upregulation of Yin-Yang-1 Associates with Proliferation and Glutamine Metabolism in Esophageal Carcinoma
Source: Int J Genomics. 2022 Mar 20;2022:9305081. doi: 10.1155/2022/9305081 (PMC8961439; doi:10.1155/2022/9305081)
Supplement: Supplementary 3 — Table S2: YY1 coexpressed genes. [file 9305081.f3.pdf]

Query	Statistic	P-value	FDR (BH)	Event_SD	Event_TD
TLK2	0.243524337	0.000865017	0.00999145	179	182
TPD52	0.243581748	0.000862504	0.00996707	179	181
PSKH2	0.243626173	0.000860564	0.00994929	179	182
ZIM2	-0.243632162	0.000860303	0.00994929	179	183
ARID3A	0.243674451	0.00085846	0.00993424	179	183
CFD	0.243674451	0.00085846	0.00993424	179	183
ELANE	0.243674451	0.00085846	0.00993424	179	183
KISS1R	0.243674451	0.00085846	0.00993424	179	183
MED16	0.243674451	0.00085846	0.00993424	179	183
PRTN3	0.243674451	0.00085846	0.00993424	179	183
R3HDM4	0.243674451	0.00085846	0.00993424	179	183
WDR18	0.243674451	0.00085846	0.00993424	179	183
SLC25A39	0.243837891	0.000851373	0.00988918	179	181
ZMAT1	0.243856355	0.000850576	0.00988455	179	175
TCEAL2	0.243856355	0.000850576	0.00988455	179	175
TCEAL6	0.243856355	0.000850576	0.00988455	179	175
BEX5	0.243856355	0.000850576	0.00988455	179	175
TCP11X3P	0.243856355	0.000850576	0.00988455	179	175
MIR1284	-0.243908254	0.000848338	0.00988172	179	181
PHOSPHO1	0.243941203	0.000846921	0.00986985	179	181
ZNF274	-0.244005292	0.00084417	0.00984242	179	183
RN7SKP32	0.244006972	0.000844098	0.00984242	179	180
ZNF329	-0.244048637	0.000842314	0.00983003	179	183
GXYLT2	-0.244158936	0.000837608	0.00977972	179	181
ZNF784	-0.244170601	0.000837111	0.00977854	179	183
ZNF580	-0.244170601	0.000837111	0.00977854	179	183
ZNF581	-0.244170601	0.000837111	0.00977854	179	183
CCDC106	-0.244170601	0.000837111	0.00977854	179	183
EIF4E3	-0.244306062	0.000831368	0.0097298	179	181
GPR27	-0.244306062	0.000831368	0.0097298	179	181
PROK2	-0.244306062	0.000831368	0.0097298	179	181
RN7SL271F	-0.244306062	0.000831368	0.0097298	179	181
LINC00877	-0.244306062	0.000831368	0.0097298	179	181
LINC0087C	-0.244306062	0.000831368	0.0097298	179	181
ABCA7	0.244423832	0.000826405	0.0096992	179	183
HMHA1	0.244423832	0.000826405	0.0096992	179	183
POLR2E	0.244423832	0.000826405	0.0096992	179	183
GPX4	0.244423832	0.000826405	0.0096992	179	183
RYBP	-0.244473662	0.000824313	0.00969301	179	181
RNA5SP517	0.244565817	0.000820457	0.00965225	179	175
CXorf51A	0.244565817	0.000820457	0.00965225	179	175
CXorf51B	0.244565817	0.000820457	0.00965225	179	175
HLF	0.244600037	0.000819029	0.0096492	179	181
MMD	0.244600037	0.000819029	0.0096492	179	181
SNORA68|E	0.244858192	0.000808332	0.00953224	179	181
ATP5G1	0.244858192	0.000808332	0.00953224	179	181
UBE2Z	0.244858192	0.000808332	0.00953224	179	181
STK11	0.244901586	0.000806547	0.00952479	179	183
FUT1	-0.244915523	0.000805974	0.00952257	179	183
FGF21	-0.244915523	0.000805974	0.00952257	179	183

GALP	-0.245142489	0.000796701	0.00942199	179	183
ZSCAN5B	-0.245142489	0.000796701	0.00942199	179	183
ZSCAN5C	-0.245142489	0.000796701	0.00942199	179	183
ZSCAN5D	-0.245142489	0.000796701	0.00942199	179	183
PPP4R2	-0.245155865	0.000796158	0.00942199	179	181
MIR767	0.245200368	0.000794353	0.00941669	179	175
U2AF2	-0.245242664	0.00079264	0.00940089	179	183
ZNF787	-0.245323574	0.000789374	0.00936663	179	183
GABRA3	0.245338117	0.000788788	0.00936416	179	175
ZNF652	0.245377369	0.000787209	0.0093499	179	181
RIMS2	0.245540307	0.000780685	0.00927686	179	180
snoU13|EN	-0.245622273	0.000777422	0.00924251	179	179
TMEM40	-0.245622273	0.000777422	0.00924251	179	179
CAND2	-0.245622273	0.000777422	0.00924251	179	179
RPL32	-0.245622273	0.000777422	0.00924251	179	179
SNORA7A	-0.245622273	0.000777422	0.00924251	179	179
SNORA25|E	0.245721329	0.000773495	0.00921795	179	176
PCDH19	0.245721329	0.000773495	0.00921795	179	176
FZD2	0.245813836	0.000769845	0.00918328	179	181
ZSCAN4	-0.245944968	0.000764697	0.00912627	179	183
ZNF551	-0.245944968	0.000764697	0.00912627	179	183
ZNF154	-0.245944968	0.000764697	0.00912627	179	183
ZNF135	-0.245958817	0.000764155	0.00912627	179	183
MBNL1	0.246793583	0.000732144	0.00875463	179	181
TMEM14E	0.246793583	0.000732144	0.00875463	179	181
P2RY1	0.246793583	0.000732144	0.00875463	179	181
SBNO2	0.246805861	0.000731682	0.00875463	179	183
ZNF134	-0.246949606	0.000726299	0.00870154	179	183
ZNF211	-0.246949606	0.000726299	0.00870154	179	183
ARL13A	0.247023653	0.000723541	0.00867688	179	176
TMEM35	0.247023653	0.000723541	0.00867688	179	176
TRMT2B	0.247023653	0.000723541	0.00867688	179	176
XKRX	0.247023653	0.000723541	0.00867688	179	176
NOG	0.247067334	0.000721918	0.00867422	179	181
TSEN2	-0.247134533	0.000719428	0.00864849	179	179
RNA5SP123	-0.247134533	0.000719428	0.00864849	179	179
C3orf83	-0.247134533	0.000719428	0.00864849	179	179
MKRN2	-0.247134533	0.000719428	0.00864849	179	179
RAF1	-0.247134533	0.000719428	0.00864849	179	179
TEX14	0.24757639	0.000703251	0.00847459	179	181
U3|ENSG0C	0.24757639	0.000703251	0.00847459	179	181
CNGB3	0.247848174	0.000693469	0.00836484	179	182
ZNF544	-0.247848532	0.000693456	0.00836484	179	183
ZNF8	-0.247848532	0.000693456	0.00836484	179	183
ZNF418	-0.24795039	0.000689822	0.00833302	179	183
ZNF256	-0.24795039	0.000689822	0.00833302	179	183
C19orf18	-0.24795039	0.000689822	0.00833302	179	183
ZNF606	-0.24795039	0.000689822	0.00833302	179	183
ZSCAN1	-0.24795039	0.000689822	0.00833302	179	183
U8|ENSG0C	0.24805335	0.000686167	0.00830912	179	182
TTC14	0.24805335	0.000686167	0.00830912	179	182

RN7SL229F	0.24805335	0.000686167	0.00830912	179	182
CCDC39	0.248274769	0.000678366	0.00822673	179	182
ZNF548	-0.24882126	0.000659462	0.00800139	179	183
ZNF17	-0.24882126	0.000659462	0.00800139	179	183
RN7SL526F	-0.248929021	0.000655792	0.00796466	179	183
RNA5SP136	-0.249129492	0.000649015	0.00788622	179	181
SHQ1	-0.249129492	0.000649015	0.00788622	179	181
B4GALNT2	0.249239896	0.000645311	0.0078489	179	181
KIF18B	0.249822887	0.00062607	0.00761861	179	181
PPARG	-0.250039613	0.000619053	0.00753693	179	179
RN7SKP14	0.250214318	0.000613449	0.00747238	179	181
DCAF4L2	0.250300282	0.000610709	0.00744266	179	182
ZNF530	-0.250364217	0.000608679	0.00742157	179	183
RNA5SP271	0.250485942	0.00060483	0.00737827	179	181
NACAP1	0.250488351	0.000604754	0.00737827	179	180
RN7SL563F	0.250488351	0.000604754	0.00737827	179	180
PABPC1	0.250531696	0.000603389	0.00737159	179	180
RNU6ATAC4	0.250531696	0.000603389	0.00737159	179	180
MIR5089	0.250568423	0.000602235	0.00736475	179	181
LRRC37A17	0.250568423	0.000602235	0.00736475	179	181
RPRML	0.250568423	0.000602235	0.00736475	179	181
ZC2HC1A	0.250605862	0.000601061	0.00736129	179	181
IL7	0.250605862	0.000601061	0.00736129	179	181
GNGT2	0.250625012	0.000600461	0.00736122	179	181
ABI3	0.250625012	0.000600461	0.00736122	179	181
SNX31	0.250687012	0.000598522	0.00734472	179	181
U8|ENSG00	0.250828486	0.00059412	0.00729431	179	181
MSC	0.250828486	0.00059412	0.00729431	179	181
TBC1D3P2	0.251256654	0.000580979	0.00714005	179	182
TAF7L	0.251280313	0.000580261	0.00713476	179	175
BTK	0.251280313	0.000580261	0.00713476	179	175
TIMM8A	0.251280313	0.000580261	0.00713476	179	175
RPL36A	0.251280313	0.000580261	0.00713476	179	175
GLA	0.251280313	0.000580261	0.00713476	179	175
HNRNPH2	0.251280313	0.000580261	0.00713476	179	175
ARMCX4	0.251280313	0.000580261	0.00713476	179	175
ARMCX1	0.251280313	0.000580261	0.00713476	179	175
ARMCX6	0.251280313	0.000580261	0.00713476	179	175
ARMCX3	0.251280313	0.000580261	0.00713476	179	175
ARMCX2	0.251280313	0.000580261	0.00713476	179	175
NXF5	0.251280313	0.000580261	0.00713476	179	175
MIR1203	0.251475057	0.00057438	0.00710476	179	181
ANKRD46	0.252028769	0.000557958	0.00690508	179	181
ZNF549	-0.252370602	0.000548038	0.0067857	179	183
ZNF550	-0.252370602	0.000548038	0.0067857	179	183
ZNF416	-0.252370602	0.000548038	0.0067857	179	183
ZIK1	-0.252370602	0.000548038	0.0067857	179	183
FMNL1	0.253105672	0.000527254	0.00654144	179	181
SPATA32	0.253105672	0.000527254	0.00654144	179	181
MAP3K14	0.253105672	0.000527254	0.00654144	179	181
CLTC	0.253264392	0.000522864	0.00649672	179	181

NIPAL2	0.253691629	0.000511212	0.00635513	179	181
ANGPT1	0.253699953	0.000510987	0.00635513	179	180
RNA5SP269	0.253801809	0.000508246	0.00632461	179	181
NMT1	0.253956076	0.00050412	0.00627643	179	181
TRPA1	0.254316647	0.000494598	0.00616096	179	181
ODF1	0.254535069	0.000488911	0.00609318	179	180
DCAF13	0.254721934	0.000484093	0.00603618	179	180
RN7SL405P	0.254843081	0.000480993	0.00600055	179	181
CCDC103	0.254843081	0.000480993	0.00600055	179	181
FAM187A	0.254843081	0.000480993	0.00600055	179	181
GFAP	0.254843081	0.000480993	0.00600055	179	181
EFTUD2	0.254965411	0.000477882	0.00597377	179	181
U3|ENSG00	0.255190692	0.000472201	0.00590573	179	181
SPANXN1	0.256098014	0.000449946	0.00563024	179	174
C1QL1	0.256304536	0.000445018	0.00557139	179	181
DCAKD	0.256304536	0.000445018	0.00557139	179	181
HRSP12	0.256310959	0.000444866	0.00557139	179	181
POP1	0.256310959	0.000444866	0.00557139	179	181
FBXO43	0.256783069	0.000433791	0.00544183	179	181
POLR2K	0.256783069	0.000433791	0.00544183	179	181
snoU13|EN	-0.256807892	0.000433216	0.00544012	179	181
HOXB1	0.256931237	0.000430368	0.0054071	179	181
PKIA	0.257007512	0.000428616	0.00538782	179	181
NUP210	-0.257189615	0.00042446	0.00533828	179	179
HDAC11	-0.257189615	0.00042446	0.00533828	179	179
FBLN2	-0.257189615	0.00042446	0.00533828	179	179
MRPL45P2	0.257343387	0.000420979	0.00530258	179	181
NPEPPS	0.257343387	0.000420979	0.00530258	179	181
KPNB1	0.257343387	0.000420979	0.00530258	179	181
FZD6	0.258020954	0.000405953	0.00512113	179	180
YWHAZ	0.258134365	0.000403488	0.00509262	179	180
RN7SL685P	0.258134365	0.000403488	0.00509262	179	180
CNTN3	-0.258141822	0.000403326	0.00509262	179	181
WWP1	0.258195048	0.000402174	0.00508381	179	182
RMDN1	0.258195048	0.000402174	0.00508381	179	182
CPNE3	0.258195048	0.000402174	0.00508381	179	182
RN7SKP189	0.258600652	0.000393494	0.00498172	179	174
LINC00630	0.258709212	0.000391201	0.00495521	179	175
RPL30	0.258977128	0.000385594	0.00488669	179	181
KCNB2	0.259010393	0.000384903	0.00488043	179	181
SNORA9|EN	0.25912988	0.00038243	0.00485156	179	176
TTLL6	0.259486669	0.000375135	0.00476145	179	181
CENPI	0.259546007	0.000373934	0.00474864	179	176
DRP2	0.259546007	0.000373934	0.00474864	179	176
MSI2	0.25955315	0.00037379	0.00474864	179	181
PRR11	0.261135564	0.000343065	0.00436333	179	181
SMG8	0.261135564	0.000343065	0.00436333	179	181
SLC25A32	0.261369098	0.000338734	0.00431268	179	180
GRN	0.261503534	0.000336264	0.00428343	179	181
FAM171A2	0.261503534	0.000336264	0.00428343	179	181
ITGA2B	0.261503534	0.000336264	0.00428343	179	181

GPATCH8	0.261503534	0.000336264	0.00428343	179	181
RN7SL258F	0.261503534	0.000336264	0.00428343	179	181
SPAG1	0.261585778	0.000334761	0.00427528	179	181
CTHRC1	0.261705261	0.000332589	0.00424973	179	180
NSF	0.261775163	0.000331324	0.00423575	179	181
VEZF1	0.261835455	0.000330237	0.00422403	179	181
EBLN2	-0.262206719	0.000323615	0.00414146	179	181
snoU13|EN	-0.262206719	0.000323615	0.00414146	179	181
PDZRN3	-0.262206719	0.000323615	0.00414146	179	181
ABRA	0.262372392	0.000320699	0.00411053	179	180
KLF10	0.263163573	0.000307111	0.00393839	179	180
RN7SKP85	0.263708421	0.000298066	0.00382438	179	181
PPP1R12C	-0.264133078	0.000291188	0.00373807	179	183
RN7SL437F	0.264595636	0.000283865	0.00364595	179	181
NLRP7	-0.26473628	0.000281672	0.00361967	179	183
NLRP2	-0.26473628	0.000281672	0.00361967	179	183
MIR5680	0.265126482	0.000275672	0.00354623	179	180
RRM2B	0.265250737	0.000273786	0.0035238	179	180
UBR5	0.265250737	0.000273786	0.0035238	179	180
OSR2	0.265531779	0.000269565	0.00347308	179	181
snoU13|EN	0.265624466	0.000268186	0.00345711	179	180
RNU6ATAC8	0.265624466	0.000268186	0.00345711	179	180
RGS22	0.265677864	0.000267394	0.0034505	179	181
MIR599	0.265787896	0.000265771	0.00343133	179	181
MIR875	0.265787896	0.000265771	0.00343133	179	181
SNORA72|E	0.265896836	0.000264172	0.00341425	179	181
C8orf47	0.265896836	0.000264172	0.00341425	179	181
C8orf56	0.266033961	0.000262172	0.00339195	179	180
BAALC	0.266033961	0.000262172	0.00339195	179	180
MIR3151	0.266033961	0.000262172	0.00339195	179	180
RNF19A	0.266140816	0.000260624	0.0033772	179	181
MIR4471	0.266140816	0.000260624	0.0033772	179	181
SULF1	0.266163933	0.00026029	0.00337641	179	181
RNU6ATAC3	0.266309182	0.000258201	0.00335107	179	181
GOSR2	0.266309182	0.000258201	0.00335107	179	181
FER1L6	0.266451356	0.000256172	0.00332822	179	181
STAU2	0.266849292	0.00025057	0.00325715	179	181
AZIN1	0.267064604	0.000247587	0.00322006	179	180
OXR1	0.267195344	0.000245792	0.00319839	179	180
C22orf24	0.26720944	0.000245599	0.00319756	179	181
YWHAH	0.26720944	0.000245599	0.00319756	179	181
snoU13|EN	0.26720944	0.000245599	0.00319756	179	181
RN7SL305F	0.26720944	0.000245599	0.00319756	179	181
HOXB13	0.267284928	0.000244569	0.00319086	179	181
MIR3185	0.267284928	0.000244569	0.00319086	179	181
PRAC	0.267284928	0.000244569	0.00319086	179	181
RN7SL125F	0.267284928	0.000244569	0.00319086	179	181
HOXB4	0.267487205	0.000241828	0.00316176	179	181
MIR10A	0.267487205	0.000241828	0.00316176	179	181
HOXB5	0.267487205	0.000241828	0.00316176	179	181
HOXB6	0.267487205	0.000241828	0.00316176	179	181

HOXB7	0.267487205	0.000241828	0.00316176	179	181
HOXB8	0.267487205	0.000241828	0.00316176	179	181
HOXB9	0.267487205	0.000241828	0.00316176	179	181
MIR196A1	0.267487205	0.000241828	0.00316176	179	181
RPL7	0.267662246	0.000239479	0.00314432	179	181
RDH10	0.267662246	0.000239479	0.00314432	179	181
DYNLL2	0.267726574	0.000238621	0.00313638	179	181
OR4D1	0.267726574	0.000238621	0.00313638	179	181
MATN2	0.267727901	0.000238604	0.00313638	179	181
LY96	0.26793002	0.000235927	0.00310591	179	181
GJC1	0.267968911	0.000235415	0.00310082	179	181
HIGD1B	0.267968911	0.000235415	0.00310082	179	181
ATP6V1C1	0.268366874	0.000230237	0.00303585	179	180
RN7SL350F	0.269130243	0.0002206	0.00291032	179	181
NCALD	0.269247959	0.000219148	0.00289271	179	180
snoU13|EN	0.269247959	0.000219148	0.00289271	179	180
SRSF1	0.269303382	0.000218467	0.0028868	179	181
HOXB2	0.269380902	0.000217519	0.00287579	179	181
HOXB3	0.269380902	0.000217519	0.00287579	179	181
SNORD77|E	0.270102866	0.000208865	0.00276434	179	181
MIR1273A	0.270102866	0.000208865	0.00276434	179	181
GDAP1	0.270133246	0.000208508	0.00276257	179	181
MIR301A	0.270203078	0.00020769	0.00275319	179	181
RN7SKP94	0.270372438	0.000205717	0.00272851	179	181
MRPS23	0.270372438	0.000205717	0.00272851	179	181
CUEDC1	0.270372438	0.000205717	0.00272851	179	181
DCSTAMP	0.270655219	0.000202462	0.00268966	179	180
ACBD4	0.27172285	0.000190602	0.00253345	179	181
HEXIM1	0.27172285	0.000190602	0.00253345	179	181
UBE2W	0.271883058	0.000188879	0.00251325	179	181
TCEB1	0.271883058	0.000188879	0.00251325	179	181
TMEM70	0.271883058	0.000188879	0.00251325	179	181
JPH1	0.27225521	0.000184933	0.00246471	179	181
PI15	0.272430384	0.000183102	0.00244162	179	181
TERF1	0.272669042	0.000180635	0.00241002	179	181
RN7SL449F	0.272742947	0.000179877	0.00240121	179	181
MYC	0.273022953	0.000177033	0.00236452	179	181
HEXIM2	0.273185986	0.000175397	0.00234392	179	181
PVT1	0.274021445	0.00016723	0.00223599	179	181
CDC27	0.27418752	0.00016565	0.00221606	179	181
ARHGAP27	0.274218561	0.000165356	0.00221332	179	181
LARGE	0.27426899	0.000164879	0.00220814	179	181
TATDN1	0.274715574	0.000160716	0.00215354	179	181
NDUFB9	0.274715574	0.000160716	0.00215354	179	181
RNA5SP275	0.275175811	0.000156529	0.0020997	179	180
RSPO2	0.275175811	0.000156529	0.0020997	179	180
OR4D2	0.275356323	0.000154914	0.0020803	179	181
TRMT12	0.275728879	0.000151631	0.00203732	179	181
ITGB3	0.275931786	0.00014987	0.00201475	179	181
EFCAB13	0.275931786	0.00014987	0.00201475	179	181
RAD51C	0.276347538	0.000146322	0.00196919	179	181

SBSPON	0.276388981	0.000145973	0.00196556	179	181
RN7SL716F	0.276835897	0.000142255	0.00191653	179	181
TRIM37	0.276835897	0.000142255	0.00191653	179	181
SKA2	0.276835897	0.000142255	0.00191653	179	181
MIR454	0.276835897	0.000142255	0.00191653	179	181
PPM1E	0.276893691	0.00014178	0.00191431	179	181
MTSS1	0.277436665	0.000137396	0.00185612	179	181
TMEM65	0.277509869	0.000136815	0.00184927	179	181
RNF139	0.277569589	0.000136342	0.00184389	179	181
snoU13|EN	0.278644083	0.000128096	0.00173332	179	181
RNA5SP272	0.278738075	0.000127397	0.00172481	179	182
DPYS	0.278958146	0.000125775	0.00170378	179	180
C17orf47	0.279640426	0.000120868	0.0016382	179	181
MIR4662B	0.280091198	0.000117725	0.00159648	179	181
WNT3	0.280234371	0.000116743	0.00158402	179	181
WNT9B	0.280234371	0.000116743	0.00158402	179	181
MIR548A3	0.280367679	0.000115835	0.00157343	179	180
LRP12	0.280367679	0.000115835	0.00157343	179	180
LAPTM4B	0.280404105	0.000115589	0.0015718	179	181
TMEM75	0.280971229	0.000111808	0.00152123	179	181
EIF3E	0.28157638	0.000107902	0.00146889	179	180
EMC2	0.28157638	0.000107902	0.00146889	179	180
TMEM74	0.28157638	0.000107902	0.00146889	179	180
SLC5A1	0.282639833	0.000101345	0.00138191	179	181
AP1B1P1	0.282639833	0.000101345	0.00138191	179	181
C22orf42	0.282639833	0.000101345	0.00138191	179	181
SQLE	0.283072317	9.88E-05	0.00134925	179	181
KIAA0196	0.283072317	9.88E-05	0.00134925	179	181
CALCOCO2	0.28327733	9.76E-05	0.00133445	179	181
PLCD3	0.283329689	9.73E-05	0.00133106	179	181
ARL17A	0.283795544	9.46E-05	0.00129552	179	181
ARL17B	0.283795544	9.46E-05	0.00129552	179	181
CRHR1	0.283795544	9.46E-05	0.00129552	179	181
FAM215B	0.283795544	9.46E-05	0.00129552	179	181
KANSL1	0.283795544	9.46E-05	0.00129552	179	181
LRRC37A2	0.283795544	9.46E-05	0.00129552	179	181
LRRC37A4F	0.283795544	9.46E-05	0.00129552	179	181
LRRC37A	0.283795544	9.46E-05	0.00129552	179	181
MAPT	0.283795544	9.46E-05	0.00129552	179	181
PLEKHM1	0.283795544	9.46E-05	0.00129552	179	181
RN7SL199F	0.283795544	9.46E-05	0.00129552	179	181
RN7SL656F	0.283795544	9.46E-05	0.00129552	179	181
RN7SL730F	0.283795544	9.46E-05	0.00129552	179	181
RN7SL739F	0.283795544	9.46E-05	0.00129552	179	181
SPPL2C	0.283795544	9.46E-05	0.00129552	179	181
STH	0.283795544	9.46E-05	0.00129552	179	181
SUPT4H1	0.284760343	8.94E-05	0.00123423	179	181
RNF43	0.284760343	8.94E-05	0.00123423	179	181
CRISPLD1	0.284916537	8.85E-05	0.00122417	179	181
PCAT2	0.284918512	8.85E-05	0.00122417	179	181
MYL4	0.284958292	8.83E-05	0.00122249	179	181

snoU13|EN	0.284958292	8.83E-05	0.00122249	179	181
ZFHX4	0.285044163	8.79E-05	0.00121761	179	181
PEX2	0.285044163	8.79E-05	0.00121761	179	181
ZNF572	0.285144198	8.73E-05	0.00121173	179	181
C17orf104	0.285306912	8.65E-05	0.0012007	179	181
CCDC43	0.285306912	8.65E-05	0.0012007	179	181
DBF4B	0.285306912	8.65E-05	0.0012007	179	181
RN7SL819P	0.285306912	8.65E-05	0.0012007	179	181
ADAM11	0.285306912	8.65E-05	0.0012007	179	181
HSF5	0.285580053	8.51E-05	0.00118459	179	181
MTMR4	0.285580053	8.51E-05	0.00118459	179	181
SEPT4	0.285580053	8.51E-05	0.00118459	179	181
MMP16	0.285788935	8.41E-05	0.00117188	179	182
ZFPM2	0.28603889	8.28E-05	0.00115514	179	180
LINC00964	0.286386202	8.11E-05	0.00113201	179	181
TSPYL5	0.287410638	7.63E-05	0.0010651	179	181
U3|ENSG00	0.287410638	7.63E-05	0.0010651	179	181
PCAT1	0.287662781	7.51E-05	0.00105025	179	181
snoU13|EN	0.288432346	7.17E-05	0.0010032	179	180
NUDCD1	0.288544282	7.12E-05	0.00099701	179	180
ENY2	0.288544282	7.12E-05	0.00099701	179	180
WDYHV1	0.288577374	7.11E-05	0.00099614	179	181
EPX	0.289374218	6.77E-05	0.00094981	179	181
MKS1	0.289374218	6.77E-05	0.00094981	179	181
MIR142	0.289693047	6.64E-05	0.00093268	179	181
MIR4736	0.289693047	6.64E-05	0.00093268	179	181
LPO	0.289848127	6.58E-05	0.00092499	179	181
MPO	0.289848127	6.58E-05	0.00092499	179	181
BZRAP1	0.289848127	6.58E-05	0.00092499	179	181
PKHD1L1	0.290261696	6.42E-05	0.00090359	179	180
EBAG9	0.290261696	6.42E-05	0.00090359	179	180
SNORD112|	0.290261696	6.42E-05	0.00090359	179	180
TRHR	0.290549698	6.31E-05	0.00088942	179	180
FBXO32	0.290802402	6.21E-05	0.00087634	179	181
POM121L9P	0.291737684	5.87E-05	0.0008282	179	183
CASC9	0.292267793	5.68E-05	0.00080223	179	181
HNF4G	0.292267793	5.68E-05	0.00080223	179	181
SPECC1L	0.292418677	5.63E-05	0.00079576	179	183
TRPS1	0.292779119	5.50E-05	0.00077881	179	181
RN7SKP155	0.293197918	5.36E-05	0.00075947	179	181
KLHL38	0.293197918	5.36E-05	0.00075947	179	181
ANXA13	0.293197918	5.36E-05	0.00075947	179	181
FAM91A1	0.293197918	5.36E-05	0.00075947	179	181
RNA5SP443	0.293911659	5.13E-05	0.00072851	179	181
MIR1205	0.294659413	4.90E-05	0.00069606	179	181
OSGIN2	0.296230709	4.45E-05	0.00063185	179	181
FLJ27365	0.296329135	4.42E-05	0.00062835	179	183
MIR3619	0.297864501	4.02E-05	0.00057133	179	183
DECR1	0.297881655	4.01E-05	0.00057104	179	181
CALB1	0.29848477	3.86E-05	0.0005502	179	181
GRAMD4	0.299087642	3.72E-05	0.00053009	179	183

MTDH	0.299655797	3.59E-05	0.00051178	179	181
RN7SL20P	0.299748841	3.57E-05	0.00050909	179	181
TRIB1	0.300010981	3.51E-05	0.00050104	179	181
RN7SL590F	0.300010981	3.51E-05	0.00050104	179	181
DEPDC5	0.300031064	3.51E-05	0.00050098	179	181
MIR1207	0.30031522	3.44E-05	0.00049237	179	181
ATAD2	0.300318053	3.44E-05	0.00049237	179	181
MIR548AA1	0.300318053	3.44E-05	0.00049237	179	181
TAF2	0.300574605	3.39E-05	0.00048522	179	181
DSCC1	0.300574605	3.39E-05	0.00048522	179	181
RN7SL396F	0.300574605	3.39E-05	0.00048522	179	181
RNA5SP277	0.300574605	3.39E-05	0.00048522	179	181
NSMCE2	0.300900035	3.32E-05	0.00047644	179	181
LINC00536	0.30158179	3.18E-05	0.00045659	179	181
MIR2053	0.302052711	3.09E-05	0.00044341	179	180
RN7SL329F	0.30247152	3.00E-05	0.00043201	179	181
snoU13|EN	0.302856851	2.93E-05	0.00042179	179	180
RIPK2	0.303018511	2.90E-05	0.00041772	179	182
COX6C	0.303553343	2.80E-05	0.00040394	179	181
CCDC26	0.303835394	2.75E-05	0.00039696	179	181
MRPL13	0.304244113	2.68E-05	0.00038695	179	181
DEPTOR	0.304384114	2.66E-05	0.00038372	179	181
NFAM1	0.304522357	2.64E-05	0.00038056	179	182
POLDIP3	0.304522357	2.64E-05	0.00038056	179	182
RN7SKP80	0.304522357	2.64E-05	0.00038056	179	182
RRP7A	0.304522357	2.64E-05	0.00038056	179	182
RRP7B	0.304522357	2.64E-05	0.00038056	179	182
SERHL2	0.304522357	2.64E-05	0.00038056	179	182
SERHL	0.304522357	2.64E-05	0.00038056	179	182
RN7SKP206	0.305205091	2.52E-05	0.00036574	179	181
COLEC10	0.305218257	2.52E-05	0.00036565	179	181
COL14A1	0.305607061	2.46E-05	0.00035683	179	181
RNA5SP276	0.305709161	2.44E-05	0.00035471	179	181
CABIN1	0.306253338	2.36E-05	0.00034269	179	183
SYBU	0.306258783	2.36E-05	0.00034269	179	180
TTC28	0.306365739	2.34E-05	0.00034062	179	182
EIF3H	0.306750396	2.28E-05	0.00033246	179	181
CELSR1	0.3069746	2.25E-05	0.00032787	179	183
ADRBK2	0.307281089	2.21E-05	0.00032162	179	183
RNA5SP494	0.307281089	2.21E-05	0.00032162	179	183
ENPP2	0.307384524	2.19E-05	0.00031986	179	181
PPARA	0.307484142	2.18E-05	0.00031799	179	183
MYO18B	0.307520781	2.17E-05	0.00031742	179	183
RN7SKP169	0.307520781	2.17E-05	0.00031742	179	183
NBN	0.3078615	2.13E-05	0.00031085	179	181
LINC00977	0.30793967	2.11E-05	0.00030947	179	181
UTP23	0.308296354	2.07E-05	0.00030256	179	181
RAD21	0.308885841	1.99E-05	0.00029136	179	181
MIR3610	0.308885841	1.99E-05	0.00029136	179	181
AARD	0.308885841	1.99E-05	0.00029136	179	181
KCNV1	0.309017433	1.97E-05	0.00028939	179	180

DERL1	0.309745326	1.88E-05	0.00027614	179	181
RNY4P5	0.309745326	1.88E-05	0.00027614	179	181
TBC1D31	0.309745326	1.88E-05	0.00027614	179	181
U3|ENSG0C	0.309745326	1.88E-05	0.00027614	179	181
FBXO7	0.310173557	1.83E-05	0.00026916	179	181
TNFRSF11E	0.310278017	1.82E-05	0.00026748	179	181
EIF4ENIF1	0.310315483	1.81E-05	0.00026699	179	181
TBC1D22A	0.310449304	1.80E-05	0.00026482	179	183
SLC2A11	0.310534016	1.79E-05	0.00026351	179	183
RN7SL268F	0.310534016	1.79E-05	0.00026351	179	183
MIF	0.310534016	1.79E-05	0.00026351	179	183
RFPL2	0.310775419	1.76E-05	0.00025984	179	181
SLC5A4	0.310775419	1.76E-05	0.00025984	179	181
TTLL1	0.311012221	1.73E-05	0.00025614	179	183
BIK	0.311012221	1.73E-05	0.00025614	179	183
MCAT	0.311012221	1.73E-05	0.00025614	179	183
TSPO	0.311012221	1.73E-05	0.00025614	179	183
TTLL12	0.311012221	1.73E-05	0.00025614	179	183
SCUBE1	0.311012221	1.73E-05	0.00025614	179	183
RFPL3	0.311039491	1.73E-05	0.00025614	179	181
RFPL3S	0.311039491	1.73E-05	0.00025614	179	181
RTCB	0.311039491	1.73E-05	0.00025614	179	181
BPIFC	0.311039491	1.73E-05	0.00025614	179	181
MTERFD1	0.311142201	1.72E-05	0.00025549	179	181
UQCRB	0.311142201	1.72E-05	0.00025549	179	181
PTDSS1	0.311173988	1.71E-05	0.00025527	179	181
CPQ	0.311274675	1.70E-05	0.00025374	179	181
DRG1	0.31161345	1.66E-05	0.0002483	179	182
RN7SKP153	0.31164867	1.66E-05	0.00024788	179	181
GDF6	0.311813561	1.64E-05	0.00024535	179	181
LINC00861	0.31184224	1.64E-05	0.00024504	179	181
FAM84B	0.312149986	1.61E-05	0.00024027	179	181
SUSD2	0.31279646	1.54E-05	0.00023038	179	183
GGT5	0.31279646	1.54E-05	0.00023038	179	183
COL22A1	0.312811088	1.54E-05	0.00023038	179	182
C22orf43	0.312893895	1.53E-05	0.00022932	179	183
GUSBP11	0.312893895	1.53E-05	0.00022932	179	183
IGLL1	0.312893895	1.53E-05	0.00022932	179	183
RGL4	0.312893895	1.53E-05	0.00022932	179	183
VPREB3	0.312893895	1.53E-05	0.00022932	179	183
ZNF70	0.312893895	1.53E-05	0.00022932	179	183
C22orf15	0.312893895	1.53E-05	0.00022932	179	183
CHCHD10	0.312893895	1.53E-05	0.00022932	179	183
MMP11	0.312893895	1.53E-05	0.00022932	179	183
SMARCB1	0.312893895	1.53E-05	0.00022932	179	183
DERL3	0.312893895	1.53E-05	0.00022932	179	183
SAMD12	0.312972125	1.52E-05	0.00022932	179	181
MAL2	0.313049333	1.51E-05	0.00022864	179	181
ADORA2A	0.313178856	1.50E-05	0.00022683	179	183
MTBP	0.313856988	1.43E-05	0.00021701	179	181
SNTB1	0.313856988	1.43E-05	0.00021701	179	181

CHEK2	0.314141618	1.41E-05	0.00021321	179	182
SNORA76|E	0.314322165	1.39E-05	0.0002108	179	181
BCR	0.31446201	1.38E-05	0.00020898	179	183
CES5AP1	0.31446201	1.38E-05	0.00020898	179	183
GGTLC2	0.31446201	1.38E-05	0.00020898	179	183
GNAZ	0.31446201	1.38E-05	0.00020898	179	183
IGLC1	0.31446201	1.38E-05	0.00020898	179	183
IGLC2	0.31446201	1.38E-05	0.00020898	179	183
IGLC3	0.31446201	1.38E-05	0.00020898	179	183
IGLC7	0.31446201	1.38E-05	0.00020898	179	183
IGLJ1	0.31446201	1.38E-05	0.00020898	179	183
IGLJ2	0.31446201	1.38E-05	0.00020898	179	183
IGLJ3	0.31446201	1.38E-05	0.00020898	179	183
IGLJ4	0.31446201	1.38E-05	0.00020898	179	183
IGLJ5	0.31446201	1.38E-05	0.00020898	179	183
IGLJ6	0.31446201	1.38E-05	0.00020898	179	183
IGLJ7	0.31446201	1.38E-05	0.00020898	179	183
IGLL5	0.31446201	1.38E-05	0.00020898	179	183
MIR650	0.31446201	1.38E-05	0.00020898	179	183
POM121L1F	0.31446201	1.38E-05	0.00020898	179	183
PRAMEF24F	0.31446201	1.38E-05	0.00020898	179	183
PRAME	0.31446201	1.38E-05	0.00020898	179	183
RAB36	0.31446201	1.38E-05	0.00020898	179	183
RN7SL263F	0.31446201	1.38E-05	0.00020898	179	183
RTDR1	0.31446201	1.38E-05	0.00020898	179	183
TOP3B	0.31446201	1.38E-05	0.00020898	179	183
VPREB1	0.31446201	1.38E-05	0.00020898	179	183
ZDHHC8P1	0.31446201	1.38E-05	0.00020898	179	183
ZNF280A	0.31446201	1.38E-05	0.00020898	179	183
ZNF280B	0.31446201	1.38E-05	0.00020898	179	183
snoU13|EN	0.31446201	1.38E-05	0.00020898	179	183
MIR1208	0.314617602	1.36E-05	0.00020898	179	181
TFIP11	0.314891453	1.34E-05	0.0002069	179	183
TPST2	0.314891453	1.34E-05	0.0002069	179	183
MIR548J	0.314891453	1.34E-05	0.0002069	179	183
CRYBB1	0.314891453	1.34E-05	0.0002069	179	183
CRYBA4	0.314891453	1.34E-05	0.0002069	179	183
SNORA32|E	0.314891809	1.34E-05	0.0002069	179	181
NOV	0.314891809	1.34E-05	0.0002069	179	181
MIAT	0.315175601	1.31E-05	0.00020391	179	183
PITPNB	0.315241608	1.31E-05	0.00020315	179	183
MICAL3	0.315364899	1.30E-05	0.00020161	179	183
SEZ6L	0.315982624	1.25E-05	0.00019359	179	183
RNA5SP495	0.315982624	1.25E-05	0.00019359	179	183
ASPHD2	0.315982624	1.25E-05	0.00019359	179	183
HPS4	0.315982624	1.25E-05	0.00019359	179	183
SRRD	0.315982624	1.25E-05	0.00019359	179	183
SFI1	0.316110048	1.23E-05	0.00019256	179	181
PISD	0.316110048	1.23E-05	0.00019256	179	181
PRR14L	0.316110048	1.23E-05	0.00019256	179	181
C8orf37	0.316875724	1.17E-05	0.00018329	179	181

CSMD3	0.317028804	1.16E-05	0.00018153	179	180
ZHX2	0.317215057	1.15E-05	0.00017939	179	181
FAM83A	0.317274776	1.14E-05	0.00017879	179	181
MIR4663	0.317274776	1.14E-05	0.00017879	179	181
C8orf76	0.317274776	1.14E-05	0.00017879	179	181
ZHX1	0.317274776	1.14E-05	0.00017879	179	181
snoU13|EN	0.317274776	1.14E-05	0.00017879	179	181
SYN3	0.317443293	1.13E-05	0.00017734	179	181
TOP1MT	0.317617069	1.12E-05	0.00017539	179	182
RHPN1	0.317617069	1.12E-05	0.00017539	179	182
BID	0.317796051	1.10E-05	0.00017352	179	183
LINC0052ε	0.317796051	1.10E-05	0.00017352	179	183
PLEKHF2	0.31780472	1.10E-05	0.00017352	179	181
C22orf31	0.317923655	1.09E-05	0.00017236	179	182
MIR4764	0.318754867	1.03E-05	0.00016309	179	181
snoU13|EN	0.318910971	1.02E-05	0.00016148	179	181
GATSL3	0.319028288	1.02E-05	0.00016031	179	182
TBC1D10A	0.319028288	1.02E-05	0.00016031	179	182
SF3A1	0.319028288	1.02E-05	0.00016031	179	182
CCDC157	0.319028288	1.02E-05	0.00016031	179	182
RNF215	0.319028288	1.02E-05	0.00016031	179	182
SEC14L2	0.319028288	1.02E-05	0.00016031	179	182
KIAA1658	0.319028288	1.02E-05	0.00016031	179	182
MTFP1	0.319028288	1.02E-05	0.00016031	179	182
SEC14L3	0.319028288	1.02E-05	0.00016031	179	182
SEC14L4	0.319028288	1.02E-05	0.00016031	179	182
OSBP2	0.319040656	1.01E-05	0.00016031	179	182
MORC2	0.319040656	1.01E-05	0.00016031	179	182
PACSIN2	0.319070495	1.01E-05	0.00016031	179	183
CERK	0.319081685	1.01E-05	0.00016031	179	183
HSCB	0.31925532	1.00E-05	0.00015929	179	182
RNA5SP497	0.319280456	9.98E-06	0.00015912	179	181
RNU12	0.319419488	9.89E-06	0.00015774	179	182
KREMEN1	0.319640104	9.74E-06	0.0001555	179	182
SDC2	0.320146485	9.41E-06	0.00015036	179	181
ZNRF3	0.320160741	9.40E-06	0.00015031	179	182
SNORA25|E	0.320182568	9.39E-06	0.00015018	179	182
NDUFAF6	0.320216453	9.37E-06	0.00014994	179	181
snoU13|EN	0.320216453	9.37E-06	0.00014994	179	181
TP53INP1	0.320216453	9.37E-06	0.00014994	179	181
MIR3150B	0.320216453	9.37E-06	0.00014994	179	181
DPY19L4	0.320260915	9.34E-06	0.00014987	179	181
MPPED1	0.320768166	9.02E-06	0.00014489	179	183
SMDT1	0.321109781	8.82E-06	0.00014166	179	182
CYP2D6	0.321109781	8.82E-06	0.00014166	179	182
CYP2D7P1	0.321109781	8.82E-06	0.00014166	179	182
TCF20	0.321109781	8.82E-06	0.00014166	179	182
SCRIB	0.32118899	8.77E-06	0.00014127	179	183
MIR937	0.32118899	8.77E-06	0.00014127	179	183
PUF60	0.32118899	8.77E-06	0.00014127	179	183
NRBP2	0.32118899	8.77E-06	0.00014127	179	183

CDPF1	0.321263913	8.72E-06	0.00014091	179	183
MAPK1	0.321268093	8.72E-06	0.00014091	179	183
RNA5SP493	0.321268093	8.72E-06	0.00014091	179	183
MN1	0.321304712	8.70E-06	0.0001408	179	183
RN7SL162F	0.321338907	8.68E-06	0.00014056	179	182
CRYBB3	0.321882624	8.36E-06	0.00013554	179	183
CRYBB2	0.321882624	8.36E-06	0.00013554	179	183
CRYBB2P1	0.321882624	8.36E-06	0.00013554	179	183
LRP5L	0.321882624	8.36E-06	0.00013554	179	183
MIR4763	0.322242541	8.16E-06	0.0001326	179	183
MIRLET7A3	0.322242541	8.16E-06	0.0001326	179	183
MIRLET7B	0.322242541	8.16E-06	0.0001326	179	183
RN7SL228F	0.322300521	8.13E-06	0.00013234	179	181
HORMAD2	0.322585617	7.97E-06	0.00012987	179	182
MIR3686	0.322761839	7.88E-06	0.0001284	179	181
VPS13B	0.322945563	7.78E-06	0.00012688	179	181
RN7SKP226	0.323130634	7.68E-06	0.00012536	179	181
NECAB1	0.323477101	7.50E-06	0.0001225	179	181
TMEM55A	0.323477101	7.50E-06	0.0001225	179	181
OTUD6B	0.323477101	7.50E-06	0.0001225	179	181
RN7SL777F	0.323477101	7.50E-06	0.0001225	179	181
LRRC69	0.323574385	7.45E-06	0.00012201	179	181
MIR4661	0.323574385	7.45E-06	0.00012201	179	181
SLC26A7	0.323574385	7.45E-06	0.00012201	179	181
UPB1	0.323721247	7.38E-06	0.00012102	179	183
DDTL	0.323792681	7.34E-06	0.00012051	179	183
DDT	0.323792681	7.34E-06	0.00012051	179	183
GSTT1	0.323792681	7.34E-06	0.00012051	179	183
GSTT2B	0.323792681	7.34E-06	0.00012051	179	183
GSTT2	0.323792681	7.34E-06	0.00012051	179	183
GSTTP2	0.323792681	7.34E-06	0.00012051	179	183
TUBA8	0.32382111	7.33E-06	0.00012051	179	183
GUCD1	0.324475445	7.00E-06	0.00011552	179	183
SNRPD3	0.324475445	7.00E-06	0.00011552	179	183
FAM211B	0.324475445	7.00E-06	0.00011552	179	183
GGT1	0.324475445	7.00E-06	0.00011552	179	183
PIWIL3	0.324475445	7.00E-06	0.00011552	179	183
SGSM1	0.324475445	7.00E-06	0.00011552	179	183
SNORD56|E	0.324475445	7.00E-06	0.00011552	179	183
TMEM211	0.324475445	7.00E-06	0.00011552	179	183
ASAP1	0.325052736	6.73E-06	0.00011161	179	181
PPM1F	0.325077611	6.72E-06	0.0001115	179	183
AIFM3	0.325099597	6.71E-06	0.0001114	179	183
ARVCF	0.325099597	6.71E-06	0.0001114	179	183
BCRP2	0.325099597	6.71E-06	0.0001114	179	183
C22orf29	0.325099597	6.71E-06	0.0001114	179	183
C22orf39	0.325099597	6.71E-06	0.0001114	179	183
CCDC116	0.325099597	6.71E-06	0.0001114	179	183
CDC45	0.325099597	6.71E-06	0.0001114	179	183
CLDN5	0.325099597	6.71E-06	0.0001114	179	183
CLTCL1	0.325099597	6.71E-06	0.0001114	179	183

COMT	0.325099597	6.71E-06	0.0001114	179	183
CRKL	0.325099597	6.71E-06	0.0001114	179	183
DGCR14	0.325099597	6.71E-06	0.0001114	179	183
DGCR2	0.325099597	6.71E-06	0.0001114	179	183
DGCR5	0.325099597	6.71E-06	0.0001114	179	183
DGCR6L	0.325099597	6.71E-06	0.0001114	179	183
DGCR6	0.325099597	6.71E-06	0.0001114	179	183
DGCR8	0.325099597	6.71E-06	0.0001114	179	183
FAM230A	0.325099597	6.71E-06	0.0001114	179	183
FAM230B	0.325099597	6.71E-06	0.0001114	179	183
FAM230C	0.325099597	6.71E-06	0.0001114	179	183
GGT2	0.325099597	6.71E-06	0.0001114	179	183
GGT3P	0.325099597	6.71E-06	0.0001114	179	183
GGTLC3	0.325099597	6.71E-06	0.0001114	179	183
GNB1L	0.325099597	6.71E-06	0.0001114	179	183
GP1BB	0.325099597	6.71E-06	0.0001114	179	183
GSC2	0.325099597	6.71E-06	0.0001114	179	183
HIC2	0.325099597	6.71E-06	0.0001114	179	183
HIRA	0.325099597	6.71E-06	0.0001114	179	183
KLHL22	0.325099597	6.71E-06	0.0001114	179	183
LZTR1	0.325099597	6.71E-06	0.0001114	179	183
MED15	0.325099597	6.71E-06	0.0001114	179	183
MIR1286	0.325099597	6.71E-06	0.0001114	179	183
MIR1306	0.325099597	6.71E-06	0.0001114	179	183
MIR130B	0.325099597	6.71E-06	0.0001114	179	183
MIR185	0.325099597	6.71E-06	0.0001114	179	183
MIR301B	0.325099597	6.71E-06	0.0001114	179	183
MIR3618	0.325099597	6.71E-06	0.0001114	179	183
MIR4761	0.325099597	6.71E-06	0.0001114	179	183
MIR649	0.325099597	6.71E-06	0.0001114	179	183
MRPL40	0.325099597	6.71E-06	0.0001114	179	183
P2RX6P	0.325099597	6.71E-06	0.0001114	179	183
P2RX6	0.325099597	6.71E-06	0.0001114	179	183
PI4KAP1	0.325099597	6.71E-06	0.0001114	179	183
PI4KAP2	0.325099597	6.71E-06	0.0001114	179	183
PI4KA	0.325099597	6.71E-06	0.0001114	179	183
POM121L4F	0.325099597	6.71E-06	0.0001114	179	183
POM121L7	0.325099597	6.71E-06	0.0001114	179	183
PPIL2	0.325099597	6.71E-06	0.0001114	179	183
PRODH	0.325099597	6.71E-06	0.0001114	179	183
RANBP1	0.325099597	6.71E-06	0.0001114	179	183
RIMBP3B	0.325099597	6.71E-06	0.0001114	179	183
RIMBP3C	0.325099597	6.71E-06	0.0001114	179	183
RIMBP3	0.325099597	6.71E-06	0.0001114	179	183
RN7SKP131	0.325099597	6.71E-06	0.0001114	179	183
RN7SKP221	0.325099597	6.71E-06	0.0001114	179	183
RN7SKP63	0.325099597	6.71E-06	0.0001114	179	183
RN7SL168F	0.325099597	6.71E-06	0.0001114	179	183
RN7SL280F	0.325099597	6.71E-06	0.0001114	179	183
RN7SL389F	0.325099597	6.71E-06	0.0001114	179	183
RN7SL812F	0.325099597	6.71E-06	0.0001114	179	183

RTN4R	0.325099597	6.71E-06	0.0001114	179	183
SCARF2	0.325099597	6.71E-06	0.0001114	179	183
SCARNA17|	0.325099597	6.71E-06	0.0001114	179	183
SCARNA17|	0.325099597	6.71E-06	0.0001114	179	183
SCARNA17|	0.325099597	6.71E-06	0.0001114	179	183
SCARNA18|	0.325099597	6.71E-06	0.0001114	179	183
SCARNA18|	0.325099597	6.71E-06	0.0001114	179	183
SCARNA18|	0.325099597	6.71E-06	0.0001114	179	183
SDF2L1	0.325099597	6.71E-06	0.0001114	179	183
SEPT5	0.325099597	6.71E-06	0.0001114	179	183
SERPIND1	0.325099597	6.71E-06	0.0001114	179	183
SLC25A1	0.325099597	6.71E-06	0.0001114	179	183
SLC7A4	0.325099597	6.71E-06	0.0001114	179	183
SMPD4P1	0.325099597	6.71E-06	0.0001114	179	183
SNAP29	0.325099597	6.71E-06	0.0001114	179	183
SNORA15|E	0.325099597	6.71E-06	0.0001114	179	183
SNORA77|E	0.325099597	6.71E-06	0.0001114	179	183
TANGO2	0.325099597	6.71E-06	0.0001114	179	183
TBX1	0.325099597	6.71E-06	0.0001114	179	183
THAP7	0.325099597	6.71E-06	0.0001114	179	183
TMEM191A	0.325099597	6.71E-06	0.0001114	179	183
TMEM191C	0.325099597	6.71E-06	0.0001114	179	183
TRMT2A	0.325099597	6.71E-06	0.0001114	179	183
TSSK2	0.325099597	6.71E-06	0.0001114	179	183
TUBA3FP	0.325099597	6.71E-06	0.0001114	179	183
TXNRD2	0.325099597	6.71E-06	0.0001114	179	183
UBE2L3	0.325099597	6.71E-06	0.0001114	179	183
UFD1L	0.325099597	6.71E-06	0.0001114	179	183
USP18	0.325099597	6.71E-06	0.0001114	179	183
USP41	0.325099597	6.71E-06	0.0001114	179	183
YDJC	0.325099597	6.71E-06	0.0001114	179	183
YPEL1	0.325099597	6.71E-06	0.0001114	179	183
ZDHHC8	0.325099597	6.71E-06	0.0001114	179	183
ZNF74	0.325099597	6.71E-06	0.0001114	179	183
snoU13|EN	0.325099597	6.71E-06	0.0001114	179	183
snoU13|EN	0.325099597	6.71E-06	0.0001114	179	183
snoU13|EN	0.325099597	6.71E-06	0.0001114	179	183
MIR648	0.325203961	6.66E-06	0.0001114	179	183
PEX26	0.325499354	6.53E-06	0.0001114	179	183
PKDREJ	0.325886388	6.35E-06	0.0001114	179	183
TTC38	0.325886388	6.35E-06	0.0001114	179	183
GTSE1	0.325886388	6.35E-06	0.0001114	179	183
RUNX1T1	0.32591563	6.34E-06	0.0001114	179	181
HAS2	0.326056416	6.28E-06	0.0001114	179	181
KIAA1671	0.326304591	6.17E-06	0.00011018	179	183
TRMU	0.326328504	6.16E-06	0.00011007	179	183
FAM92A1	0.32645214	6.11E-06	0.00010921	179	181
RBM12B	0.32645214	6.11E-06	0.00010921	179	181
TMEM67	0.32645214	6.11E-06	0.00010921	179	181
PDP1	0.32645214	6.11E-06	0.00010921	179	181
MIR378D2	0.32645214	6.11E-06	0.00010921	179	181

CDH17	0.32645904	6.11E-06	0.00010921	179	181
GEM	0.32645904	6.11E-06	0.00010921	179	181
RN7SL757F	0.326608652	6.04E-06	0.00010858	179	182
SNORD42|E	0.326608652	6.04E-06	0.00010858	179	182
INTS8	0.326792311	5.97E-06	0.00010737	179	181
CCNE2	0.326792311	5.97E-06	0.00010737	179	181
NUP50	0.326871758	5.93E-06	0.00010693	179	183
SNORA40|E	0.326891189	5.93E-06	0.00010686	179	182
CCDC117	0.327060242	5.86E-06	0.00010569	179	182
XBP1	0.327060242	5.86E-06	0.00010569	179	182
ST3GAL1	0.327148858	5.82E-06	0.0001052	179	182
ESRP1	0.327382919	5.73E-06	0.00010358	179	181
GPIHBP1	0.327828248	5.55E-06	0.00010049	179	182
ZC3H3	0.328234889	5.40E-06	9.78E-05	179	183
snoU13|EN	0.328467016	5.31E-06	9.63E-05	179	181
RN7SKP231	0.328467016	5.31E-06	9.63E-05	179	181
SULT4A1	0.328490766	5.30E-06	9.62E-05	179	183
PNPLA5	0.328490766	5.30E-06	9.62E-05	179	183
RN7SL633F	0.328838487	5.17E-06	9.41E-05	179	182
SMTN	0.328838487	5.17E-06	9.41E-05	179	182
INPP5J	0.328838487	5.17E-06	9.41E-05	179	182
PLA2G3	0.328838487	5.17E-06	9.41E-05	179	182
MIR3928	0.328838487	5.17E-06	9.41E-05	179	182
RNF185	0.328838487	5.17E-06	9.41E-05	179	182
LIMK2	0.328838487	5.17E-06	9.41E-05	179	182
PIK3IP1	0.328838487	5.17E-06	9.41E-05	179	182
RNA5SP496	0.328838487	5.17E-06	9.41E-05	179	182
PATZ1	0.328838487	5.17E-06	9.41E-05	179	182
PNPLA3	0.329103371	5.08E-06	9.30E-05	179	183
SAMM50	0.329103371	5.08E-06	9.30E-05	179	183
CABP7	0.329290558	5.01E-06	9.19E-05	179	182
ZMAT5	0.329290558	5.01E-06	9.19E-05	179	182
UQCR10	0.329290558	5.01E-06	9.19E-05	179	182
ASCC2	0.329290558	5.01E-06	9.19E-05	179	182
MTMR3	0.329290558	5.01E-06	9.19E-05	179	182
TUG1	0.329621581	4.90E-06	9.02E-05	179	182
snoU13|EN	0.329624159	4.90E-06	9.02E-05	179	183
TIMP3	0.330121804	4.73E-06	8.72E-05	179	181
LINC00898	0.330195962	4.71E-06	8.68E-05	179	183
GSDMC	0.330216526	4.70E-06	8.67E-05	179	181
MAFA	0.330590584	4.58E-06	8.46E-05	179	182
EPPK1	0.330778733	4.52E-06	8.35E-05	179	183
PARP10	0.330874091	4.49E-06	8.30E-05	179	183
AP1B1	0.330978111	4.45E-06	8.25E-05	179	182
RFPL1S	0.330978111	4.45E-06	8.25E-05	179	182
RFPL1	0.330978111	4.45E-06	8.25E-05	179	182
NEFH	0.330978111	4.45E-06	8.25E-05	179	182
LIF	0.331207035	4.38E-06	8.14E-05	179	182
MGC20647	0.331207035	4.38E-06	8.14E-05	179	182
OSM	0.331207035	4.38E-06	8.14E-05	179	182
KIAA0930	0.331232397	4.37E-06	8.14E-05	179	183

MIR1249	0.331232397	4.37E-06	8.14E-05	179	183
GRINA	0.331422	4.32E-06	8.05E-05	179	183
SPATC1	0.331422	4.32E-06	8.05E-05	179	183
LY6E	0.331513502	4.29E-06	8.01E-05	179	182
C8orf31	0.331513502	4.29E-06	8.01E-05	179	182
SEC14L6	0.33154416	4.28E-06	8.00E-05	179	182
GAL3ST1	0.331595211	4.26E-06	7.98E-05	179	182
PES1	0.331595211	4.26E-06	7.98E-05	179	182
TCN2	0.331595211	4.26E-06	7.98E-05	179	182
SLC35E4	0.331595211	4.26E-06	7.98E-05	179	182
DUSP18	0.331595211	4.26E-06	7.98E-05	179	182
MIR3200	0.331595211	4.26E-06	7.98E-05	179	182
FSBP	0.332125784	4.11E-06	7.72E-05	179	181
RAD54B	0.332125784	4.11E-06	7.72E-05	179	181
KIAA1429	0.332125784	4.11E-06	7.72E-05	179	181
PARVB	0.332229166	4.08E-06	7.68E-05	179	183
XPNPEP3	0.332428871	4.02E-06	7.58E-05	179	181
UPK3A	0.332753156	3.93E-06	7.41E-05	179	183
FAM118A	0.332753156	3.93E-06	7.41E-05	179	183
ADCY8	0.332916262	3.88E-06	7.34E-05	179	181
ZFP41	0.333099915	3.83E-06	7.25E-05	179	182
GLI4	0.333099915	3.83E-06	7.25E-05	179	182
ZNF696	0.333099915	3.83E-06	7.25E-05	179	182
MED30	0.333104249	3.83E-06	7.25E-05	179	181
ARFGAP3	0.333555676	3.71E-06	7.04E-05	179	182
WISP1	0.333607207	3.70E-06	7.02E-05	179	182
NDRG1	0.333607207	3.70E-06	7.02E-05	179	182
MIR151A	0.333642364	3.69E-06	7.01E-05	179	182
PTK2	0.333642364	3.69E-06	7.01E-05	179	182
RNA5SP278	0.333642364	3.69E-06	7.01E-05	179	182
ARC	0.333730498	3.67E-06	6.99E-05	179	182
JRK	0.333730498	3.67E-06	6.99E-05	179	182
PSCA	0.333730498	3.67E-06	6.99E-05	179	182
LY6K	0.333730498	3.67E-06	6.99E-05	179	182
THEM6	0.333730498	3.67E-06	6.99E-05	179	182
SLURP1	0.333730498	3.67E-06	6.99E-05	179	182
LYPD2	0.333730498	3.67E-06	6.99E-05	179	182
LY6D	0.333730498	3.67E-06	6.99E-05	179	182
LYNX1	0.333730498	3.67E-06	6.99E-05	179	182
GML	0.333775127	3.65E-06	6.99E-05	179	182
CYP11B1	0.333775127	3.65E-06	6.99E-05	179	182
CYP11B2	0.333775127	3.65E-06	6.99E-05	179	182
THOC5	0.334014327	3.59E-06	6.91E-05	179	182
GPR20	0.334021388	3.59E-06	6.91E-05	179	182
LINC00051	0.334021388	3.59E-06	6.91E-05	179	182
MROH5	0.334021388	3.59E-06	6.91E-05	179	182
PTP4A3	0.334021388	3.59E-06	6.91E-05	179	182
SNORD5|EN	0.334021388	3.59E-06	6.91E-05	179	182
TSNARE1	0.334021388	3.59E-06	6.91E-05	179	182
LINC00534	0.334117709	3.57E-06	6.90E-05	179	181
RNA5SP273	0.334234258	3.54E-06	6.84E-05	179	181

LDOC1L	0.334237152	3.54E-06	6.84E-05	179	183
LINC00207	0.334237152	3.54E-06	6.84E-05	179	183
LINC00229	0.334237152	3.54E-06	6.84E-05	179	183
PRR5	0.334237152	3.54E-06	6.84E-05	179	183
ARHGAP8	0.334237152	3.54E-06	6.84E-05	179	183
TG	0.334332507	3.51E-06	6.83E-05	179	182
FAM135B	0.334468963	3.48E-06	6.77E-05	179	182
PHF21B	0.334522264	3.46E-06	6.75E-05	179	183
7SK|ENSG0	0.334686486	3.42E-06	6.67E-05	179	183
GSDMD	0.334686486	3.42E-06	6.67E-05	179	183
MROH6	0.334686486	3.42E-06	6.67E-05	179	183
EEF1D	0.334686486	3.42E-06	6.67E-05	179	183
NAPRT1	0.334686486	3.42E-06	6.67E-05	179	183
TIGD5	0.334686486	3.42E-06	6.67E-05	179	183
PYCRL	0.334686486	3.42E-06	6.67E-05	179	183
TSTA3	0.334686486	3.42E-06	6.67E-05	179	183
ZNF623	0.334686486	3.42E-06	6.67E-05	179	183
CYB5R3	0.33469131	3.42E-06	6.67E-05	179	182
ATP5L2	0.33469131	3.42E-06	6.67E-05	179	182
A4GALT	0.33469131	3.42E-06	6.67E-05	179	182
KIAA1644	0.334797101	3.40E-06	6.67E-05	179	183
PARVG	0.334819255	3.39E-06	6.67E-05	179	183
BAI1	0.335087664	3.33E-06	6.56E-05	179	182
EMID1	0.335579552	3.21E-06	6.34E-05	179	182
RHBDD3	0.335579552	3.21E-06	6.34E-05	179	182
EWSR1	0.335579552	3.21E-06	6.34E-05	179	182
GAS2L1	0.335579552	3.21E-06	6.34E-05	179	182
RASL10A	0.335579552	3.21E-06	6.34E-05	179	182
SNORD125	0.335579552	3.21E-06	6.34E-05	179	182
FAM203A	0.335688066	3.19E-06	6.32E-05	179	183
MROH1	0.335688066	3.19E-06	6.32E-05	179	183
MIR3201	0.335913641	3.14E-06	6.23E-05	179	183
SREBF2	0.336058918	3.10E-06	6.17E-05	179	182
CENPM	0.336058918	3.10E-06	6.17E-05	179	182
LINC00634	0.336058918	3.10E-06	6.17E-05	179	182
MIR33A	0.336058918	3.10E-06	6.17E-05	179	182
MIR378I	0.336058918	3.10E-06	6.17E-05	179	182
SEPT3	0.336058918	3.10E-06	6.17E-05	179	182
SHISA8	0.336058918	3.10E-06	6.17E-05	179	182
TNFRSF13C	0.336058918	3.10E-06	6.17E-05	179	182
WBP2NL	0.336058918	3.10E-06	6.17E-05	179	182
NAGA	0.336058918	3.10E-06	6.17E-05	179	182
FAM109B	0.336058918	3.10E-06	6.17E-05	179	182
snoU13|EN	0.336058918	3.10E-06	6.17E-05	179	182
NDUFA6	0.336058918	3.10E-06	6.17E-05	179	182
DENND3	0.336129217	3.09E-06	6.17E-05	179	182
MIR4535	0.336465113	3.01E-06	6.06E-05	179	183
ACR	0.336465113	3.01E-06	6.06E-05	179	183
ADM2	0.336465113	3.01E-06	6.06E-05	179	183
ALG12	0.336465113	3.01E-06	6.06E-05	179	183
ARSA	0.336465113	3.01E-06	6.06E-05	179	183

BRD1	0.336465113	3.01E-06	6.06E-05	179	183
C22orf34	0.336465113	3.01E-06	6.06E-05	179	183
CHKB	0.336465113	3.01E-06	6.06E-05	179	183
CPT1B	0.336465113	3.01E-06	6.06E-05	179	183
CRELD2	0.336465113	3.01E-06	6.06E-05	179	183
DENND6B	0.336465113	3.01E-06	6.06E-05	179	183
HDAC10	0.336465113	3.01E-06	6.06E-05	179	183
IL17REL	0.336465113	3.01E-06	6.06E-05	179	183
KLHDC7B	0.336465113	3.01E-06	6.06E-05	179	183
LMF2	0.336465113	3.01E-06	6.06E-05	179	183
MAPK11	0.336465113	3.01E-06	6.06E-05	179	183
MAPK12	0.336465113	3.01E-06	6.06E-05	179	183
MAPK8IP2	0.336465113	3.01E-06	6.06E-05	179	183
MIOX	0.336465113	3.01E-06	6.06E-05	179	183
MIR3667	0.336465113	3.01E-06	6.06E-05	179	183
MLC1	0.336465113	3.01E-06	6.06E-05	179	183
MOV10L1	0.336465113	3.01E-06	6.06E-05	179	183
NCAPH2	0.336465113	3.01E-06	6.06E-05	179	183
ODF3B	0.336465113	3.01E-06	6.06E-05	179	183
PANX2	0.336465113	3.01E-06	6.06E-05	179	183
PIM3	0.336465113	3.01E-06	6.06E-05	179	183
PLXNB2	0.336465113	3.01E-06	6.06E-05	179	183
PPP6R2	0.336465113	3.01E-06	6.06E-05	179	183
RABL2B	0.336465113	3.01E-06	6.06E-05	179	183
RN7SKP252	0.336465113	3.01E-06	6.06E-05	179	183
RN7SL500F	0.336465113	3.01E-06	6.06E-05	179	183
SBF1	0.336465113	3.01E-06	6.06E-05	179	183
SCO2	0.336465113	3.01E-06	6.06E-05	179	183
SHANK3	0.336465113	3.01E-06	6.06E-05	179	183
SYCE3	0.336465113	3.01E-06	6.06E-05	179	183
TRABD	0.336465113	3.01E-06	6.06E-05	179	183
TTLL8	0.336465113	3.01E-06	6.06E-05	179	183
TUBGCP6	0.336465113	3.01E-06	6.06E-05	179	183
TYMP	0.336465113	3.01E-06	6.06E-05	179	183
ZBED4	0.336465113	3.01E-06	6.06E-05	179	183
LY6H	0.336606975	2.98E-06	6.06E-05	179	182
TMEM64	0.33681952	2.94E-06	6.06E-05	179	181
ACO2	0.338229848	2.66E-06	5.52E-05	179	182
SNORA25|E	0.338290205	2.64E-06	5.50E-05	179	181
SLC30A8	0.338437243	2.62E-06	5.45E-05	179	181
RN7SL826F	0.338437243	2.62E-06	5.45E-05	179	181
SNORA31|E	0.338437243	2.62E-06	5.45E-05	179	181
FAM19A5	0.338438732	2.62E-06	5.45E-05	179	183
TEF	0.338550762	2.59E-06	5.42E-05	179	182
PHF20L1	0.338959305	2.52E-06	5.27E-05	179	182
MIR5194	0.339191766	2.48E-06	5.19E-05	179	181
TMEM71	0.339295467	2.46E-06	5.15E-05	179	182
MIR30B	0.339557952	2.41E-06	5.06E-05	179	182
MIR30D	0.339557952	2.41E-06	5.06E-05	179	182
PLEC	0.339565158	2.41E-06	5.06E-05	179	183
MIR661	0.339565158	2.41E-06	5.06E-05	179	183

CCDC166	0.339610429	2.40E-06	5.06E-05	179	183
FAM83H	0.339610429	2.40E-06	5.06E-05	179	183
MAPK15	0.339610429	2.40E-06	5.06E-05	179	183
MIR4664	0.339610429	2.40E-06	5.06E-05	179	183
ZNF707	0.339610429	2.40E-06	5.06E-05	179	183
KCNK9	0.339717673	2.38E-06	5.04E-05	179	182
C8orf87	0.339998122	2.34E-06	4.94E-05	179	181
OPLAH	0.340017704	2.33E-06	4.94E-05	179	183
EXOSC4	0.340017704	2.33E-06	4.94E-05	179	183
GPAA1	0.340017704	2.33E-06	4.94E-05	179	183
CYC1	0.340017704	2.33E-06	4.94E-05	179	183
SHARPIN	0.340017704	2.33E-06	4.94E-05	179	183
KIAA1875	0.340017704	2.33E-06	4.94E-05	179	183
MAF1	0.340017704	2.33E-06	4.94E-05	179	183
EXT1	0.34019639	2.30E-06	4.90E-05	179	181
RNA5SP274	0.340390149	2.27E-06	4.84E-05	179	181
EFCAB6	0.340449254	2.26E-06	4.82E-05	179	183
KHDRBS3	0.340869687	2.19E-06	4.68E-05	179	182
FBLN1	0.341426918	2.10E-06	4.50E-05	179	183
FAM49B	0.342060233	2.01E-06	4.30E-05	179	181
LINC00535	0.342212547	1.99E-06	4.26E-05	179	181
WNT7B	0.342523058	1.94E-06	4.16E-05	179	183
SLC45A4	0.342618376	1.93E-06	4.14E-05	179	182
SMC1B	0.342795648	1.90E-06	4.09E-05	179	183
RIBC2	0.342795648	1.90E-06	4.09E-05	179	183
LINC00899	0.342995034	1.88E-06	4.04E-05	179	183
C22orf26	0.342995034	1.88E-06	4.04E-05	179	183
C8orf17	0.343202413	1.85E-06	3.98E-05	179	182
ATXN10	0.343227169	1.84E-06	3.98E-05	179	183
MIR4762	0.343227169	1.84E-06	3.98E-05	179	183
NIPSNAP1	0.343242897	1.84E-06	3.98E-05	179	182
NF2	0.343242897	1.84E-06	3.98E-05	179	182
TRAPPC9	0.343937977	1.75E-06	3.79E-05	179	182
SNORA12|E	0.344152323	1.72E-06	3.73E-05	179	181
SLA	0.344250721	1.71E-06	3.71E-05	179	182
AGO2	0.344328078	1.70E-06	3.69E-05	179	182
TRIQK	0.345035723	1.61E-06	3.51E-05	179	181
MEI1	0.345343206	1.58E-06	3.43E-05	179	182
CCDC134	0.345343206	1.58E-06	3.43E-05	179	182
SLC25A17	0.345565412	1.55E-06	3.38E-05	179	181
MIR4766	0.345565412	1.55E-06	3.38E-05	179	181
ST13	0.346390891	1.46E-06	3.18E-05	179	181
DNAJB7	0.346390891	1.46E-06	3.18E-05	179	181
TOB2	0.347270771	1.37E-06	2.99E-05	179	182
MCHR1	0.347389749	1.36E-06	2.96E-05	179	181
RBFOX2	0.347502152	1.34E-06	2.94E-05	179	181
HPYR1	0.348628214	1.24E-06	2.71E-05	179	182
LRRC6	0.348628214	1.24E-06	2.71E-05	179	182
EFR3A	0.349146592	1.19E-06	2.61E-05	179	182
OC90	0.349146592	1.19E-06	2.61E-05	179	182
HHLA1	0.349146592	1.19E-06	2.61E-05	179	182

KCNQ3	0.349146592	1.19E-06	2.61E-05	179	182
ZFAT	0.349374079	1.17E-06	2.57E-05	179	182
CECR2	0.349425901	1.16E-06	2.57E-05	179	183
CCT8L2	0.349609875	1.15E-06	2.53E-05	179	183
HSFY1P1	0.349609875	1.15E-06	2.53E-05	179	183
KCNMB3P1	0.349609875	1.15E-06	2.53E-05	179	183
OR11H1	0.349609875	1.15E-06	2.53E-05	179	183
POTEH	0.349609875	1.15E-06	2.53E-05	179	183
TPTEP1	0.349609875	1.15E-06	2.53E-05	179	183
XKR3	0.349609875	1.15E-06	2.53E-05	179	183
GAB4	0.349609875	1.15E-06	2.53E-05	179	183
CECR7	0.349609875	1.15E-06	2.53E-05	179	183
IL17RA	0.349609875	1.15E-06	2.53E-05	179	183
CECR6	0.349609875	1.15E-06	2.53E-05	179	183
CECR5	0.349609875	1.15E-06	2.53E-05	179	183
CECR1	0.349609875	1.15E-06	2.53E-05	179	183
CECR3	0.349609875	1.15E-06	2.53E-05	179	183
CECR9	0.349609875	1.15E-06	2.53E-05	179	183
RN7SL843F	0.349609875	1.15E-06	2.53E-05	179	183
ELFN2	0.349712888	1.14E-06	2.53E-05	179	181
SNORA72|E	0.350786296	1.05E-06	2.35E-05	179	181
CYTH4	0.350793621	1.05E-06	2.35E-05	179	181
POLR3H	0.351442297	1.00E-06	2.24E-05	179	182
SLC25A18	0.356011391	7.06E-07	1.59E-05	179	183
ATP6V1E1	0.356011391	7.06E-07	1.59E-05	179	183
BCL2L13	0.356011391	7.06E-07	1.59E-05	179	183
MAFF	0.357217163	6.43E-07	1.45E-05	179	181
ADCK5	0.357962344	6.07E-07	1.37E-05	179	183
ARHGAP39	0.357962344	6.07E-07	1.37E-05	179	183
BOP1	0.357962344	6.07E-07	1.37E-05	179	183
C8orf33	0.357962344	6.07E-07	1.37E-05	179	183
C8orf82	0.357962344	6.07E-07	1.37E-05	179	183
COMMD5	0.357962344	6.07E-07	1.37E-05	179	183
CPSF1	0.357962344	6.07E-07	1.37E-05	179	183
CYHR1	0.357962344	6.07E-07	1.37E-05	179	183
DGAT1	0.357962344	6.07E-07	1.37E-05	179	183
FAM203B	0.357962344	6.07E-07	1.37E-05	179	183
FBXL6	0.357962344	6.07E-07	1.37E-05	179	183
FOXH1	0.357962344	6.07E-07	1.37E-05	179	183
GPT	0.357962344	6.07E-07	1.37E-05	179	183
HSF1	0.357962344	6.07E-07	1.37E-05	179	183
KIFC2	0.357962344	6.07E-07	1.37E-05	179	183
LRRC14	0.357962344	6.07E-07	1.37E-05	179	183
LRRC24	0.357962344	6.07E-07	1.37E-05	179	183
MFSD3	0.357962344	6.07E-07	1.37E-05	179	183
MIR1234	0.357962344	6.07E-07	1.37E-05	179	183
MIR939	0.357962344	6.07E-07	1.37E-05	179	183
PPP1R16A	0.357962344	6.07E-07	1.37E-05	179	183
RECQL4	0.357962344	6.07E-07	1.37E-05	179	183
RN7SL395F	0.357962344	6.07E-07	1.37E-05	179	183
RPL8	0.357962344	6.07E-07	1.37E-05	179	183

SCRT1	0.357962344	6.07E-07	1.37E-05	179	183
SCXA	0.357962344	6.07E-07	1.37E-05	179	183
SCXB	0.357962344	6.07E-07	1.37E-05	179	183
SLC39A4	0.357962344	6.07E-07	1.37E-05	179	183
SLC52A2	0.357962344	6.07E-07	1.37E-05	179	183
TMEM249	0.357962344	6.07E-07	1.37E-05	179	183
TONSL	0.357962344	6.07E-07	1.37E-05	179	183
VPS28	0.357962344	6.07E-07	1.37E-05	179	183
ZNF16	0.357962344	6.07E-07	1.37E-05	179	183
ZNF250	0.357962344	6.07E-07	1.37E-05	179	183
ZNF251	0.357962344	6.07E-07	1.37E-05	179	183
ZNF252P	0.357962344	6.07E-07	1.37E-05	179	183
ZNF34	0.357962344	6.07E-07	1.37E-05	179	183
ZNF517	0.357962344	6.07E-07	1.37E-05	179	183
ZNF7	0.357962344	6.07E-07	1.37E-05	179	183
MFNG	0.35841479	5.86E-07	1.37E-05	179	181
CARD10	0.35841479	5.86E-07	1.37E-05	179	181
CDC42EP1	0.35841479	5.86E-07	1.37E-05	179	181
LGALS2	0.35841479	5.86E-07	1.37E-05	179	181
GGA1	0.35841479	5.86E-07	1.37E-05	179	181
SH3BP1	0.35841479	5.86E-07	1.37E-05	179	181
PDXP	0.35841479	5.86E-07	1.37E-05	179	181
RN7SL385F	0.35841479	5.86E-07	1.37E-05	179	181
LGALS1	0.35841479	5.86E-07	1.37E-05	179	181
NOL12	0.35841479	5.86E-07	1.37E-05	179	181
TRIOBP	0.35841479	5.86E-07	1.37E-05	179	181
snoU13|EN	0.35841479	5.86E-07	1.37E-05	179	181
GCAT	0.35841479	5.86E-07	1.37E-05	179	181
H1F0	0.35841479	5.86E-07	1.37E-05	179	181
ANKRD54	0.35841479	5.86E-07	1.37E-05	179	181
GALR3	0.35841479	5.86E-07	1.37E-05	179	181
MIR658	0.35841479	5.86E-07	1.37E-05	179	181
EIF3L	0.35841479	5.86E-07	1.37E-05	179	181
MIR659	0.35841479	5.86E-07	1.37E-05	179	181
RBX1	0.358637084	5.76E-07	1.37E-05	179	181
TMEM184B	0.359756382	5.28E-07	1.26E-05	179	181
RN7SL704F	0.359756382	5.28E-07	1.26E-05	179	181
PHF5A	0.360693998	4.91E-07	1.17E-05	179	182
APOL1	0.360819777	4.86E-07	1.16E-05	179	181
APOL2	0.360819777	4.86E-07	1.16E-05	179	181
MYH9	0.360819777	4.86E-07	1.16E-05	179	181
RN7SL349F	0.360819777	4.86E-07	1.16E-05	179	181
TXN2	0.360819777	4.86E-07	1.16E-05	179	181
FOXRED2	0.360819777	4.86E-07	1.16E-05	179	181
EIF3D	0.360819777	4.86E-07	1.16E-05	179	181
CSDC2	0.362452832	4.28E-07	1.03E-05	179	182
PMM1	0.362452832	4.28E-07	1.03E-05	179	182
DESI1	0.362452832	4.28E-07	1.03E-05	179	182
XRCC6	0.362452832	4.28E-07	1.03E-05	179	182
NHP2L1	0.362452832	4.28E-07	1.03E-05	179	182
C22orf46	0.362452832	4.28E-07	1.03E-05	179	182

RNU6ATAC2	0.362452832	4.28E-07	1.03E-05	179	182
snoU13|EN	0.362727916	4.19E-07	1.01E-05	179	181
MCM5	0.363640917	3.90E-07	9.45E-06	179	181
C1QTNF6	0.363871258	3.83E-07	9.29E-06	179	181
SSTR3	0.363871258	3.83E-07	9.29E-06	179	181
RAC2	0.363871258	3.83E-07	9.29E-06	179	181
snoU13|EN	0.364077518	3.77E-07	9.17E-06	179	181
IFT27	0.364277737	3.71E-07	9.03E-06	179	181
PVALB	0.364277737	3.71E-07	9.03E-06	179	181
NCF4	0.364277737	3.71E-07	9.03E-06	179	181
CSF2RB	0.364277737	3.71E-07	9.03E-06	179	181
CACNG2	0.364494461	3.64E-07	8.91E-06	179	181
MKL1	0.36523627	3.44E-07	8.41E-06	179	181
RASD2	0.366165519	3.19E-07	7.82E-06	179	181
APOL6	0.366165519	3.19E-07	7.82E-06	179	181
MB	0.366165519	3.19E-07	7.82E-06	179	181
APOL5	0.366165519	3.19E-07	7.82E-06	179	181
CASC7	0.367775549	2.81E-07	6.91E-06	179	182
CHRAC1	0.367775549	2.81E-07	6.91E-06	179	182
SLC16A8	0.36849375	2.65E-07	6.54E-06	179	181
BAIAP2L2	0.36849375	2.65E-07	6.54E-06	179	181
HMOX1	0.370427771	2.27E-07	5.61E-06	179	181
PDGFB	0.371052241	2.16E-07	5.34E-06	179	181
ISX	0.371490025	2.08E-07	5.15E-06	179	181
HMGXB4	0.371490025	2.08E-07	5.15E-06	179	181
TOM1	0.371742389	2.04E-07	5.06E-06	179	181
MIR3909	0.371742389	2.04E-07	5.06E-06	179	181
MICALL1	0.372077108	1.99E-07	4.94E-06	179	181
C22orf23	0.372077108	1.99E-07	4.94E-06	179	181
SOX10	0.372077108	1.99E-07	4.94E-06	179	181
MIR4534	0.372077108	1.99E-07	4.94E-06	179	181
MIR1281	0.37223773	1.96E-07	4.89E-06	179	181
APOL3	0.372646853	1.90E-07	4.74E-06	179	181
APOL4	0.372646853	1.90E-07	4.74E-06	179	181
TEX33	0.372734808	1.88E-07	4.71E-06	179	181
TST	0.372734808	1.88E-07	4.71E-06	179	181
MPST	0.372734808	1.88E-07	4.71E-06	179	181
KCTD17	0.372734808	1.88E-07	4.71E-06	179	181
RN7SKP214	0.372734808	1.88E-07	4.71E-06	179	181
TMPRSS6	0.372734808	1.88E-07	4.71E-06	179	181
IL2RB	0.372734808	1.88E-07	4.71E-06	179	181
PLA2G6	0.373026484	1.84E-07	4.63E-06	179	181
EP300	0.373540425	1.76E-07	4.45E-06	179	182
L3MBTL2	0.373540425	1.76E-07	4.45E-06	179	182
CHADL	0.373540425	1.76E-07	4.45E-06	179	182
RANGAP1	0.373540425	1.76E-07	4.45E-06	179	182
ZC3H7B	0.37397002	1.70E-07	4.31E-06	179	182
PICK1	0.374917464	1.58E-07	4.00E-06	179	181
POLR2F	0.376519299	1.38E-07	3.51E-06	179	181
ADSL	0.382415352	8.46E-08	2.15E-06	179	181
SGSM3	0.382415352	8.46E-08	2.15E-06	179	181

NPTXR	0.384292484	7.22E-08	1.84E-06	179	181
CBX6	0.384292484	7.22E-08	1.84E-06	179	181
TNRC6B	0.384486091	7.11E-08	1.81E-06	179	181
FAM83F	0.392979953	3.43E-08	8.76E-07	179	181
APOBEC3C	0.396273204	2.57E-08	6.58E-07	179	181
APOBEC3D	0.396273204	2.57E-08	6.58E-07	179	181
APOBEC3F	0.396273204	2.57E-08	6.58E-07	179	181
APOBEC3G	0.396273204	2.57E-08	6.58E-07	179	181
APOBEC3H	0.396273204	2.57E-08	6.58E-07	179	181
CBX7	0.396336777	2.56E-08	6.57E-07	179	181
RPL3	0.398318121	2.15E-08	5.52E-07	179	181
SNORD83B	0.398318121	2.15E-08	5.52E-07	179	181
SNORD83A	0.398318121	2.15E-08	5.52E-07	179	181
SNORD43|E	0.398318121	2.15E-08	5.52E-07	179	181
SYNGR1	0.398318121	2.15E-08	5.52E-07	179	181
TOMM22	0.398469155	2.12E-08	5.48E-07	179	181
JOSD1	0.398469155	2.12E-08	5.48E-07	179	181
GTPBP1	0.398469155	2.12E-08	5.48E-07	179	181
SUN2	0.398469155	2.12E-08	5.48E-07	179	181
DNAL4	0.398469155	2.12E-08	5.48E-07	179	181
APOBEC3A	0.398483795	2.12E-08	5.48E-07	179	181
APOBEC3B	0.398483795	2.12E-08	5.48E-07	179	181
TAB1	0.399195615	1.99E-08	5.18E-07	179	181
MGAT3	0.399195615	1.99E-08	5.18E-07	179	181
SMCR7L	0.399195615	1.99E-08	5.18E-07	179	181
ATF4	0.399195615	1.99E-08	5.18E-07	179	181
RPS19BP1	0.399195615	1.99E-08	5.18E-07	179	181
KDELR3	0.400886837	1.71E-08	4.48E-07	179	181
DDX17	0.400886837	1.71E-08	4.48E-07	179	181
DMC1	0.400886837	1.71E-08	4.48E-07	179	181
FAM227A	0.400886837	1.71E-08	4.48E-07	179	181
CBY1	0.400886837	1.71E-08	4.48E-07	179	181
CACNA1I	0.401499638	1.62E-08	4.26E-07	179	181
ENTHD1	0.401499638	1.62E-08	4.26E-07	179	181
RN7SKP21C	0.401499638	1.62E-08	4.26E-07	179	181
GRAP2	0.401499638	1.62E-08	4.26E-07	179	181
CSNK1E	0.411733855	6.38E-09	1.69E-07	179	181
KCNJ4	0.411733855	6.38E-09	1.69E-07	179	181
ABHD4	0.509007432	1.61E-13	4.27E-12	179	178
DAD1	0.509007432	1.61E-13	4.27E-12	179	178
OR4E2	0.509007432	1.61E-13	4.27E-12	179	178
OR6J1	0.509007432	1.61E-13	4.27E-12	179	178
TRAC	0.509007432	1.61E-13	4.27E-12	179	178
TRAJ10	0.509007432	1.61E-13	4.27E-12	179	178
TRAJ11	0.509007432	1.61E-13	4.27E-12	179	178
TRAJ12	0.509007432	1.61E-13	4.27E-12	179	178
TRAJ13	0.509007432	1.61E-13	4.27E-12	179	178
TRAJ14	0.509007432	1.61E-13	4.27E-12	179	178
TRAJ16	0.509007432	1.61E-13	4.27E-12	179	178
TRAJ17	0.509007432	1.61E-13	4.27E-12	179	178
TRAJ18	0.509007432	1.61E-13	4.27E-12	179	178

TRAJ19	0.509007432	1.61E-13	4.27E-12	179	178
TRAJ1	0.509007432	1.61E-13	4.27E-12	179	178
TRAJ20	0.509007432	1.61E-13	4.27E-12	179	178
TRAJ21	0.509007432	1.61E-13	4.27E-12	179	178
TRAJ22	0.509007432	1.61E-13	4.27E-12	179	178
TRAJ23	0.509007432	1.61E-13	4.27E-12	179	178
TRAJ24	0.509007432	1.61E-13	4.27E-12	179	178
TRAJ25	0.509007432	1.61E-13	4.27E-12	179	178
TRAJ26	0.509007432	1.61E-13	4.27E-12	179	178
TRAJ27	0.509007432	1.61E-13	4.27E-12	179	178
TRAJ28	0.509007432	1.61E-13	4.27E-12	179	178
TRAJ29	0.509007432	1.61E-13	4.27E-12	179	178
TRAJ2	0.509007432	1.61E-13	4.27E-12	179	178
TRAJ30	0.509007432	1.61E-13	4.27E-12	179	178
TRAJ31	0.509007432	1.61E-13	4.27E-12	179	178
TRAJ32	0.509007432	1.61E-13	4.27E-12	179	178
TRAJ33	0.509007432	1.61E-13	4.27E-12	179	178
TRAJ34	0.509007432	1.61E-13	4.27E-12	179	178
TRAJ35	0.509007432	1.61E-13	4.27E-12	179	178
TRAJ36	0.509007432	1.61E-13	4.27E-12	179	178
TRAJ37	0.509007432	1.61E-13	4.27E-12	179	178
TRAJ38	0.509007432	1.61E-13	4.27E-12	179	178
TRAJ39	0.509007432	1.61E-13	4.27E-12	179	178
TRAJ3	0.509007432	1.61E-13	4.27E-12	179	178
TRAJ40	0.509007432	1.61E-13	4.27E-12	179	178
TRAJ41	0.509007432	1.61E-13	4.27E-12	179	178
TRAJ42	0.509007432	1.61E-13	4.27E-12	179	178
TRAJ43	0.509007432	1.61E-13	4.27E-12	179	178
TRAJ44	0.509007432	1.61E-13	4.27E-12	179	178
TRAJ45	0.509007432	1.61E-13	4.27E-12	179	178
TRAJ46	0.509007432	1.61E-13	4.27E-12	179	178
TRAJ47	0.509007432	1.61E-13	4.27E-12	179	178
TRAJ48	0.509007432	1.61E-13	4.27E-12	179	178
TRAJ49	0.509007432	1.61E-13	4.27E-12	179	178
TRAJ4	0.509007432	1.61E-13	4.27E-12	179	178
TRAJ50	0.509007432	1.61E-13	4.27E-12	179	178
TRAJ52	0.509007432	1.61E-13	4.27E-12	179	178
TRAJ53	0.509007432	1.61E-13	4.27E-12	179	178
TRAJ54	0.509007432	1.61E-13	4.27E-12	179	178
TRAJ56	0.509007432	1.61E-13	4.27E-12	179	178
TRAJ57	0.509007432	1.61E-13	4.27E-12	179	178
TRAJ58	0.509007432	1.61E-13	4.27E-12	179	178
TRAJ59	0.509007432	1.61E-13	4.27E-12	179	178
TRAJ5	0.509007432	1.61E-13	4.27E-12	179	178
TRAJ61	0.509007432	1.61E-13	4.27E-12	179	178
TRAJ6	0.509007432	1.61E-13	4.27E-12	179	178
TRAJ7	0.509007432	1.61E-13	4.27E-12	179	178
TRAJ8	0.509007432	1.61E-13	4.27E-12	179	178
TRAJ9	0.509007432	1.61E-13	4.27E-12	179	178
TRAV10	0.509007432	1.61E-13	4.27E-12	179	178
TRAV14DV4	0.509007432	1.61E-13	4.27E-12	179	178

TRAV16	0.509007432	1.61E-13	4.27E-12	179	178
TRAV17	0.509007432	1.61E-13	4.27E-12	179	178
TRAV18	0.509007432	1.61E-13	4.27E-12	179	178
TRAV19	0.509007432	1.61E-13	4.27E-12	179	178
TRAV20	0.509007432	1.61E-13	4.27E-12	179	178
TRAV21	0.509007432	1.61E-13	4.27E-12	179	178
TRAV22	0.509007432	1.61E-13	4.27E-12	179	178
TRAV23DV6	0.509007432	1.61E-13	4.27E-12	179	178
TRAV24	0.509007432	1.61E-13	4.27E-12	179	178
TRAV25	0.509007432	1.61E-13	4.27E-12	179	178
TRAV27	0.509007432	1.61E-13	4.27E-12	179	178
TRAV29DV5	0.509007432	1.61E-13	4.27E-12	179	178
TRAV2	0.509007432	1.61E-13	4.27E-12	179	178
TRAV30	0.509007432	1.61E-13	4.27E-12	179	178
TRAV34	0.509007432	1.61E-13	4.27E-12	179	178
TRAV35	0.509007432	1.61E-13	4.27E-12	179	178
TRAV36DV7	0.509007432	1.61E-13	4.27E-12	179	178
TRAV39	0.509007432	1.61E-13	4.27E-12	179	178
TRAV3	0.509007432	1.61E-13	4.27E-12	179	178
TRAV40	0.509007432	1.61E-13	4.27E-12	179	178
TRAV41	0.509007432	1.61E-13	4.27E-12	179	178
TRAV4	0.509007432	1.61E-13	4.27E-12	179	178
TRAV5	0.509007432	1.61E-13	4.27E-12	179	178
TRAV6	0.509007432	1.61E-13	4.27E-12	179	178
TRAV7	0.509007432	1.61E-13	4.27E-12	179	178
TRDC	0.509007432	1.61E-13	4.27E-12	179	178
TRDD1	0.509007432	1.61E-13	4.27E-12	179	178
TRDD2	0.509007432	1.61E-13	4.27E-12	179	178
TRDD3	0.509007432	1.61E-13	4.27E-12	179	178
TRDJ1	0.509007432	1.61E-13	4.27E-12	179	178
TRDJ2	0.509007432	1.61E-13	4.27E-12	179	178
TRDJ3	0.509007432	1.61E-13	4.27E-12	179	178
TRDJ4	0.509007432	1.61E-13	4.27E-12	179	178
TRDV1	0.509007432	1.61E-13	4.27E-12	179	178
TRDV2	0.509007432	1.61E-13	4.27E-12	179	178
TRDV3	0.509007432	1.61E-13	4.27E-12	179	178
OR10G2	0.525311112	1.92E-14	5.70E-13	179	178
OXA1L	0.52613564	1.72E-14	5.11E-13	179	178
SLC7A7	0.52613564	1.72E-14	5.11E-13	179	178
SNORD41|E	0.52613564	1.72E-14	5.11E-13	179	178
SALL2	0.529086022	1.16E-14	3.45E-13	179	178
OR10G3	0.529086022	1.16E-14	3.45E-13	179	178
RN7SL650F	0.533470658	6.35E-15	1.90E-13	179	178
RAB2B	0.533470658	6.35E-15	1.90E-13	179	178
TOX4	0.533470658	6.35E-15	1.90E-13	179	178
METTL3	0.533470658	6.35E-15	1.90E-13	179	178
RPGRIP1	0.534117436	5.81E-15	1.75E-13	179	178
SUPT16H	0.534117436	5.81E-15	1.75E-13	179	178
CHD8	0.534117436	5.81E-15	1.75E-13	179	178
SNORD9	0.534117436	5.81E-15	1.75E-13	179	178
SNORD8	0.534117436	5.81E-15	1.75E-13	179	178

OR5AU1	0.535358079	4.90E-15	1.48E-13	179	178
LINC00641	0.535358079	4.90E-15	1.48E-13	179	178
MRPL52	0.541996233	1.93E-15	5.86E-14	179	178
MMP14	0.541996233	1.93E-15	5.86E-14	179	178
LRP10	0.541996233	1.93E-15	5.86E-14	179	178
REM2	0.541996233	1.93E-15	5.86E-14	179	178
RBM23	0.541996233	1.93E-15	5.86E-14	179	178
PRMT5	0.541996233	1.93E-15	5.86E-14	179	178
HAUS4	0.541996233	1.93E-15	5.86E-14	179	178
MIR4707	0.541996233	1.93E-15	5.86E-14	179	178
AJUBA	0.543023039	1.67E-15	5.11E-14	179	178
C14orf93	0.543023039	1.67E-15	5.11E-14	179	178
PSMB5	0.543023039	1.67E-15	5.11E-14	179	178
SNORA73|E	0.543023039	1.67E-15	5.11E-14	179	178
PSMB11	0.543023039	1.67E-15	5.11E-14	179	178
CDH24	0.543023039	1.67E-15	5.11E-14	179	178
ACIN1	0.543023039	1.67E-15	5.11E-14	179	178
HNRNPC	0.543339419	1.60E-15	4.93E-14	179	178
OR11H4	0.544926563	1.28E-15	3.94E-14	179	178
KLHL33	0.549105062	6.99E-16	2.16E-14	179	178
OSGEP	0.549105062	6.99E-16	2.16E-14	179	178
TTC5	0.549815926	6.31E-16	1.96E-14	179	178
CCNB1IP1	0.549815926	6.31E-16	1.96E-14	179	178
SNORA79|E	0.549815926	6.31E-16	1.96E-14	179	178
SNORD126	0.549815926	6.31E-16	1.96E-14	179	178
RPPH1	0.549815926	6.31E-16	1.96E-14	179	178
PARP2	0.549815926	6.31E-16	1.96E-14	179	178
TEP1	0.549815926	6.31E-16	1.96E-14	179	178
RNA5SP382	0.549815926	6.31E-16	1.96E-14	179	178
RN7SL189F	0.551833199	4.70E-16	1.47E-14	179	178
RNASE2	0.551833199	4.70E-16	1.47E-14	179	178
RNASE3	0.551833199	4.70E-16	1.47E-14	179	178
METTL17	0.551833199	4.70E-16	1.47E-14	179	178
SLC39A2	0.551833199	4.70E-16	1.47E-14	179	178
NDRG2	0.551833199	4.70E-16	1.47E-14	179	178
TPPP2	0.551833199	4.70E-16	1.47E-14	179	178
RNASE13	0.551833199	4.70E-16	1.47E-14	179	178
RNASE7	0.551833199	4.70E-16	1.47E-14	179	178
RNASE8	0.551833199	4.70E-16	1.47E-14	179	178
ARHGEF40	0.551833199	4.70E-16	1.47E-14	179	178
ZNF219	0.551833199	4.70E-16	1.47E-14	179	178
TMEM253	0.551833199	4.70E-16	1.47E-14	179	178
EDDM3A	0.552859307	4.05E-16	1.29E-14	179	178
LINC00516	0.552924275	4.01E-16	1.28E-14	179	178
MED15P1	0.552924275	4.01E-16	1.28E-14	179	178
MED15P6	0.552924275	4.01E-16	1.28E-14	179	178
OR11H12	0.552924275	4.01E-16	1.28E-14	179	178
OR4K13	0.552924275	4.01E-16	1.28E-14	179	178
OR4K14	0.552924275	4.01E-16	1.28E-14	179	178
OR4K15	0.552924275	4.01E-16	1.28E-14	179	178
OR4K1	0.552924275	4.01E-16	1.28E-14	179	178

OR4K2	0.552924275	4.01E-16	1.28E-14	179	178
OR4K5	0.552924275	4.01E-16	1.28E-14	179	178
OR4M1	0.552924275	4.01E-16	1.28E-14	179	178
OR4N2	0.552924275	4.01E-16	1.28E-14	179	178
OR4Q2	0.552924275	4.01E-16	1.28E-14	179	178
OR4Q3	0.552924275	4.01E-16	1.28E-14	179	178
POTEG	0.552924275	4.01E-16	1.28E-14	179	178
POTEM	0.552924275	4.01E-16	1.28E-14	179	178
snoU13|EN	0.552924275	4.01E-16	1.28E-14	179	178
OR4L1	0.552924275	4.01E-16	1.28E-14	179	178
RNA5SP380	0.552924275	4.01E-16	1.28E-14	179	178
OR4K17	0.552924275	4.01E-16	1.28E-14	179	178
OR4N5	0.552924275	4.01E-16	1.28E-14	179	178
RNA5SP381	0.552924275	4.01E-16	1.28E-14	179	178
OR11G2	0.552924275	4.01E-16	1.28E-14	179	178
OR11H6	0.552924275	4.01E-16	1.28E-14	179	178
OR11H7	0.552924275	4.01E-16	1.28E-14	179	178
EDDM3B	0.553144424	3.88E-16	1.28E-14	179	178
RNASE6	0.553144424	3.88E-16	1.28E-14	179	178
RNASE1	0.553144424	3.88E-16	1.28E-14	179	178
C14orf119	0.554367996	3.24E-16	1.07E-14	179	178
CEBPE	0.554367996	3.24E-16	1.07E-14	179	178
SLC7A8	0.554367996	3.24E-16	1.07E-14	179	178
RNASE11	0.555969799	2.56E-16	8.49E-15	179	178
APEX1	0.556642348	2.31E-16	7.70E-15	179	178
TMEM55B	0.556642348	2.31E-16	7.70E-15	179	178
PNP	0.556642348	2.31E-16	7.70E-15	179	178
OR6S1	0.558858319	1.66E-16	5.55E-15	179	178
ANG	0.558858319	1.66E-16	5.55E-15	179	178
RNASE4	0.558858319	1.66E-16	5.55E-15	179	178
RNASE12	0.560000443	1.40E-16	4.70E-15	179	178
STXBP6	0.561591821	1.10E-16	3.70E-15	179	178
RNASE10	0.562912022	9.03E-17	3.04E-15	179	178
RNASE9	0.562912022	9.03E-17	3.04E-15	179	178
C14orf164	0.566336438	5.36E-17	1.81E-15	179	178
HOMEZ	0.566336438	5.36E-17	1.81E-15	179	178
PPP1R3E	0.566336438	5.36E-17	1.81E-15	179	178
BCL2L2	0.566336438	5.36E-17	1.81E-15	179	178
PABPN1	0.569821417	3.13E-17	1.06E-15	179	178
SLC22A17	0.569821417	3.13E-17	1.06E-15	179	178
EFS	0.569821417	3.13E-17	1.06E-15	179	178
DHRS2	0.569837792	3.12E-17	1.06E-15	179	178
IL25	0.569957232	3.06E-17	1.04E-15	179	178
CMTM5	0.569957232	3.06E-17	1.04E-15	179	178
MYH6	0.569957232	3.06E-17	1.04E-15	179	178
MIR208A	0.569957232	3.06E-17	1.04E-15	179	178
MYH7	0.569957232	3.06E-17	1.04E-15	179	178
MIR208B	0.569957232	3.06E-17	1.04E-15	179	178
NGDN	0.569957232	3.06E-17	1.04E-15	179	178
ZFHX2	0.569957232	3.06E-17	1.04E-15	179	178
THTPA	0.569957232	3.06E-17	1.04E-15	179	178

AP1G2	0.569957232	3.06E-17	1.04E-15	179	178
JPH4	0.569957232	3.06E-17	1.04E-15	179	178
CTSG	0.570858819	2.66E-17	9.21E-16	179	178
GZMH	0.570858819	2.66E-17	9.21E-16	179	178
GZMB	0.570858819	2.66E-17	9.21E-16	179	178
DHRS4L1	0.571060135	2.58E-17	8.96E-16	179	178
LRRC16B	0.571060135	2.58E-17	8.96E-16	179	178
RN7SKP205	0.57113912	2.55E-17	8.88E-16	179	178
LINC00596	0.57113912	2.55E-17	8.88E-16	179	178
DHRS4	0.575241531	1.34E-17	4.67E-16	179	178
DHRS4L2	0.575730853	1.24E-17	4.33E-16	179	178
DHRS1	0.58205737	4.49E-18	1.58E-16	179	178
NOP9	0.58205737	4.49E-18	1.58E-16	179	178
CIDEB	0.58205737	4.49E-18	1.58E-16	179	178
LTB4R2	0.58205737	4.49E-18	1.58E-16	179	178
LTB4R	0.58205737	4.49E-18	1.58E-16	179	178
ADCY4	0.58205737	4.49E-18	1.58E-16	179	178
RIPK3	0.58205737	4.49E-18	1.58E-16	179	178
NFATC4	0.58205737	4.49E-18	1.58E-16	179	178
NYNRIN	0.58205737	4.49E-18	1.58E-16	179	178
CBLN3	0.58205737	4.49E-18	1.58E-16	179	178
TINF2	0.582078561	4.48E-18	1.58E-16	179	178
TGM1	0.582078561	4.48E-18	1.58E-16	179	178
RABGGTA	0.582078561	4.48E-18	1.58E-16	179	178
NRL	0.582180664	4.41E-18	1.57E-16	179	178
PCK2	0.582180664	4.41E-18	1.57E-16	179	178
DCAF11	0.582180664	4.41E-18	1.57E-16	179	178
FITM1	0.582180664	4.41E-18	1.57E-16	179	178
EMC9	0.582180664	4.41E-18	1.57E-16	179	178
PSME1	0.582180664	4.41E-18	1.57E-16	179	178
PSME2	0.582180664	4.41E-18	1.57E-16	179	178
RNF31	0.582180664	4.41E-18	1.57E-16	179	178
IRF9	0.582180664	4.41E-18	1.57E-16	179	178
RNA5SP383	0.582180664	4.41E-18	1.57E-16	179	178
REC8	0.582180664	4.41E-18	1.57E-16	179	178
IPO4	0.582180664	4.41E-18	1.57E-16	179	178
TM9SF1	0.582180664	4.41E-18	1.57E-16	179	178
CHMP4A	0.582180664	4.41E-18	1.57E-16	179	178
TSSK4	0.582180664	4.41E-18	1.57E-16	179	178
MDP1	0.582180664	4.41E-18	1.57E-16	179	178
NEDD8	0.582180664	4.41E-18	1.57E-16	179	178
GMPR2	0.582180664	4.41E-18	1.57E-16	179	178
CPNE6	0.582198002	4.39E-18	1.57E-16	179	178
KHNYN	0.583422272	3.60E-18	1.32E-16	179	178
SDR39U1	0.583422272	3.60E-18	1.32E-16	179	178
CMA1	0.583422272	3.60E-18	1.32E-16	179	178
SNORD37|E	0.588185178	1.65E-18	6.08E-17	179	178
MIR4307	0.593924029	6.33E-19	2.34E-17	179	178
NOVA1	0.596069239	4.40E-19	1.63E-17	179	178
LINC00609	0.598576636	2.87E-19	1.06E-17	179	179
GPR33	0.598818023	2.75E-19	1.02E-17	179	179

NUBPL	0.599343946	2.51E-19	9.34E-18	179	179
BNIP3P1	0.600690254	1.99E-19	7.42E-18	179	178
snoU13|EN	0.602164572	1.54E-19	5.76E-18	179	179
BRMS1L	0.602164572	1.54E-19	5.76E-18	179	179
RALGAPA1	0.602240667	1.52E-19	5.70E-18	179	179
LINC0064E	0.60332181	1.26E-19	4.73E-18	179	178
AKAP6	0.604468609	1.03E-19	3.88E-18	179	179
BAZ1A	0.611975744	2.73E-20	1.03E-18	179	179
HECTD1	0.612133763	2.66E-20	9.98E-19	179	179
MIPOL1	0.612178877	2.63E-20	9.92E-19	179	179
STRN3	0.612201189	2.62E-20	9.89E-19	179	179
CFL2	0.612226184	2.61E-20	9.86E-19	179	179
G2E3	0.613026582	2.26E-20	8.55E-19	179	178
SCFD1	0.613026582	2.26E-20	8.55E-19	179	178
PRKD1	0.614149121	1.85E-20	7.00E-19	179	178
RN7SL660F	0.614515376	1.73E-20	6.56E-19	179	179
AP4S1	0.615239205	1.51E-20	5.77E-19	179	179
MIR624	0.615306631	1.50E-20	5.70E-19	179	179
DPPA3P2	0.616345029	1.24E-20	4.73E-19	179	179
SFTA3	0.616345029	1.24E-20	4.73E-19	179	179
SLC25A21	0.616382964	1.23E-20	4.71E-19	179	179
PTCSC3	0.616420457	1.22E-20	4.69E-19	179	179
RN7SKP21	0.616420457	1.22E-20	4.69E-19	179	179
MBIP	0.616420457	1.22E-20	4.69E-19	179	179
NFKBIA	0.617832481	9.43E-21	3.64E-19	179	179
INSM2	0.617832481	9.43E-21	3.64E-19	179	179
COCH	0.618129182	8.93E-21	3.45E-19	179	179
SNORA31|E	0.618137825	8.92E-21	3.45E-19	179	179
MIR4503	0.618557381	8.26E-21	3.20E-19	179	179
ARHGAP5	0.620168266	6.14E-21	2.38E-19	179	179
HEATR5A	0.62145195	4.84E-21	1.88E-19	179	179
DTD2	0.621512634	4.78E-21	1.86E-19	179	179
C14orf23	0.623768385	3.14E-21	1.22E-19	179	178
FOXG1	0.623768385	3.14E-21	1.22E-19	179	178
PAX9	0.62387495	3.08E-21	1.20E-19	179	179
KIAA0391	0.624909396	2.53E-21	9.93E-20	179	179
PSMA6	0.624909396	2.53E-21	9.93E-20	179	179
SRP54	0.625033352	2.48E-21	9.73E-20	179	179
FAM177A1	0.625033352	2.48E-21	9.73E-20	179	179
PPP2R3C	0.625033352	2.48E-21	9.73E-20	179	179
RN7SKP257	0.625123895	2.43E-21	9.62E-20	179	179
KLHL28	0.632830737	5.59E-22	2.21E-20	179	179
NPAS3	0.635368009	3.41E-22	1.35E-20	179	179
MIR5580	0.636259126	2.86E-22	1.14E-20	179	179
BMP4	0.636259126	2.86E-22	1.14E-20	179	179
EAPP	0.640285116	1.29E-22	5.15E-21	179	179
SNX6	0.64097475	1.13E-22	4.50E-21	179	179
GPHN	0.641596978	9.95E-23	3.98E-21	179	179
RN7SL3	0.642707223	7.96E-23	3.19E-21	179	179
RN7SL2	0.642707223	7.96E-23	3.19E-21	179	179
ARF6	0.642707223	7.96E-23	3.19E-21	179	179

C14orf28	0.642845515	7.75E-23	3.12E-21	179	179
C14orf182	0.643096678	7.37E-23	2.97E-21	179	179
SOS2	0.643130391	7.32E-23	2.95E-21	179	179
RN7SKP193	0.643183369	7.24E-23	2.93E-21	179	179
C14orf183	0.643183369	7.24E-23	2.93E-21	179	179
METTL21D	0.643183369	7.24E-23	2.93E-21	179	179
SPTSSA	0.643188287	7.23E-23	2.93E-21	179	179
NEMF	0.644372652	5.69E-23	2.32E-21	179	179
CDKN3	0.650732295	1.55E-23	6.32E-22	179	179
CNIH	0.650732295	1.55E-23	6.32E-22	179	179
GMFB	0.650732295	1.55E-23	6.32E-22	179	179
LRFN5	0.651125292	1.43E-23	5.86E-22	179	179
SAMD4A	0.651839044	1.23E-23	5.06E-22	179	179
GCH1	0.651839044	1.23E-23	5.06E-22	179	179
RNU6ATAC9	0.651839044	1.23E-23	5.06E-22	179	179
EGLN3	0.652480555	1.08E-23	4.45E-22	179	179
CLEC14A	0.652786066	1.01E-23	4.18E-22	179	179
LGALS3	0.652940242	9.81E-24	4.06E-22	179	179
SSTR1	0.653249378	9.19E-24	3.81E-22	179	179
MAPK1IP1L	0.653373692	8.96E-24	3.72E-22	179	179
FOXA1	0.65425723	7.45E-24	3.09E-22	179	179
FAM179B	0.655341361	5.93E-24	2.47E-22	179	179
SNORD58	0.655341361	5.93E-24	2.47E-22	179	179
PTGDR	0.658787391	2.86E-24	1.19E-22	179	178
PTGER2	0.658787391	2.86E-24	1.19E-22	179	178
TXNDC16	0.658787391	2.86E-24	1.19E-22	179	178
TTC6	0.65889903	2.79E-24	1.17E-22	179	179
POLE2	0.659353806	2.53E-24	1.06E-22	179	179
RNU6ATAC3	0.659353806	2.53E-24	1.06E-22	179	179
KLHDC1	0.659425085	2.49E-24	1.05E-22	179	179
KLHDC2	0.659425085	2.49E-24	1.05E-22	179	179
CGRRF1	0.661194885	1.70E-24	7.22E-23	179	179
WDHD1	0.661961792	1.45E-24	6.13E-23	179	179
SOCS4	0.663092618	1.13E-24	4.81E-23	179	179
DLGAP5	0.663615654	1.01E-24	4.30E-23	179	179
SNORA42|E	0.663756592	9.80E-25	4.18E-23	179	179
snoU83B	0.664721416	7.94E-25	3.39E-23	179	179
PYGL	0.664721416	7.94E-25	3.39E-23	179	179
ABHD12B	0.664721416	7.94E-25	3.39E-23	179	179
L2HGDH	0.66677198	5.07E-25	2.18E-23	179	179
MIR4504	0.66677198	5.07E-25	2.18E-23	179	179
LINC00519	0.666885802	4.94E-25	2.13E-23	179	179
LINC00520	0.667804714	4.03E-25	1.74E-23	179	179
RPL13AP3	0.667804714	4.03E-25	1.74E-23	179	179
ATL1	0.668421037	3.52E-25	1.52E-23	179	179
SAV1	0.668421037	3.52E-25	1.52E-23	179	179
RN7SL452P	0.668421037	3.52E-25	1.52E-23	179	179
NIN	0.668421037	3.52E-25	1.52E-23	179	179
ATP5S	0.668422001	3.52E-25	1.52E-23	179	179
CDKL1	0.668422001	3.52E-25	1.52E-23	179	179
MAP4K5	0.668422001	3.52E-25	1.52E-23	179	179

LINC00639	0.668512052	3.45E-25	1.51E-23	179	179
LINC00517	0.668847897	3.20E-25	1.41E-23	179	179
FBXO34	0.670892812	2.03E-25	8.94E-24	179	179
ATG14	0.670892812	2.03E-25	8.94E-24	179	179
TBPL2	0.670892812	2.03E-25	8.94E-24	179	179
MIR4308	0.671085318	1.95E-25	8.61E-24	179	179
MPP5	0.671488222	1.78E-25	7.88E-24	179	179
TRIM9	0.671710964	1.69E-25	7.51E-24	179	179
TMX1	0.671824302	1.65E-25	7.34E-24	179	179
FSCB	0.671964911	1.60E-25	7.12E-24	179	179
snoU13|EN	0.671964911	1.60E-25	7.12E-24	179	179
SNORA70|E	0.672030758	1.57E-25	7.04E-24	179	179
FRMD6	0.674921954	8.20E-26	3.67E-24	179	178
GPR137C	0.67586076	6.62E-26	2.97E-24	179	178
ERO1L	0.67586076	6.62E-26	2.97E-24	179	178
PSMC6	0.67586076	6.62E-26	2.97E-24	179	178
STYX	0.67586076	6.62E-26	2.97E-24	179	178
GNPNAT1	0.67586076	6.62E-26	2.97E-24	179	178
RN7SL588P	0.67586076	6.62E-26	2.97E-24	179	178
FERMT2	0.67586076	6.62E-26	2.97E-24	179	178
DDHD1	0.67586076	6.62E-26	2.97E-24	179	178
SEC23A	0.676089617	6.28E-26	2.86E-24	179	179
LINC00640	0.676364094	5.90E-26	2.69E-24	179	178
RNA5SP385	0.678399748	3.70E-26	1.69E-24	179	178
C14orf37	0.680740642	2.15E-26	9.87E-25	179	179
RN7SL598P	0.680740642	2.15E-26	9.87E-25	179	179
ACTR10	0.680740642	2.15E-26	9.87E-25	179	179
PSMA3	0.680740642	2.15E-26	9.87E-25	179	179
DNAAF2	0.681212768	1.93E-26	8.91E-25	179	179
KTN1	0.682287119	1.50E-26	6.94E-25	179	179
SNORA31|E	0.68306147	1.25E-26	5.80E-25	179	179
PRPF39	0.684771809	8.38E-27	3.89E-25	179	179
FAM71D	0.685101403	7.75E-27	3.60E-25	179	179
KIAA0586	0.685990208	6.28E-27	2.93E-25	179	179
MAX	0.686947802	5.00E-27	2.33E-25	179	179
GNG2	0.687031353	4.90E-27	2.29E-25	179	178
C14orf166	0.687031353	4.90E-27	2.29E-25	179	178
NID2	0.687031353	4.90E-27	2.29E-25	179	178
MTHFD1	0.689478521	2.73E-27	1.28E-25	179	179
ZBTB25	0.689478521	2.73E-27	1.28E-25	179	179
ATP6V1D	0.689915242	2.46E-27	1.16E-25	179	179
GEMIN2	0.690266424	2.26E-27	1.07E-25	179	179
TRAPPC6B	0.690266424	2.26E-27	1.07E-25	179	179
PNN	0.690266424	2.26E-27	1.07E-25	179	179
SNORD127	0.690569474	2.10E-27	9.98E-26	179	179
FKBP3	0.690569474	2.10E-27	9.98E-26	179	179
MIA2	0.691514769	1.67E-27	7.97E-26	179	179
CTAGE5	0.691514769	1.67E-27	7.97E-26	179	179
OTX2	0.691724275	1.59E-27	7.60E-26	179	179
RN7SL461P	0.691724275	1.59E-27	7.60E-26	179	179
MIR4708	0.691950745	1.50E-27	7.22E-26	179	179

EXOC5	0.693276028	1.09E-27	5.24E-26	179	179
AP5M1	0.693276028	1.09E-27	5.24E-26	179	179
NAA30	0.693276028	1.09E-27	5.24E-26	179	179
C14orf105	0.693276028	1.09E-27	5.24E-26	179	179
ESR2	0.695292159	6.63E-28	3.22E-26	179	179
MDGA2	0.69619927	5.30E-28	2.58E-26	179	179
FBXO33	0.697689069	3.66E-28	1.79E-26	179	179
SGPP1	0.698911609	2.70E-28	1.32E-26	179	179
U3|ENSG0C	0.699134304	2.55E-28	1.25E-26	179	179
SNORD112|	0.699337969	2.43E-28	1.19E-26	179	179
GPR135	0.699575844	2.29E-28	1.12E-26	179	179
L3HYPDH	0.699575844	2.29E-28	1.12E-26	179	179
JKAMP	0.699575844	2.29E-28	1.12E-26	179	179
CCDC175	0.699575844	2.29E-28	1.12E-26	179	179
DAAM1	0.699866738	2.12E-28	1.05E-26	179	179
DACT1	0.699932237	2.09E-28	1.04E-26	179	179
SLC35F4	0.700231463	1.94E-28	9.64E-27	179	179
RN7SKP99	0.700231463	1.94E-28	9.64E-27	179	179
ARID4A	0.700312373	1.90E-28	9.49E-27	179	179
TOMM20L	0.700312373	1.90E-28	9.49E-27	179	179
TIMM9	0.700312373	1.90E-28	9.49E-27	179	179
HIF1A	0.700640783	1.75E-28	8.79E-27	179	179
SNAPC1	0.700640783	1.75E-28	8.79E-27	179	179
EIF2S1	0.700674398	1.73E-28	8.75E-27	179	179
PLEK2	0.700674398	1.73E-28	8.75E-27	179	179
RNA5SP384	0.700732732	1.71E-28	8.66E-27	179	179
RPS29	0.700732732	1.71E-28	8.66E-27	179	179
RN7SL1	0.700732732	1.71E-28	8.66E-27	179	179
LRR1	0.700732732	1.71E-28	8.66E-27	179	179
MGAT2	0.700732732	1.71E-28	8.66E-27	179	179
RPL36AL	0.700732732	1.71E-28	8.66E-27	179	179
KCNH5	0.700948821	1.62E-28	8.30E-27	179	179
RHOJ	0.700948821	1.62E-28	8.30E-27	179	179
GPHB5	0.700948821	1.62E-28	8.30E-27	179	179
PPP2R5E	0.700948821	1.62E-28	8.30E-27	179	179
RN7SL540F	0.700948821	1.62E-28	8.30E-27	179	179
SCARNA20|	0.700948821	1.62E-28	8.30E-27	179	179
WDR89	0.700948821	1.62E-28	8.30E-27	179	179
U3|ENSG0C	0.700948821	1.62E-28	8.30E-27	179	179
MIS18BP1	0.703009556	9.60E-29	5.01E-27	179	179
MIR548H1	0.703246441	9.04E-29	4.72E-27	179	179
FANCM	0.703346685	8.81E-29	4.62E-27	179	179
AKAP5	0.704616536	6.37E-29	3.34E-27	179	179
ZBTB1	0.704616536	6.37E-29	3.34E-27	179	179
HSPA2	0.704616536	6.37E-29	3.34E-27	179	179
SYNE2	0.705268582	5.39E-29	2.85E-27	179	179
SNORD112|	0.705281586	5.37E-29	2.84E-27	179	179
TMEM30B	0.705281586	5.37E-29	2.84E-27	179	179
SYT16	0.705634125	4.91E-29	2.61E-27	179	179
LINC00643	0.705634125	4.91E-29	2.61E-27	179	179
LINC00644	0.705634125	4.91E-29	2.61E-27	179	179

PELI2	0.705803369	4.70E-29	2.51E-27	179	179
LINC00238	0.706727755	3.70E-29	1.98E-27	179	178
TMEM260	0.707120094	3.34E-29	1.80E-27	179	179
PRKCH	0.707215735	3.26E-29	1.76E-27	179	179
LINC00871	0.708024036	2.65E-29	1.43E-27	179	179
FUT8	0.708738388	2.20E-29	1.19E-27	179	179
MIR625	0.708756067	2.19E-29	1.19E-27	179	179
PLEKHH1	0.709406941	1.85E-29	1.00E-27	179	179
C14orf39	0.71008132	1.55E-29	8.42E-28	179	179
SIX6	0.71008132	1.55E-29	8.42E-28	179	179
RTN1	0.711485142	1.07E-29	5.85E-28	179	179
MIR5586	0.711485142	1.07E-29	5.85E-28	179	179
LRRC9	0.711485142	1.07E-29	5.85E-28	179	179
PCNXL4	0.711485142	1.07E-29	5.85E-28	179	179
DHRS7	0.711485142	1.07E-29	5.85E-28	179	179
PPM1A	0.711485142	1.07E-29	5.85E-28	179	179
SIX1	0.711889287	9.61E-30	5.33E-28	179	179
SIX4	0.711889287	9.61E-30	5.33E-28	179	179
MNAT1	0.711889287	9.61E-30	5.33E-28	179	179
TRMT5	0.711889287	9.61E-30	5.33E-28	179	179
SLC38A6	0.711889287	9.61E-30	5.33E-28	179	179
RPL10L	0.712508555	8.16E-30	4.57E-28	179	179
RN7SL706F	0.712566643	8.04E-30	4.51E-28	179	179
RN7SL108F	0.712566643	8.04E-30	4.51E-28	179	179
PLEKHG3	0.712641768	7.88E-30	4.45E-28	179	179
SPTB	0.712641768	7.88E-30	4.45E-28	179	179
CHURC1	0.712641768	7.88E-30	4.45E-28	179	179
GPX2	0.712641768	7.88E-30	4.45E-28	179	179
RAB15	0.712641768	7.88E-30	4.45E-28	179	179
FNTB	0.712641768	7.88E-30	4.45E-28	179	179
MIR4706	0.712641768	7.88E-30	4.45E-28	179	179
PPP1R36	0.714105248	5.34E-30	3.06E-28	179	179
MED6	0.714890272	4.33E-30	2.49E-28	179	179
RN7SL77P	0.714890272	4.33E-30	2.49E-28	179	179
ARG2	0.717675213	2.05E-30	1.18E-28	179	179
VTI1B	0.717675213	2.05E-30	1.18E-28	179	179
MIR5694	0.717829328	1.96E-30	1.14E-28	179	179
TMEM229B	0.717829328	1.96E-30	1.14E-28	179	179
RAD51B	0.719243334	1.34E-30	7.79E-29	179	179
SIPA1L1	0.719279666	1.32E-30	7.73E-29	179	179
LINC00648	0.719285935	1.32E-30	7.73E-29	179	179
MIR548Y	0.719285935	1.32E-30	7.73E-29	179	179
ADAM20P1	0.719708298	1.18E-30	6.93E-29	179	179
SLC39A9	0.72056907	9.30E-31	5.49E-29	179	179
PLEKHD1	0.72056907	9.30E-31	5.49E-29	179	179
CCDC177|E	0.72056907	9.30E-31	5.49E-29	179	179
CCDC177|E	0.72056907	9.30E-31	5.49E-29	179	179
KIAA0247	0.72056907	9.30E-31	5.49E-29	179	179
SNORD56B	0.72198535	6.30E-31	3.76E-29	179	179
RN7SL683F	0.722350411	5.70E-31	3.41E-29	179	179
ACTN1	0.722872743	4.93E-31	2.96E-29	179	179

RGS6	0.723023568	4.73E-31	2.84E-29	179	179
RDH12	0.723622476	4.01E-31	2.41E-29	179	179
ZFYVE26	0.723622476	4.01E-31	2.41E-29	179	179
SMOC1	0.723743227	3.87E-31	2.35E-29	179	179
SLC8A3	0.723991738	3.61E-31	2.20E-29	179	179
TTC9	0.724368705	3.25E-31	1.98E-29	179	179
MAP3K9	0.724368705	3.25E-31	1.98E-29	179	179
PCNX	0.724368705	3.25E-31	1.98E-29	179	179
ADAM20	0.724389547	3.24E-31	1.98E-29	179	179
ADAM21P1	0.7243934	3.23E-31	1.98E-29	179	179
COX16	0.7243934	3.23E-31	1.98E-29	179	179
SYNJ2BP	0.7243934	3.23E-31	1.98E-29	179	179
ADAM21	0.7243934	3.23E-31	1.98E-29	179	179
DCAF5	0.728490587	1.02E-31	6.34E-30	179	179
SNORA25|E	0.729538476	7.58E-32	4.72E-30	179	179
PIGH	0.731185323	4.73E-32	2.95E-30	179	179
U3|ENSG0C	0.731378319	4.47E-32	2.80E-30	179	179
RN7SL213F	0.731378319	4.47E-32	2.80E-30	179	179
NEK9	0.73312653	2.70E-32	1.70E-30	179	179
TMED10	0.73312653	2.70E-32	1.70E-30	179	179
RDH11	0.733334618	2.54E-32	1.61E-30	179	179
RN7SL369F	0.733334618	2.54E-32	1.61E-30	179	179
RN7SL356F	0.73345014	2.46E-32	1.56E-30	179	179
C14orf166	0.733703468	2.28E-32	1.45E-30	179	179
RN7SKP17	0.733703468	2.28E-32	1.45E-30	179	179
RN7SL224F	0.736270156	1.08E-32	6.91E-31	179	179
ZFP36L1	0.737983723	6.51E-33	4.18E-31	179	179
ELMSAN1	0.739286079	4.42E-33	2.84E-31	179	179
snoU13|EN	0.739286079	4.42E-33	2.84E-31	179	179
SRSF5	0.739785298	3.81E-33	2.46E-31	179	179
SLC10A1	0.739785298	3.81E-33	2.46E-31	179	179
SNORA11|E	0.739785298	3.81E-33	2.46E-31	179	179
EXD2	0.739789151	3.80E-33	2.46E-31	179	179
GALNT16	0.739789151	3.80E-33	2.46E-31	179	179
ERH	0.739789151	3.80E-33	2.46E-31	179	179
RBM25	0.74037609	3.19E-33	2.10E-31	179	179
PSEN1	0.74037609	3.19E-33	2.10E-31	179	179
IRF2BPL	0.744398135	9.46E-34	6.25E-32	179	179
KIAA1737	0.744398135	9.46E-34	6.25E-32	179	179
TMEM63C	0.744398135	9.46E-34	6.25E-32	179	179
ZDHHC22	0.744398135	9.46E-34	6.25E-32	179	179
SNORA32|E	0.744398135	9.46E-34	6.25E-32	179	179
RNU4ATAC1	0.745170649	7.47E-34	5.00E-32	179	179
FOS	0.745170649	7.47E-34	5.00E-32	179	179
VASH1	0.746051989	5.70E-34	3.84E-32	179	179
ANGEL1	0.746051989	5.70E-34	3.84E-32	179	179
DPF3	0.750463912	1.45E-34	9.79E-33	179	179
RN7SL586F	0.751095184	1.19E-34	8.05E-33	179	179
ACYP1	0.751178021	1.16E-34	7.86E-33	179	179
ZC2HC1C	0.751178021	1.16E-34	7.86E-33	179	179
snoU13|EN	0.751213298	1.14E-34	7.82E-33	179	179

DCAF4	0.751213298	1.14E-34	7.82E-33	179	179
ZFYVE1	0.751213298	1.14E-34	7.82E-33	179	179
PNMA1	0.751993269	8.93E-35	6.16E-33	179	179
MIR4505	0.751993269	8.93E-35	6.16E-33	179	179
EIF2B2	0.754239141	4.37E-35	3.03E-33	179	179
MLH3	0.754239141	4.37E-35	3.03E-33	179	179
AHSA1	0.756848758	1.89E-35	1.32E-33	179	179
SNORA46|E	0.756848758	1.89E-35	1.32E-33	179	179
ISM2	0.756848758	1.89E-35	1.32E-33	179	179
MIR1260A	0.756850684	1.89E-35	1.32E-33	179	179
NGB	0.756850684	1.89E-35	1.32E-33	179	179
POMT2	0.756850684	1.89E-35	1.32E-33	179	179
GSTZ1	0.756850684	1.89E-35	1.32E-33	179	179
TMED8	0.756850684	1.89E-35	1.32E-33	179	179
RN7SL137F	0.756850684	1.89E-35	1.32E-33	179	179
SAMD15	0.756850684	1.89E-35	1.32E-33	179	179
NOXRED1	0.756850684	1.89E-35	1.32E-33	179	179
FKSG61	0.756850684	1.89E-35	1.32E-33	179	179
VIPAS39	0.757463294	1.55E-35	1.12E-33	179	179
JDP2	0.762076284	3.40E-36	2.46E-34	179	179
BATF	0.762076284	3.40E-36	2.46E-34	179	179
TGFB3	0.762666626	2.80E-36	2.04E-34	179	179
ESRRB	0.763494998	2.12E-36	1.55E-34	179	179
RN7SL747F	0.763494998	2.12E-36	1.55E-34	179	179
FLVCR2	0.765111399	1.23E-36	9.06E-35	179	179
RNA5SP387	0.765111399	1.23E-36	9.06E-35	179	179
TTLL5	0.765111399	1.23E-36	9.06E-35	179	179
C14orf1	0.765111399	1.23E-36	9.06E-35	179	179
NPC2	0.765800698	9.76E-37	7.26E-35	179	179
MIR4709	0.765800698	9.76E-37	7.26E-35	179	179
ISCA2	0.765800698	9.76E-37	7.26E-35	179	179
LTBP2	0.765800698	9.76E-37	7.26E-35	179	179
AREL1	0.765800698	9.76E-37	7.26E-35	179	179
FCF1	0.765800698	9.76E-37	7.26E-35	179	179
SNORA7|EN	0.765800698	9.76E-37	7.26E-35	179	179
YLPM1	0.765800698	9.76E-37	7.26E-35	179	179
PROX2	0.765800698	9.76E-37	7.26E-35	179	179
DLST	0.765800698	9.76E-37	7.26E-35	179	179
RPS6KL1	0.765800698	9.76E-37	7.26E-35	179	179
PGF	0.765800698	9.76E-37	7.26E-35	179	179
PAPLN	0.765999122	9.13E-37	7.05E-35	179	179
NUMB	0.765999122	9.13E-37	7.05E-35	179	179
ACOT6	0.766788964	6.98E-37	5.42E-35	179	179
DNAL1	0.766788964	6.98E-37	5.42E-35	179	179
HEATR4	0.767287913	5.89E-37	4.60E-35	179	179
C14orf169	0.767287913	5.89E-37	4.60E-35	179	179
ACOT1	0.767287913	5.89E-37	4.60E-35	179	179
ACOT2	0.767287913	5.89E-37	4.60E-35	179	179
ACOT4	0.767287913	5.89E-37	4.60E-35	179	179
SPTLC2	0.774119349	5.51E-38	4.38E-36	179	179
RN7SL587F	0.774119349	5.51E-38	4.38E-36	179	179

ALKBH1	0.774119349	5.51E-38	4.38E-36	179	179
SLIRP	0.774119349	5.51E-38	4.38E-36	179	179
SNW1	0.774119349	5.51E-38	4.38E-36	179	179
C14orf178	0.774119349	5.51E-38	4.38E-36	179	179
ADCK1	0.774123202	5.51E-38	4.38E-36	179	179
IFT43	0.776785551	2.14E-38	1.74E-36	179	179
GPATCH2L	0.776787477	2.14E-38	1.74E-36	179	179
COQ6	0.778187736	1.29E-38	1.06E-36	179	179
ENTPD5	0.778187736	1.29E-38	1.06E-36	179	179
CCDC176	0.778187736	1.29E-38	1.06E-36	179	179
ALDH6A1	0.778187736	1.29E-38	1.06E-36	179	179
LIN52	0.778187736	1.29E-38	1.06E-36	179	179
RN7SL530P	0.778187736	1.29E-38	1.06E-36	179	179
VSX2	0.778187736	1.29E-38	1.06E-36	179	179
ABCD4	0.778187736	1.29E-38	1.06E-36	179	179
VRTN	0.778187736	1.29E-38	1.06E-36	179	179
SYNDIG1L	0.778187736	1.29E-38	1.06E-36	179	179
ZNF410	0.778741964	1.06E-38	8.95E-37	179	179
FAM161B	0.778741964	1.06E-38	8.95E-37	179	179
PTGR2	0.778791676	1.04E-38	8.85E-37	179	179
NRXN3	0.786006314	7.28E-40	6.22E-38	179	179
RNA5SP388	0.796333202	1.35E-41	1.16E-39	179	179
CEP128	0.800211234	2.84E-42	2.44E-40	179	179
AKT1	0.871015748	4.45E-58	3.84E-56	179	179
ZBTB42	0.871015748	4.45E-58	3.84E-56	179	179
LINC00638	0.871015748	4.45E-58	3.84E-56	179	179
MIR4710	0.880647979	6.02E-61	5.25E-59	179	179
GTF2A1	0.881752372	2.72E-61	2.38E-59	179	179
TMEM179	0.882568647	1.51E-61	1.32E-59	179	179
C14orf180	0.882568647	1.51E-61	1.32E-59	179	179
SNORA11B	0.882893309	1.19E-61	1.05E-59	179	179
RN7SL506P	0.882893309	1.19E-61	1.05E-59	179	179
INF2	0.883117684	1.01E-61	8.99E-60	179	179
SNORA79|E	0.883647994	6.84E-62	6.11E-60	179	179
STON2	0.883647994	6.84E-62	6.11E-60	179	179
SEL1L	0.883647994	6.84E-62	6.11E-60	179	179
FLRT2	0.883825988	6.00E-62	5.42E-60	179	179
snoU13|EN	0.883956225	5.45E-62	4.94E-60	179	179
ADSSL1	0.884302448	4.22E-62	3.84E-60	179	179
SIVA1	0.884947808	2.61E-62	2.39E-60	179	179
DIO2	0.887680634	3.34E-63	3.06E-61	179	179
TSHR	0.889649835	7.32E-64	6.74E-62	179	179
RNU6ATAC2	0.890863601	2.83E-64	2.62E-62	179	179
ZFYVE21	0.891254993	2.08E-64	1.93E-62	179	179
RNU3P3	0.892902741	5.60E-65	5.22E-63	179	179
LINC00911	0.892992321	5.21E-65	4.87E-63	179	179
FOXN3	0.895738265	5.57E-66	5.23E-64	179	179
FBLN5	0.898117044	7.62E-67	7.18E-65	179	179
RPS6KA5	0.902384121	1.90E-68	1.79E-66	179	179
C14orf159	0.902402422	1.87E-68	1.77E-66	179	179
KCNK10	0.907086574	2.64E-70	2.52E-68	179	179

JAG2	0.908860349	4.97E-71	4.75E-69	179	179
NUDT14	0.908860349	4.97E-71	4.75E-69	179	179
BRF1	0.909212888	3.55E-71	3.42E-69	179	179
CEP170B	0.909490734	2.72E-71	2.63E-69	179	179
PLD4	0.909490734	2.72E-71	2.63E-69	179	179
AHNAK2	0.909490734	2.72E-71	2.63E-69	179	179
C14orf79	0.909490734	2.72E-71	2.63E-69	179	179
RIN3	0.910480008	1.05E-71	1.03E-69	179	179
LGMN	0.910480008	1.05E-71	1.03E-69	179	179
GOLGA5	0.910480008	1.05E-71	1.03E-69	179	179
PPP1R13B	0.910540648	9.88E-72	9.83E-70	179	179
GPR132	0.912670334	1.22E-72	1.22E-70	179	179
CDCA4	0.91281867	1.05E-72	1.05E-70	179	179
SPATA7	0.916274755	3.11E-74	3.13E-72	179	179
PTPN21	0.916274755	3.11E-74	3.13E-72	179	179
GALC	0.917334261	1.02E-74	1.04E-72	179	179
GPR65	0.917334261	1.02E-74	1.04E-72	179	179
GPR68	0.91742099	9.35E-75	9.57E-73	179	179
CCDC88C	0.91742099	9.35E-75	9.57E-73	179	179
EML5	0.917535574	8.28E-75	8.55E-73	179	179
TTC8	0.91862498	2.60E-75	2.69E-73	179	179
TDRD9	0.921048449	1.86E-76	1.93E-74	179	179
SNORD51|E	0.921125507	1.70E-76	1.78E-74	179	179
TTC7B	0.921516094	1.10E-76	1.16E-74	179	179
SMEK1	0.921660134	9.40E-77	9.91E-75	179	179
CATSPERB	0.921660134	9.40E-77	9.91E-75	179	179
CDC42BPB	0.921848368	7.62E-77	8.10E-75	179	179
RN7SL634F	0.921951953	6.79E-77	7.25E-75	179	179
ASPG	0.921951953	6.79E-77	7.25E-75	179	179
C14orf2	0.923298539	1.48E-77	1.60E-75	179	179
U3|ENSG0C	0.923390082	1.34E-77	1.44E-75	179	179
TC2N	0.923390082	1.34E-77	1.44E-75	179	179
TRIP11	0.923558646	1.10E-77	1.20E-75	179	179
ATXN3	0.923558646	1.10E-77	1.20E-75	179	179
NDUFB1	0.923558646	1.10E-77	1.20E-75	179	179
MIR203	0.924134616	5.68E-78	6.29E-76	179	179
KIF26A	0.924134616	5.68E-78	6.29E-76	179	179
C14orf144	0.924134616	5.68E-78	6.29E-76	179	179
RD3L	0.924202042	5.26E-78	5.89E-76	179	179
CHGA	0.925082464	1.89E-78	2.13E-76	179	179
FAM181A	0.92664729	2.98E-79	3.37E-77	179	179
LINC00637	0.926656334	2.95E-79	3.35E-77	179	179
PSMC1	0.926962569	2.04E-79	2.33E-77	179	179
NRDE2	0.927035328	1.87E-79	2.15E-77	179	179
ZC3H14	0.927993701	5.87E-80	6.77E-78	179	179
BTBD6	0.928555363	2.95E-80	3.42E-78	179	179
MOAP1	0.929121257	1.47E-80	1.71E-78	179	179
TMEM251	0.929121257	1.47E-80	1.71E-78	179	179
C14orf142	0.929121257	1.47E-80	1.71E-78	179	179
UBR7	0.929121257	1.47E-80	1.71E-78	179	179
BTBD7	0.929121257	1.47E-80	1.71E-78	179	179

ITPK1	0.92929849	1.18E-80	1.41E-78	179	179
PRIMA1	0.929942438	5.29E-81	6.33E-79	179	179
ASB2	0.929943435	5.28E-81	6.33E-79	179	179
MIR4506	0.929943435	5.28E-81	6.33E-79	179	179
AMN	0.930063703	4.54E-81	5.52E-79	179	179
TRAF3	0.930277538	3.47E-81	4.24E-79	179	179
CALM1	0.935910501	2.11E-84	2.59E-82	179	179
LINC00642	0.935910501	2.11E-84	2.59E-82	179	179
TEX22	0.93758365	2.05E-85	2.54E-83	179	179
MTA1	0.93758365	2.05E-85	2.54E-83	179	179
CRIP2	0.937732949	1.66E-85	2.08E-83	179	179
C14orf80	0.937732949	1.66E-85	2.08E-83	179	179
CRIP1	0.937732949	1.66E-85	2.08E-83	179	179
ADAM6	0.937732949	1.66E-85	2.08E-83	179	179
IGHA1	0.937732949	1.66E-85	2.08E-83	179	179
IGHA2	0.937732949	1.66E-85	2.08E-83	179	179
IGHD	0.937732949	1.66E-85	2.08E-83	179	179
IGHE	0.937732949	1.66E-85	2.08E-83	179	179
IGHG1	0.937732949	1.66E-85	2.08E-83	179	179
IGHG2	0.937732949	1.66E-85	2.08E-83	179	179
IGHG3	0.937732949	1.66E-85	2.08E-83	179	179
IGHG4	0.937732949	1.66E-85	2.08E-83	179	179
IGHJ1	0.937732949	1.66E-85	2.08E-83	179	179
IGHJ2	0.937732949	1.66E-85	2.08E-83	179	179
IGHJ3	0.937732949	1.66E-85	2.08E-83	179	179
IGHJ4	0.937732949	1.66E-85	2.08E-83	179	179
IGHJ5	0.937732949	1.66E-85	2.08E-83	179	179
IGHJ6	0.937732949	1.66E-85	2.08E-83	179	179
IGHM	0.937732949	1.66E-85	2.08E-83	179	179
KIAA0125	0.937732949	1.66E-85	2.08E-83	179	179
LINC00221	0.937732949	1.66E-85	2.08E-83	179	179
LINC0022€	0.937732949	1.66E-85	2.08E-83	179	179
RNA5SP389	0.937732949	1.66E-85	2.08E-83	179	179
TMEM121	0.937732949	1.66E-85	2.08E-83	179	179
UNC79	0.938753484	3.87E-86	5.51E-84	179	179
COX8C	0.938753484	3.87E-86	5.51E-84	179	179
MARK3	0.938959073	2.87E-86	4.14E-84	179	179
XRCC3	0.939245661	1.90E-86	2.75E-84	179	179
snoU13|EN	0.939575989	1.17E-86	1.71E-84	179	179
EIF5	0.940107236	5.38E-87	7.89E-85	179	179
SNORA28	0.940107236	5.38E-87	7.89E-85	179	179
PACS2	0.94188155	3.78E-88	5.61E-86	179	179
ZNF839	0.943452993	3.36E-89	5.01E-87	179	179
CINP	0.943452993	3.36E-89	5.01E-87	179	179
snoU13|EN	0.943452993	3.36E-89	5.01E-87	179	179
TECPR2	0.943452993	3.36E-89	5.01E-87	179	179
MOK	0.943574359	2.78E-89	4.24E-87	179	179
RN7SKP107	0.94454577	5.97E-90	9.19E-88	179	179
U3|ENSG0C	0.946033469	5.38E-91	8.34E-89	179	179
LINC00524	0.946033469	5.38E-91	8.34E-89	179	179
DIO3OS	0.946033469	5.38E-91	8.34E-89	179	179

DIO3	0.946033469	5.38E-91	8.34E-89	179	179
PPP2R5C	0.946033469	5.38E-91	8.34E-89	179	179
EXOC3L4	0.946800677	1.52E-91	2.42E-89	179	179
LINC00677	0.946800677	1.52E-91	2.42E-89	179	179
TNFAIP2	0.946800677	1.52E-91	2.42E-89	179	179
LINC00605	0.946800677	1.52E-91	2.42E-89	179	179
TDP1	0.947059784	9.83E-92	1.61E-89	179	179
CPSF2	0.948945343	3.95E-93	6.53E-91	179	179
HSP90AA1	0.950099236	5.20E-94	8.65E-92	179	179
WDR20	0.950099236	5.20E-94	8.65E-92	179	179
SLC24A4	0.950189827	4.43E-94	7.46E-92	179	179
MIR4309	0.950269245	3.84E-94	6.52E-92	179	179
ANKRD9	0.950808169	1.46E-94	2.50E-92	179	179
TCL6	0.951047119	9.49E-95	1.63E-92	179	179
RCOR1	0.951139999	8.01E-95	1.39E-92	179	179
RN7SKP255	0.952455763	7.10E-96	1.24E-93	179	179
EFCAB11	0.952457689	7.07E-96	1.24E-93	179	179
KCNK13	0.952547728	5.97E-96	1.06E-93	179	179
CKB	0.953698825	6.74E-97	1.20E-94	179	179
TRMT61A	0.953698825	6.74E-97	1.20E-94	179	179
BAG5	0.953698825	6.74E-97	1.20E-94	179	179
KLC1	0.953698825	6.74E-97	1.20E-94	179	179
APOPT1	0.953698825	6.74E-97	1.20E-94	179	179
CLMN	0.955951847	7.97E-99	1.47E-96	179	179
LINC00341	0.955951847	7.97E-99	1.47E-96	179	179
SYNE3	0.955951847	7.97E-99	1.47E-96	179	179
SNHG10	0.955951847	7.97E-99	1.47E-96	179	179
SCARNA13	0.955951847	7.97E-99	1.47E-96	179	179
GLRX5	0.955951847	7.97E-99	1.47E-96	179	179
TCL1B	0.955957626	7.88E-99	1.47E-96	179	179
TCL1A	0.955957626	7.88E-99	1.47E-96	179	179
LINC00521	0.956551473	2.35E-99	4.63E-97	179	179
PPP4R4	0.959957926	1.63E-102	3.23E-100	179	179
SERPINA10	0.959957926	1.63E-102	3.23E-100	179	179
SERPINA6	0.959957926	1.63E-102	3.23E-100	179	179
RN7SL472P	0.961634802	3.59E-104	7.28E-102	179	179
DYNC1H1	0.961640582	3.54E-104	7.25E-102	179	179
SERPINA1	0.962943413	1.62E-105	3.34E-103	179	179
SERPINA11	0.962943413	1.62E-105	3.34E-103	179	179
SERPINA9	0.962943413	1.62E-105	3.34E-103	179	179
SERPINA12	0.962943413	1.62E-105	3.34E-103	179	179
SERPINA4	0.962943413	1.62E-105	3.34E-103	179	179
SERPINA5	0.962943413	1.62E-105	3.34E-103	179	179
SERPINA3	0.962943413	1.62E-105	3.34E-103	179	179
SERPINA13	0.962943413	1.62E-105	3.34E-103	179	179
GSC	0.96297759	1.49E-105	3.30E-103	179	179
OTUB2	0.965363026	3.87E-108	8.64E-106	179	179
DDX24	0.965363026	3.87E-108	8.64E-106	179	179
IFI27L1	0.965363026	3.87E-108	8.64E-106	179	179
IFI27	0.965363026	3.87E-108	8.64E-106	179	179
IFI27L2	0.965363026	3.87E-108	8.64E-106	179	179

LINC00523	0.967627017	9.14E-111	2.14E-108	179	179
DLK1	0.967627017	9.14E-111	2.14E-108	179	179
MEG3	0.967627017	9.14E-111	2.14E-108	179	179
MIR2392	0.967627017	9.14E-111	2.14E-108	179	179
MIR770	0.967627017	9.14E-111	2.14E-108	179	179
MIR493	0.967627017	9.14E-111	2.14E-108	179	179
MIR337	0.967627017	9.14E-111	2.14E-108	179	179
MIR431	0.967627017	9.14E-111	2.14E-108	179	179
MIR433	0.967627017	9.14E-111	2.14E-108	179	179
MIR665	0.967627017	9.14E-111	2.14E-108	179	179
RTL1	0.967627017	9.14E-111	2.14E-108	179	179
MIR127	0.967627017	9.14E-111	2.14E-108	179	179
MIR136	0.967627017	9.14E-111	2.14E-108	179	179
MIR432	0.967627017	9.14E-111	2.14E-108	179	179
MEG8	0.967627017	9.14E-111	2.14E-108	179	179
MIR370	0.967627017	9.14E-111	2.14E-108	179	179
SNORD112|	0.967627017	9.14E-111	2.14E-108	179	179
SNORD112|	0.967627017	9.14E-111	2.14E-108	179	179
SNORD112|	0.967627017	9.14E-111	2.14E-108	179	179
SNORD112|	0.967627017	9.14E-111	2.14E-108	179	179
SNORD112|	0.967627017	9.14E-111	2.14E-108	179	179
SNORD113|	0.967627017	9.14E-111	2.14E-108	179	179
SNORD113|	0.967627017	9.14E-111	2.14E-108	179	179
SNORD113|	0.967627017	9.14E-111	2.14E-108	179	179
SNORD113|	0.967627017	9.14E-111	2.14E-108	179	179
SNORD113|	0.967627017	9.14E-111	2.14E-108	179	179
SNORD112|	0.967627017	9.14E-111	2.14E-108	179	179
SNORD113|	0.967627017	9.14E-111	2.14E-108	179	179
MIR379	0.967627017	9.14E-111	2.14E-108	179	179
MIR411	0.967627017	9.14E-111	2.14E-108	179	179
MIR299	0.967627017	9.14E-111	2.14E-108	179	179
MIR380	0.967627017	9.14E-111	2.14E-108	179	179
MIR1197	0.967627017	9.14E-111	2.14E-108	179	179
MIR323A	0.967627017	9.14E-111	2.14E-108	179	179
MIR758	0.967627017	9.14E-111	2.14E-108	179	179
MIR1193	0.967627017	9.14E-111	2.14E-108	179	179
MIR494	0.967627017	9.14E-111	2.14E-108	179	179
MIR495	0.967627017	9.14E-111	2.14E-108	179	179
MIR543	0.967627017	9.14E-111	2.14E-108	179	179
MIR300	0.967627017	9.14E-111	2.14E-108	179	179
MIR376A1	0.967627017	9.14E-111	2.14E-108	179	179
MIR376C	0.967627017	9.14E-111	2.14E-108	179	179
MIR654	0.967627017	9.14E-111	2.14E-108	179	179
MIR381HG	0.967627017	9.14E-111	2.14E-108	179	179
MIR381	0.967627017	9.14E-111	2.14E-108	179	179
MIR487B	0.967627017	9.14E-111	2.14E-108	179	179
MIR539	0.967627017	9.14E-111	2.14E-108	179	179
MIR889	0.967627017	9.14E-111	2.14E-108	179	179
MIR544A	0.967627017	9.14E-111	2.14E-108	179	179
MIR655	0.967627017	9.14E-111	2.14E-108	179	179
MIR487A	0.967627017	9.14E-111	2.14E-108	179	179

MIR134	0.967627017	9.14E-111	2.14E-108	179	179
MIR382	0.967627017	9.14E-111	2.14E-108	179	179
MIR485	0.967627017	9.14E-111	2.14E-108	179	179
MIR323B	0.967627017	9.14E-111	2.14E-108	179	179
MIR154	0.967627017	9.14E-111	2.14E-108	179	179
MIR377	0.967627017	9.14E-111	2.14E-108	179	179
MIR496	0.967627017	9.14E-111	2.14E-108	179	179
MIR369	0.967627017	9.14E-111	2.14E-108	179	179
MIR409	0.967627017	9.14E-111	2.14E-108	179	179
MIR410	0.967627017	9.14E-111	2.14E-108	179	179
MIR412	0.967627017	9.14E-111	2.14E-108	179	179
MIR541	0.967627017	9.14E-111	2.14E-108	179	179
MIR656	0.967627017	9.14E-111	2.14E-108	179	179
MEG9	0.967627017	9.14E-111	2.14E-108	179	179
RN7SL546F	0.968219881	1.75E-111	1.06E-108	179	179
LINC00617	0.968316731	1.33E-111	8.23E-109	179	179
C14orf132	0.968316731	1.33E-111	8.23E-109	179	179
DICER1	0.968484331	8.26E-112	5.39E-109	179	179
MIR3173	0.968484331	8.26E-112	5.39E-109	179	179
BDKRB2	0.968589262	6.13E-112	4.22E-109	179	179
BDKRB1	0.968589262	6.13E-112	4.22E-109	179	179
DKFZP434C	0.968589262	6.13E-112	4.22E-109	179	179
ATG2B	0.968589262	6.13E-112	4.22E-109	179	179
snoU13|EN	0.968589262	6.13E-112	4.22E-109	179	179
GSKIP	0.968595041	6.03E-112	4.22E-109	179	179
AK7	0.968595041	6.03E-112	4.22E-109	179	179
PAPOLA	0.968595041	6.03E-112	4.22E-109	179	179
RN7SKP108	0.968595041	6.03E-112	4.22E-109	179	179
VRK1	0.968800705	3.35E-112	3.07E-109	179	179
LINC00618	0.968800705	3.35E-112	3.07E-109	179	179
RN7SKP92	0.968996239	1.90E-112	1.89E-109	179	179
BEGAIN	0.968996239	1.90E-112	1.89E-109	179	179
RN7SL523F	0.993041191	5.02E-171	5.41E-168	179	179
SLC25A47	0.993041191	5.02E-171	5.41E-168	179	179
WARS	0.993041191	5.02E-171	5.41E-168	179	179
SETD3	0.994374298	2.10E-179	2.60E-176	179	179
U6|ENSG0C	0.994374298	2.10E-179	2.60E-176	179	179
CCNK	0.994374298	2.10E-179	2.60E-176	179	179
EML1	0.995468514	6.24E-188	9.10E-185	179	179
RN7SL710F	0.99618564	1.00E-194	1.55E-191	179	179
C14orf64	0.99618564	1.00E-194	1.55E-191	179	179
CYP46A1	0.996192379	8.53E-195	1.51E-191	179	179
RN7SL714F	0.996387917	7.10E-197	1.35E-193	179	179
C14orf177	0.996387917	7.10E-197	1.35E-193	179	179
BCL11B	0.996390323	6.68E-197	1.35E-193	179	179
CCDC85C	0.996915279	4.21E-203	1.04E-199	179	179
HHIPL1	0.996915279	4.21E-203	1.04E-199	179	179
EVL	0.99708673	2.33E-205	7.22E-202	179	179
MIR151B	0.99708673	2.33E-205	7.22E-202	179	179
MIR342	0.99708673	2.33E-205	7.22E-202	179	179
DEGS2	0.99708673	2.33E-205	7.22E-202	179	179

WDR25	0.999349342	1.47E-264	9.11E-261	179	179
YY1	1	1.00E-264	1.00E-261	179	179
SLC25A29	1	1.00E-264	1.00E-261	179	179
MIR345	1	1.00E-264	1.00E-261	179	179
