# Supplementary material for: Upregulation of Yin-Yang-1 Associates with Proliferation and Glutamine Metabolism in Esophageal Carcinoma
Source: Int J Genomics. 2022 Mar 20;2022:9305081. doi: 10.1155/2022/9305081 (PMC8961439; doi:10.1155/2022/9305081)
Supplement: Supplementary 4 — Table S3: functional enrichment analysis of YY1-related genes. The table consists of four parts, which are composed of biological processes (BP), cellular components (CC), molecular functions (MF), and Kyoto Encyclopedia of Genes and Genomes (KEGG). We screened out the items with statistical significance according to p < 0.01. It includes 22 biological processes, 15 cellular components, 21 molecular functions, and 9 KEGG signaling pathways. These items are used to draw bubble charts. [file 9305081.f4.pdf]

BP

Category	Term	Count	%	PValue	Genes	List Total	Pop Hits
GOTERM_BP_	GO:0050871	16	0.813421454	5.33E-11	IGHM, IGLC	1279	26
GOTERM_BP_	GO:000691(	16	0.813421454	2.23E-10	IGHM, IGLC	1279	28
GOTERM_BP_	GO:0006911	17	0.864260295	1.17E-09	IGHM, IGLC	1279	35
GOTERM_BP_	GO:0050853	19	0.965937977	4.50E-08	IGHM, IGLC	1279	54
GOTERM_BP_	GO:0010529	7	0.355871886	4.71E-06	APOBEC3C,	1279	8
GOTERM_BP_	GO:0007131	10	0.508388409	2.75E-04	RAD51B, SY	1279	30
GOTERM_BP_	GO:0048704	11	0.55922725	5.19E-04	OSR2, GSC,	1279	39
GOTERM_BP_	GO:0070383	5	0.254194204	9.69E-04	APOBEC3D,	1279	7
GOTERM_BP_	GO:0060122	7	0.355871886	0.002195963	CECR2, TTC	1279	19
GOTERM_BP_	GO:0090502	12	0.61006609	0.002526318	POP1, APEX	1279	55
GOTERM_BP_	GO:0006749	12	0.61006609	0.002931986	GGTLC2, GS	1279	56
GOTERM_BP_	GO:0009972	5	0.254194204	0.00308283	APOBEC3C,	1279	9
GOTERM_BP_	GO:0046474	5	0.254194204	0.00308283	CHKB, CECR	1279	9
GOTERM_BP_	GO:0060071	16	0.813421454	0.003869335	PSMB11, FZ	1279	92
GOTERM_BP_	GO:0048538	10	0.508388409	0.004425227	TBX1, BCL1	1279	43
GOTERM_BP_	GO:0050907	16	0.813421454	0.0052677	OR4D2, OR4	1279	95
GOTERM_BP_	GO:0042157	9	0.457549568	0.006883008	APOL5, APC	1279	38
GOTERM_BP_	GO:0009952	14	0.711743772	0.007096495	TBX1, CELS	1279	80
GOTERM_BP_	GO:003597(	5	0.254194204	0.007137311	PDP1, PPM1	1279	11
GOTERM_BP_	GO:0006958	16	0.813421454	0.007739871	IGHM, IGLC	1279	99
GOTERM_BP_	GO:0006487	9	0.457549568	0.00809466	FUT8, GAL3	1279	39
GOTERM_BP_	GO:0007141	6	0.305033045	0.009393676	RAD51C, MF	1279	18
GOTERM_BP_	GO:0071712	5	0.254194204	0.010068549	POMT2, UFI	1279	12
GOTERM_BP_	GO:0043488	16	0.813421454	0.01105944	PSMB11, SA	1279	103
GOTERM_BP_	GO:0042148	4	0.203355363	0.01219463	RAD51B, RA	1279	7
GOTERM_BP_	GO:0045869	4	0.203355363	0.01219463	APOBEC3D,	1279	7
GOTERM_BP_	GO:001937(	6	0.305033045	0.014973271	GGTLC2, GC	1279	20
GOTERM_BP_	GO:0071375	6	0.305033045	0.014973271	XBP1, CYP1	1279	20
GOTERM_BP_	GO:0008218	3	0.152516523	0.016484792	SERPIND1,	1279	3
GOTERM_BP_	GO:0006397	23	1.16929334	0.017450804	SF3A1, RBM	1279	179
GOTERM_BP_	GO:0072089	4	0.203355363	0.018418142	RNF43, ZNF	1279	8
GOTERM_BP_	GO:0019835	6	0.305033045	0.018449021	GSDMD, TST	1279	21
GOTERM_BP_	GO:0097421	7	0.355871886	0.020019315	NFKBIA, PF	1279	29
GOTERM_BP_	GO:0016192	20	1.016776817	0.022065592	NSF, RAB2F	1279	152
GOTERM_BP_	GO:0016973	5	0.254194204	0.023121236	ENY2, PABF	1279	15
GOTERM_BP_	GO:190074(	7	0.355871886	0.023459306	PPP1R13B,	1279	30
GOTERM_BP_	GO:0006605	8	0.406710727	0.026083448	SYNGR1, TC	1279	39
GOTERM_BP_	GO:0003094	4	0.203355363	0.026085798	XPNPEP3, I	1279	9
GOTERM_BP_	GO:0042742	19	0.965937977	0.026990088	IGLC7, CEF	1279	145
GOTERM_BP_	GO:0048701	7	0.355871886	0.02727404	TBX1, BMP4	1279	31
GOTERM_BP_	GO:0001701	23	1.16929334	0.027311653	JAG2, MBNI	1279	187
GOTERM_BP_	GO:0006869	12	0.61006609	0.028293593	APOL5, APC	1279	76
GOTERM_BP_	GO:0080111	5	0.254194204	0.02901506	APOBEC3C,	1279	16
GOTERM_BP_	GO:000675(	5	0.254194204	0.02901506	GGT5, GGT3	1279	16
GOTERM_BP_	GO:000652(	8	0.406710727	0.029609759	SLC7A4, GC	1279	40

GOTERM_BP_GO:001608(	3	0.152516523	0.031310249	SCRIB, PSI	1279	4
GOTERM_BP_GO:004816	7	0.355871886	0.031477201	JPH4, SIP/	1279	32
GOTERM_BP_GO:003112	9	0.457549568	0.03384966	CPSF1, PAI	1279	50
GOTERM_BP_GO:0090501	4	0.203355363	0.035195272	RNASE7, RN	1279	10
GOTERM_BP_GO:007028	4	0.203355363	0.035195272	CCDC39, DN	1279	10
GOTERM_BP_GO:003087	6	0.305033045	0.037469602	TBX1, TG,	1279	25
GOTERM_BP_GO:0006521	9	0.457549568	0.03757201	PSMB11, PS	1279	51
GOTERM_BP_GO:003017	9	0.457549568	0.041559243	RNF43, ZNI	1279	52
GOTERM_BP_GO:000016	29	1.474326385	0.042925268	PSMB11, PI	1279	262
GOTERM_BP_GO:002190	4	0.203355363	0.045717007	FOXA1, BMI	1279	11
GOTERM_BP_GO:000717	13	0.660904931	0.045941366	KLF10, TGI	1279	92
GOTERM_BP_GO:005091	7	0.355871886	0.046526316	BMP4, LGAI	1279	35
GOTERM_BP_GO:003464	8	0.406710727	0.046874108	YY1, MYC,	1279	44
GOTERM_BP_GO:006007(	12	0.61006609	0.049462636	STK11, HO)	1279	83
GOTERM_BP_GO:004455(	3	0.152516523	0.049575079	CYP11B2, (	1279	5
GOTERM_BP_GO:007258	3	0.152516523	0.049575079	PACSIN2, M	1279	5
GOTERM_BP_GO:004504(	3	0.152516523	0.049575079	HSP90AA1,	1279	5

Pop Total	Fold Enrichm	Bonferroni	Benjamini	FDR
16792	8.079388946	1.94E-07	1.94E-07	1.94E-07
16792	7.502289735	8.12E-07	4.06E-07	4.06E-07
16792	6.376946275	4.26E-06	1.42E-06	1.42E-06
16792	4.619465439	1.64E-04	4.09E-05	4.09E-05
16792	11.48788116	0.016958944	0.003420871	0.003418047
16792	4.376335679	0.631844342	0.16651868	0.166381212
16792	3.703053267	0.848461872	0.269489745	0.269267272
16792	9.377862169	0.970537499	0.440366033	0.440002494
16792	4.837002592	0.999660795	0.861769511	0.861058089
16792	2.864510626	0.999898175	0.861769511	0.861058089
16792	2.813358651	0.99997678	0.861769511	0.861058089
16792	7.293892798	0.999986601	0.861769511	0.861058089
16792	7.293892798	0.999986601	0.861769511	0.861058089
16792	2.283305572	0.999999239	1	0.999449491
16792	3.05325745	0.9999999	1	0.999449491
16792	2.211201185	0.999999995	1	0.999449491
16792	3.109501667	1	1	0.999449491
16792	2.297576231	1	1	0.999449491
16792	5.967730471	1	1	0.999449491
16792	2.121859723	1	1	0.999449491
16792	3.029770855	1	1	0.999449491
16792	4.376335679	1	1	0.999449491
16792	5.470419599	1	1	0.999449491
16792	2.039457404	1	1	0.999449491
16792	7.502289735	1	1	0.999449491
16792	7.502289735	1	1	0.999449491
16792	3.938702111	1	1	0.999449491
16792	3.938702111	1	1	0.999449491
16792	13.12900704	1	1	0.999449491
16792	1.686967385	1	1	0.999449491
16792	6.564503518	1	1	0.999449491
16792	3.751144868	1	1	0.999449491
16792	3.169070664	1	1	0.999449491
16792	1.727500926	1	1	0.999449491
16792	4.376335679	1	1	0.999449491
16792	3.063434975	1	1	0.999449491
16792	2.693129649	1	1	0.999449491
16792	5.835114239	1	1	0.999449491
16792	1.720352646	1	1	0.999449491
16792	2.964614492	1	1	0.999449491
16792	1.614797657	1	1	0.999449491
16792	2.073001111	1	1	0.999449491
16792	4.102814699	1	1	0.999449491
16792	4.102814699	1	1	0.999449491
16792	2.625801407	1	1	0.999449491

16792	9.846755278	1	1	0.999449491
16792	2.871970289	1	1	0.999449491
16792	2.363221267	1	1	0.999449491
16792	5.251602815	1	1	0.999449491
16792	5.251602815	1	1	0.999449491
16792	3.150961689	1	1	0.999449491
16792	2.316883595	1	1	0.999449491
16792	2.272328141	1	1	0.999449491
16792	1.453210703	1	1	0.999449491
16792	4.774184377	1	1	0.999449491
16792	1.855185777	1	1	0.999449491
16792	2.625801407	1	1	0.999449491
16792	2.387092188	1	1	0.999449491
16792	1.898169692	1	1	0.999449491
16792	7.877404222	1	1	0.999449491
16792	7.877404222	1	1	0.999449491
16792	7.877404222	1	1	0.999449491

CC

Category	Term	Count	%	PValue	Genes	List Total	Pop Hits
GOTERM_CC_	GO:004257[illegible]	13	0.660904931	9.06E-10	IGLC7, IG[illegible]	1365	19
GOTERM_CC_	GO:000573[illegible]	131	6.659888155	9.37E-04	ISCA2, CE[illegible]	1365	1331
GOTERM_CC_	GO:000565[illegible]	246	12.50635486	0.00285583	CCNK, SMA[illegible]	1365	2784
GOTERM_CC_	GO:003136[illegible]	7	0.355871886	0.004568994	GGTLC2, G[illegible]	1365	22
GOTERM_CC_	GO:007256[illegible]	21	1.067615658	0.009623987	SERPINA3,	1365	152
GOTERM_CC_	GO:000562[illegible]	121	6.151499746	0.018028366	GMFB, PIT[illegible]	1365	1332
GOTERM_CC_	GO:003329[illegible]	5	0.254194204	0.021878177	EIF3L, EI[illegible]	1365	15
GOTERM_CC_	GO:000573[illegible]	423	21.50482969	0.027974768	RPL30, JR[illegible]	1365	5222
GOTERM_CC_	GO:000557[illegible]	30	1.525165226	0.028908731	SERPINA1,	1365	268
GOTERM_CC_	GO:000093[illegible]	12	0.61006609	0.030186221	APOBEC3D,	1365	78
GOTERM_CC_	GO:003101[illegible]	32	1.626842908	0.036425051	RPL30, CO[illegible]	1365	296
GOTERM_CC_	GO:003651[illegible]	4	0.203355363	0.043823614	RNF139, S[illegible]	1365	11
GOTERM_CC_	GO:000574[illegible]	44	2.236908998	0.045457185	ATP5S, SL[illegible]	1365	441
GOTERM_CC_	GO:003306[illegible]	3	0.152516523	0.048072565	RAD51B, R[illegible]	1365	5
GOTERM_CC_	GO:003125[illegible]	5	0.254194204	0.048997786	ITGB3, PD[illegible]	1365	19

Pop Total	Fold Enrichm	Bonferroni	Benjamini	FDR
18224	9.134837093	6.30E-07	6.30E-07	6.30E-07
18224	1.31402702	0.478718679	0.325580093	0.325580093
18224	1.179714538	0.862981815	0.661600695	0.661600695
18224	4.248018648	0.958527595	0.793862636	0.793862636
18224	1.844534413	0.998794746	1	1
18224	1.212808413	0.999996773	1	1
18224	4.45030525	0.99999979	1	1
18224	1.081470196	0.999999997	1	1
18224	1.494505495	0.999999999	1	1
18224	2.053987039	0.999999999	1	1
18224	1.443342243	1	1	1
18224	4.854878455	1	1	1
18224	1.332064157	1	1	1
18224	8.010549451	1	1	1
18224	3.513398882	1	1	1

MF

Category	Term	Count	%	PValue	Genes	List Total	Pop Hits
GOTERM_MF_	GO:0034987	16	0.813421454	5.00E-11	IGHM, IGLC	1280	26
GOTERM_MF_	GO:0004540	11	0.55922725	2.76E-06	RNASE11, R	1280	23
GOTERM_MF_	GO:0004519	15	0.762582613	2.19E-05	RNASE11, R	1280	52
GOTERM_MF_	GO:0016814	7	0.355871886	6.19E-05	APOBEC3C,	1280	11
GOTERM_MF_	GO:0003840	6	0.305033045	4.52E-04	GGTLC2, GG	1280	10
GOTERM_MF_	GO:0004522	4	0.203355363	0.001637511	RNASE8, RN	1280	4
GOTERM_MF_	GO:0036374	4	0.203355363	0.007290621	GGT5, GGT3	1280	6
GOTERM_MF_	GO:0003823	16	0.813421454	0.010640645	IGHM, IGLC	1280	103
GOTERM_MF_	GO:0032403	26	1.321809863	0.01307085	UFD1L, TMP	1280	206
GOTERM_MF_	GO:0044822	106	5.388917133	0.015035147	RPL30, RPL	1280	1129
GOTERM_MF_	GO:0003743	11	0.55922725	0.015645189	EIF4ENIF1,	1280	61
GOTERM_MF_	GO:0000400	5	0.254194204	0.017747102	RAD51B, YY	1280	14
GOTERM_MF_	GO:0000150	4	0.203355363	0.018195212	RAD51B, RA	1280	8
GOTERM_MF_	GO:0003676	93	4.728012201	0.020136829	ZNF572, RP	1280	985
GOTERM_MF_	GO:0052650	4	0.203355363	0.025776747	RDH12, RDH	1280	9
GOTERM_MF_	GO:0004126	4	0.203355363	0.025776747	APOBEC3F,	1280	9
GOTERM_MF_	GO:0016491	24	1.220132181	0.029494937	TSTA3, EGL	1280	200
GOTERM_MF_	GO:0043565	52	2.643619725	0.030867307	FOXA1, HNF	1280	518
GOTERM_MF_	GO:0047844	3	0.152516523	0.031044263	APOBEC3G,	1280	4
GOTERM_MF_	GO:0003700	88	4.473817997	0.045949751	FOXA1, ZNF	1280	961
GOTERM_MF_	GO:0004090	3	0.152516523	0.049165387	DHRS4L2, D	1280	5

Pop Total	Fold Enrichm	Bonferroni	Benjamini	FDR
16881	8.115865385	5.38E-08	5.38E-08	5.36E-08
16881	6.307438859	0.002957522	0.00148095	0.001476813
16881	3.804311899	0.023294712	0.007856688	0.007834742
16881	8.392542614	0.064369603	0.016633174	0.016586712
16881	7.91296875	0.384605258	0.097076329	0.096805166
16881	13.18828125	0.827977278	0.293114533	0.292295778
16881	8.7921875	0.999613667	1	0.998136067
16881	2.048665049	0.999989761	1	0.998136067
16881	1.664540352	0.99999927	1	0.998136067
16881	1.238226583	0.999999914	1	0.998136067
16881	2.378214652	0.999999956	1	0.998136067
16881	4.710100446	0.999999996	1	0.998136067
16881	6.594140625	0.999999997	1	0.998136067
16881	1.245187976	1	1	0.998136067
16881	5.861458333	1	1	0.998136067
16881	5.861458333	1	1	0.998136067
16881	1.58259375	1	1	0.998136067
16881	1.323920125	1	1	0.998136067
16881	9.891210937	1	1	0.998136067
16881	1.207667794	1	1	0.998136067
16881	7.91296875	1	1	0.998136067

KEGG

Category	Term	Count	%	PValue	Genes	List Tota	Pop Hits
KEGG_PATH	hsa05211:	12	0.61006609	0.006894107	EGLN3, TG	496	66
KEGG_PATH	hsa00603:	5	0.254194204	0.014835694	NAGA, A4G	496	14
KEGG_PATH	hsa05200:	41	2.084392476	0.015015852	MAX, PTGE	496	393
KEGG_PATH	hsa00480:	9	0.457549568	0.027863322	GGT5, GPX	496	51
KEGG_PATH	hsa03050:	8	0.406710727	0.035955315	PSMB11, P	496	44
KEGG_PATH	hsa05166:	27	1.372648704	0.041845365	ADCY4, PD	496	254
KEGG_PATH	hsa04668:	14	0.711743772	0.042649851	RIPK3, LI	496	107
KEGG_PATH	hsa04390:	18	0.915099136	0.042849916	FZD2, TGF	496	151
KEGG_PATH	hsa00600:	8	0.406710727	0.049118226	GALC, ARS	496	47

Pop Total	Fold Enrichm	Bonferroni	Benjamini	FDR
6879	2.521627566	0.853861259	1	1
6879	4.953197005	0.984317698	1	1
6879	1.446888082	0.985095101	1	1
6879	2.447462049	0.999612566	1	1
6879	2.521627566	0.999962067	1	1
6879	1.474258636	0.999993096	1	1
6879	1.814629183	0.999994534	1	1
6879	1.65325251	0.999994842	1	1
6879	2.360672615	0.99999917	1	1
